# Serial reproduction reveals the geometry of visuospatial representations

**DOI:** 10.1073/pnas.2012938118

**Published:** 2021-03-26

**Authors:** Thomas A. Langlois, Nori Jacoby, Jordan W. Suchow, Thomas L. Griffiths

**Affiliations:** ^a^Department of Psychology, University of California, Berkeley, CA 94704;; ^b^Department of Computer Science, Princeton University, Princeton, NJ 08542;; ^c^Computational Auditory Perception Research Group, Max Planck Institute for Empirical Aesthetics, 60322 Frankfurt am Main, Germany;; ^d^The Center for Science and Society, Columbia University, New York, NY 10027;; ^e^School of Business, Stevens Institute of Technology, Hoboken, NJ 07030

**Keywords:** visual perception, spatial memory, iterated learning, Bayesian statistics

## Abstract

An essential function of the human visual system is to locate objects in space and navigate the environment. Due to limited resources, the visual system achieves this by combining imperfect sensory information with a belief state about locations in a scene, resulting in systematic distortions and biases. These biases can be captured by a Bayesian model in which internal beliefs are expressed in a prior probability distribution over locations in a scene. We introduce a paradigm that enables us to measure these priors by iterating a simple memory task where the response of one participant becomes the stimulus for the next. This approach reveals an unprecedented richness and level of detail in these priors, suggesting a different way to think about biases in spatial memory. A prior distribution on locations in a visual scene can reflect the selective allocation of coding resources to different visual regions during encoding (“efficient encoding”). This selective allocation predicts that locations in the scene will be encoded with variable precision, in contrast to previous work that has assumed fixed encoding precision regardless of location. We demonstrate that perceptual biases covary with variations in discrimination accuracy, a finding that is aligned with simulations of our efficient encoding model but not the traditional fixed encoding view. This work demonstrates the promise of using nonparametric data-driven approaches that combine crowdsourcing with the careful curation of information transmission within social networks to reveal the hidden structure of shared visual representations.

## Significance

A primary function of human vision is to encode and recall spatial information about visual scenes. We developed an experimental paradigm that reveals the structure of human spatial memory priors in unprecedented detail. We ran a series of 85 large-scale online experiments with 9,202 participants that paint an intricate picture of these priors. Our results suggest a way to understand visuospatial representations as reflecting the efficient allocation of coding resources. In a radical departure from traditional theory, we introduce a model that reinterprets spatial memory priors as reflecting an optimal allocation of perceptual resources. We validate the predictions of the model experimentally by showing that perceptual biases are correlated with variations in discrimination accuracy.

The formation of accurate memories poses a difficult problem for the human visual system, which must process complex and noisy scenes while keeping pace with a relentless stream of incoming information. Because not all information is equally useful, the visual system must allocate its limited resources selectively, which leads to simplified and distorted internal representations (1–9). An essential function of the human visual system is to locate objects and navigate visual scenes, and understanding how it accomplishes this depends on detailed and accurate measures of visuospatial memory representations (10).

Previous work has probed visuospatial memory distortions using a task in which participants reproduced the locations of points within visual scenes, finding that participants’ responses were systematically biased (11–14). These systematic distortions have been described in terms of an attraction toward prototypical locations in the scenes (11–15), with perceptual attractors located at the centers of mass of visual objects (12); centered around prototype locations, such as the quadrant centers of a circle (11, 13, 14); or located along the medial axis (“shape skeleton”) of geometric shapes (16).

The state of the art in characterizing human visual memory biases relies on the long-standing category adjustment model (CAM) (11, 13), which asserts that each reconstruction R from memory linearly interpolates between the stimulus S and a prototype P, with

R=wS+(1−w)P+n [1]

for some weight w, where n is a perceptual noise term. Using the CAM relies on fitting the prototype location and other model parameters to the data, a process that is sensitive to estimation noise, particularly when using a relatively small number of human judgments (11, 13). In situations where multiple prototypes need to be estimated, the risk of overfitting to noise is even greater, and the number of prototypes must be predetermined (*Materials and Methods*).

**Fig. 1.**
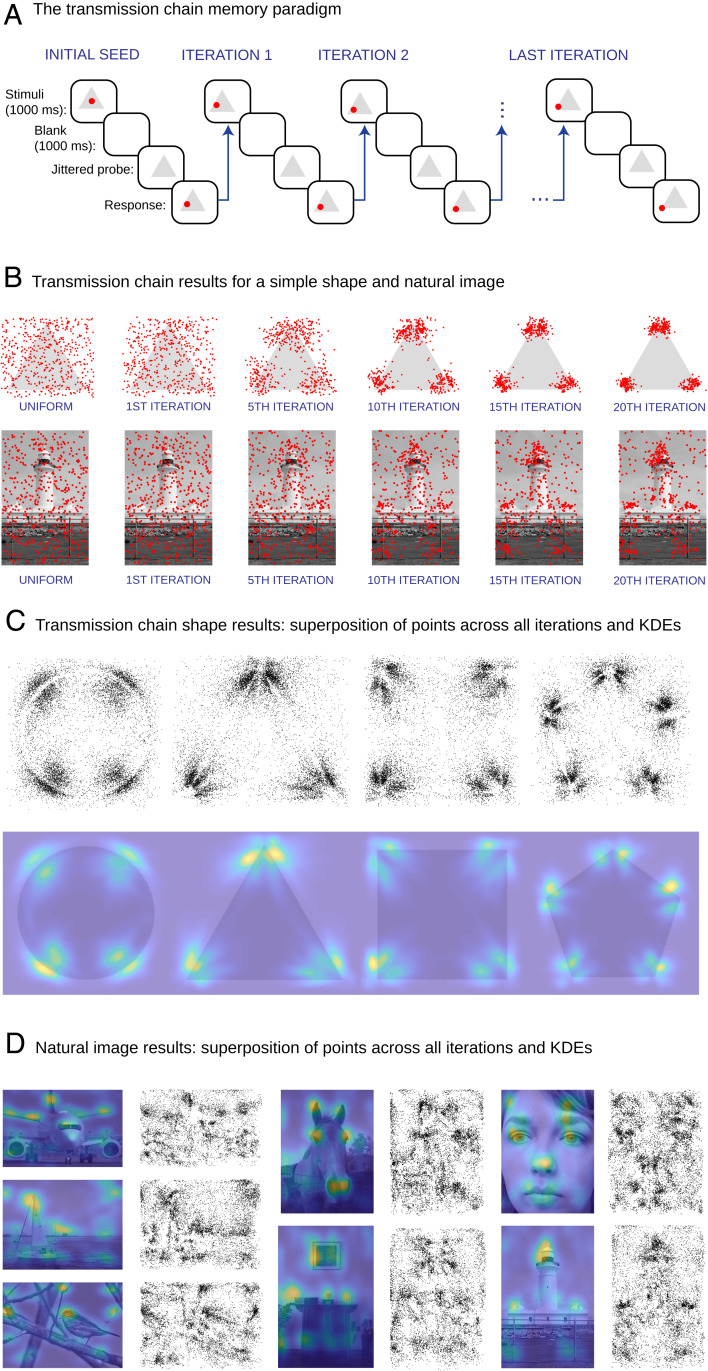
Visuospatial memory distortions, serial reproduction paradigm, and results. (*A*) Illustration of the serial reproduction method. The first participant views an image with a point overlaid in a random position and is then asked to reproduce its location from memory. The next participant views the same image but with the point located at the position reconstructed by the previous participant. The process is repeated for a total of 20 iterations. We adopted a between-subject design, where participants contributed to a given chain only once. (*B*) Serial reproduction results for the remembered position of points overlaid on a simple shape (triangle) and a natural image (lighthouse). The initial uniform distributions of 500 points are shown (column 1) as well as the distributions of the same points at iterations 1, 5, 10, 15, and 20 of the transmission chains. (*C*) Scatterplots showing the superposition of responses across all iterations of the chains for each of the shapes and the corresponding KDEs. (*D*) KDEs and scatterplots for complex natural scenes.

Here, we propose a method that overcomes these limitations. Our approach is based on two innovations. First, we leverage online crowdsourcing platforms to increase the number of human judgments obtained significantly, and second, we apply an adaptive sampling technique based on serial reproduction (17) to estimate the prototype locations nonparametrically, sidestepping any model-fitting approach. In our paradigm, information is repeatedly retrieved from memory by a sequence of people, with the reconstruction of one person becoming the stimulus for the next, forming a transmission chain analogous to the “telephone game.” The first participant views a point overlaid on an image and must later reproduce the location of the point from memory following a delay. The next participant views the same image but with the point located in the position reconstructed by the previous participant. This process is repeated for each participant in the chain (Fig. 1*A* and *SI Appendix*, Fig. S1). Unlike the traditional approach, which typically attempts to fit a descriptive model to noisy and unreliable estimates following only a single iteration of this process, we repeat it until convergence, which allows us to discern the prototypes toward which the responses converge. Intuitively, serial reproduction “amplifies” shared perceptual biases by compounding systematic errors (18). In terms of the cam, it is straightforward to show that repeating the paradigm eventually converges to the cam’s prototypes. In the simple case described in Eq. **1**, the distance to the prototype decreases on average with each iteration, and the prototype P is approximately a fixed point of the iterated process.

Indeed, in the case of simple shapes, our paradigm reveals a pattern of results that is consistent with previous literature and the cam (Fig. 1*C* and *SI Appendix*, Fig. S12) (13). However, it is also visually apparent that our technique paints a far more nuanced picture of visuospatial memory biases, revealing patterns missed by previous estimation approaches and that are inconsistent with a bias toward category centers (11, 12). Representative results are shown in Fig. 1 *B* and *C*. We found spatial memory distortions toward the edges and vertices of the geometric shapes, revealing a greater number of modes at different locations than previously thought (11–13). For natural images, the patterns are even more complex (Fig. 1*D*).

How can we explain the complex patterns of visual memory biases revealed by our method? The cam has traditionally been given a Bayesian interpretation (11). In this formulation, prototype point locations (landmarks) are replaced by a continuous probability density function [the prior p(S), which represents a belief state about probable point locations] where the landmarks correspond to the modes of the distribution. Intuitively, this distribution quantifies the degree of “landmarkness” of different visual regions. According to this view, participants infer point locations by combining noisy sensory information with the belief state. As a result, participants produce responses that are systematically biased toward nearby landmarks (*SI Appendix*, Fig. S2). The Bayesian interpretation has an important implication when it comes to understanding our serial reproduction paradigm because under experimentally verifiable assumptions, one can show that with multiple iterations of the serial reproduction process, distributions estimated from the chain results converge to the prior (refs. 18–20 and *Materials and Methods* have a proof).

**Fig. 2.**
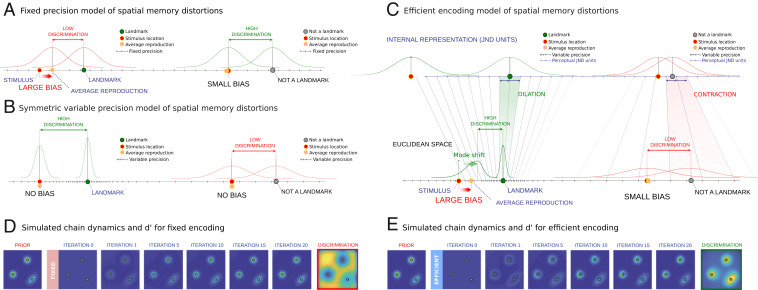
Models of visuospatial memory. The curves in *A–C* show the distributions of reproductions of a set of stimulus locations under a different model. (*A*) Fixed precision view. Perceptual noise (precision) is assumed to be constant, and biases occur when participants infer the true stimulus location (red dot). Average responses (pink dot) are pulled toward a nearby landmark (green dot). Because point locations near a mode (*Left*) are perceived to be closer to a nearby landmark, they are also harder to discriminate. Far from a mode (*Right*), responses are less biased, and discrimination between nearby point locations is higher. (*B*) Symmetric variable precision model. This model introduces the idea that perceptual noise (precision) varies and that it is highest near a landmark (*Left*) and lowest far from a landmark (*Right*). This model trivially explains higher discrimination accuracy near a landmark but does not predict perceptual biases. Individual responses are assumed to be independent noisy samples from a symmetric function centered on the true stimulus location, and on average, these responses will be unbiased (pink dot). (*C*) Efficient encoding model. Constant precision in a perceptually warped coordinate space (internal representation) determines how perceptual noise is skewed in Euclidean units. Visual regions near landmarks are overrepresented in the internal representation, resulting in higher precision in external Euclidean units. The skewed perceptual noise also predicts that responses near a landmark will be biased toward that landmark on average (*Left*) but not when the stimulus is far from a landmark (*Right*). In Euclidean space, the curves represent the reproduction distributions of the responses. The same reproduction distributions are also shown in the internal representation (in JND units). (*D*) Simulations of the fixed encoding model and predicted discrimination accuracy map. Given the prior (column 1), the model produces perceptual biases toward the three modes in the prior over multiple iterations of the serial reproduction process. Examples are shown for the 1st, 5th, 10th, 15th, and 20th iterations of the process. The fixed encoding model predicts that discrimination is reduced in the modes (column 8). (*E*) Simulations of the efficient encoding model and predicted discrimination accuracy map. The model also produces perceptual biases over multiple iterations. Critically, it also predicts increased discrimination accuracy in the modes of the prior (column 8).

Previous literature on cam (11) assumed that the sensory noise is Gaussian and isotropic regardless of location (“fixed precision”) with a fixed SD σ (Fig. 2*A*). This assumption is common to the classical “categorical perception” literature (21). Importantly, it has a direct mathematical implication with respect to how discrimination accuracy changes depending on the distance of a stimulus location to a landmark. In particular, it predicts that discrimination is lower near the landmarks because point locations near landmarks will be biased and perceived to be closer than they actually are, making them harder to tell apart (Fig. 2*A*). This phenomenon, known as the “perceptual magnet effect,” has been demonstrated in multiple perceptual modalities (21–24), including spatial memory (25, 26).

An alternative to the fixed precision view is the idea that precision varies over an image. The “variable precision” view trivially predicts variation in discrimination accuracy and can also explain convergence in the transmission chains since it models serial reproduction as a random walk with decreasing step sizes. With each step (iteration), responses are more likely to concentrate around the landmarks, which act as “absorbing states.” Critically, the variable precision view introduces the possibility that discrimination accuracy increases near the landmark, which is the opposite of the prediction made by the fixed precision model. In this paper, we test these opposing theoretical predictions empirically. Testing for the perceptual magnet effect or an increase in discrimination accuracy near a landmark is accomplished by comparing the results of the memory experiments (which reveal biases in reproduction) with the results of two-alternative forced choice (2AFC) “same” or “different” experiments, which quantify discrimination accuracy. We show that discrimination accuracy is higher near the landmarks, refuting the long-standing fixed precision account of spatial memory biases and supporting the variable precision view.

However, variable precision may or may not predict consistent perceptual biases. In its simplest form, variable precision can be implemented with symmetric noise (“symmetric variable precision”) (Fig. 2*B*). Such a model predicts that R−S (a single-trial response) has independent noise with decreasing magnitude closer to a landmark. However, this prediction is at odds with the fact that people tend to produce biased responses (11, 12, 25) and that as a result, neighboring point reconstructions tend to be oriented in similar directions. For example, nearby point locations that are close to a landmark will be consistently shifted toward that landmark (*SI Appendix*, Fig. S3). We confirm this effect in our data, which we measure by quantifying the probability of small angular differences in single-trial biases for nearby point reconstructions, indicating that the variable precision model is ill suited to fully explain spatial memory distortions, at least in its simplest form.

These results demonstrate the need for a theoretical model based on the variable precision view that can also predict the consistent perceptual biases in the data. In this work, we innovate on a recent Bayesian formulation of variable precision developed in terms of efficient encoding (7), generalizing it to the high-dimensional case using mathematical tools from differential geometry. According to this model, convergence of the serial reproduction chains occurs due to the combined effect of variable precision (causing a shift in successive reproductions toward the landmark) and consistent perceptual biases (like a “gravitational pull” of responses toward the landmarks). This model has the advantage of being a fully Bayesian model, just like the long-standing cam account of spatial memory described above, and with no additional parameters. To explain this model, we start with a key notion from signal detection theory (27).

A common assumption from signal detection theory is that variable precision over an image can be measured both in terms of changes in sensitivity using physical (Euclidean) distance units and also, in terms of constant just noticeable distance (JND) units over a transformed internal representation of the space (Fig. 2*C* and *SI Appendix*, Fig. S4). In other words, increased precision in a Euclidean coordinate space is equivalent to constant precision in a perceptually dilated coordinate space. Intuitively, the geometric pattern of dilations and contractions is similar to how variations in perceptual sensitivity are reflected in neural representations such as the somatosensory homunculus (28) or retinotopic map (29), where increased precision corresponds to areas that are overrepresented by the brain. Interpreting variable precision in terms of JND units is useful because it forms the basis of a fully Bayesian formulation of the variable precision view that overcomes its limitations when it comes to predicting perceptual biases while also predicting increased discrimination accuracy near the landmarks (Fig. 2 *C* and *D*) (7, 30).

**Fig. 3.**
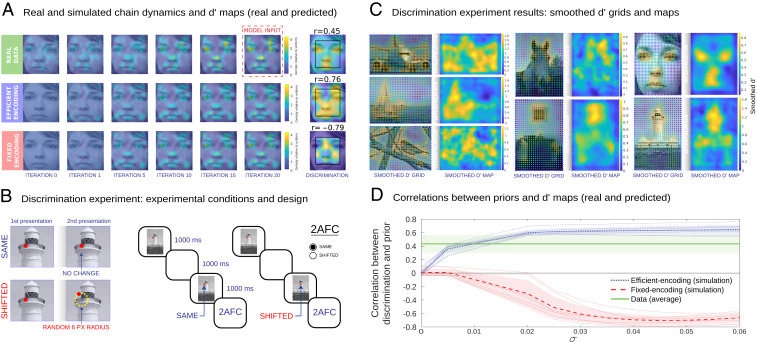
Visuospatial memory distortions correspond to variable encoding precision. (*A*) Representative example of real and simulated chain dynamics and discrimination maps (face image). Real and simulated KDEs are shown for iterations 0, 1, 5, 10, 15, and 20. Both the efficient and fixed encoding models provide good approximations to the real transmission chain data (*SI Appendix*, Fig. S8). Real and simulated discrimination accuracy maps are also shown, including correlations to the prior. (*B*) Discrimination experiment conditions and experimental design. Discrimination points were obtained from a regular two-dimensional (2D) grid of points over the image. In the same condition, the red dot did not change position in the second presentation. In the shifted condition, the red dot was shifted to a point located in a random position at a six-pixel radius distance from the original position. Two identical images were shown for 1,000 ms sequentially with a red dot placed on top of them. The dot was either in the same location in both cases (same condition) or shifted (shifted condition). Both the dot and the image were shifted by a random offset in the second presentation in both conditions. The starting points were sampled from a 2D grid of possible points over the image. (*C*) Discrimination results for natural images. Discrimination $d^{\prime }$ values for each grid point were convolved with a Gaussian kernel, and final maps were computed through cubic interpolation of the smoothed $d^{\prime }$ grid values. (*D*) Correlations between priors and discrimination (natural images). For each noise magnitude σ, we computed the correlation predicted by the two models. The correlations were positive (blue line) for the efficient encoding model and negative (red line) for the fixed encoding model. Thin lines show data for individual natural images; error bars show SDs across images. The green line shows the mean and SD of the correlations of the empirical $d^{\prime }$ data and the priors. We exclude the edges of the images because the fixed encoding model produces predictions with noticeable edge artifacts resulting in slightly smaller correlations than the ones we report. The fixed encoding model also predicts smaller variation in $d^{\prime }$ across the images (*SI Appendix*, Fig. S8). The data support the efficient encoding model.

The efficient encoding model (7) is based on the idea that encoding resources limit the ability to store all regions of a visual scene with equal accuracy, and it specifies the optimal trade-off between coding resources and precision (7, 30). The essence of the model is that it determines the exact mathematical relation between the magnitude of the bias and discrimination accuracy. This is useful because it predicts the full range of empirical results in this paper including the serial reproduction dynamics and discrimination accuracy measures (Figs. 2 *D* and *E* and 3 *A* and *D*). Critically, it also predicts that single-trial biases for nearby point reconstructions tend to point in the same direction (*SI Appendix*, Fig. S3).

Thinking about spatial memory distortions in terms of efficient encoding helps to explain the structured priors revealed by our method: As the perceptual space is condensed to Euclidean space, it concentrates the prior probability distribution in regions of greater precision (Fig. 2*C*). A uniform prior in the perceptual space will become a distribution in Euclidean space in which probability is proportional to encoding precision. As a result, the priors we estimate reveal the geometry of the perceptual space. This perspective also makes additional testable predictions. Because it explains biases in terms of an optimal allocation of encoding resources, it predicts that limiting these resources in the task should result in qualitative changes to the internal representation for a given stimulus image, rather than just introducing additive noise to the original representation. We confirm this prediction empirically by reducing the encoding time in our experiments, which reveals qualitative simplifications to the transmission chain results, rather than just additional noise. In contrast, changing the retention time or manipulating the display during the reproduction phase had only a minor effect on the final results, suggesting that biases emerge during encoding rather than the retention or reproduction phases.

## Results

### Revealing Spatial Memory Priors by Serial Reproduction.

We began by running a series of serial reproduction experiments probing memory for point locations in simple images and a selection of complex natural scenes. For simple images, we used geometric shapes (circle, triangle, square, and pentagon), and for natural scenes, we used images of both natural and man-made objects (Fig. 1). We ran approximately 500 unique chains, 1 for each initial point location, which we randomly sampled from the uniform distribution. For each chain, the telephone game was played for 20 iterations. Fig. 1*B* shows the initial uniform distributions of the points for the triangle and a natural image, as well as the results of the 1st, 5th, 10th, 15th, and 20th iterations of the process. As expected, initial point locations and the locations of points in the first iteration were not significantly different from a uniform distribution (*P* = 0.35 and *P* = 0.08 for initial seeds and iteration 1, respectively). However, subsequent iterations deviated considerably from the uniform distribution (*P* < 0.001 for iterations 2 to 20 for all shapes). The distributions estimated based on data aggregated from each iteration converged to a stationary distribution within approximately 20 iterations (*SI Appendix*, *SI Text* and Figs. S5 and S6 have further convergence analyses). Fig. 1 *C* and *D* shows scatterplots of the chain point locations across all iterations for each of the images, as well as kernel density estimates (KDEs; which are estimates of the underlying distributions that produced the data, as explained in *Materials and Methods*). They reveal the intricate structure of visuospatial memory priors.

### Precision Shapes Visuospatial Memory Representations.

To test the opposing predictions of the fixed and efficient encoding models (the simulated $d^{\prime }$ results of both models are in Fig. 2 *D* and *E*), we ran a series of discrimination accuracy experiments on a separate cohort of participants using the same images. Participants saw the image with a point positioned over it in a random location sampled from a regular grid of possible point locations (Fig. 3 *B* and *C*). After a 1,000-ms delay, the same image reappeared with the point in either the same position or in a shifted position, and participants were asked to determine if the point was the same or “shifted” (Fig. 3*B*). We obtained change sensitivity responses from dense point grids over our images, producing detailed $d^{\prime }$ accuracy maps (Fig. 3*C* and *SI Appendix*, Fig. S7). Smoothed $d^{\prime }$ accuracy maps are shown for the natural images in Fig. 3*C*. We found that discrimination maps were consistently highly and positively correlated with the transmission chain results. Because the discrimination maps and prior KDEs are estimated from noisy empirical measurements, we also computed disattenuated correlations between the priors and the $d^{\prime }$ maps using estimates of the internal reliability of the two measures (*SI Appendix*). We found that the disattenuated correlations for the $d^{\prime }$ maps, which ranged between *r* = 0.76 and *r* = 0.93 (average *r* = 0.82), predict a significant portion of the variance, even though there may still be some systematic variation originating from other sources. Note that given the prior, both models have only a single degree of freedom (the perceptual noise σ). While both models fit the transmission chain experiment dynamics well (Fig. 3*A* and *SI Appendix*, Fig. S8), the results of the discrimination experiment are consistent with the predictions of the efficient encoding model (we observed positive correlations between the transmission chain results and the simulated discrimination accuracy maps in all cases; *P* < 0.001; via bootstrapping) but not the fixed encoding model (we observed negative correlations in all cases where σ>0). Fig. 3*D* shows these opposing predictions.

### Consistent Perceptual Biases.

A well-documented finding in the literature describes people’s tendency to produce consistent perceptual biases in the task. The bias consists in producing a response that is oriented toward the nearest landmark. One implication of this is that reproductions of neighboring point locations will tend to be oriented in similar directions toward the nearby landmark. However, the symmetric variable precision model predicts random independent bias directions (*SI Appendix*, Fig. S3 *A* and *B*). To quantify this effect in our data, we computed histograms of the angular differences between the averaged biases of nearby point reconstructions for the triangle data (*SI Appendix*, Fig. S3*F*) and for all natural images (*SI Appendix*, Fig. S3*G*). We found that small angular differences (between −12° and +12°) tend to be 1.83 to 4.36 (mean 2.8) times more probable than expected by chance for all images (*P* < 0.001; via bootstrapping) (*SI Appendix*, Fig. S3). The efficient encoding model predicts significantly more probable angular differences in this range, while the symmetric variable precision model predicts a uniform distribution of angular differences (*SI Appendix* has more information).

### Encoding, Memory, and Reproduction.

The theoretical assumptions of the efficient encoding model predict that distortions should change with direct experimental manipulations of encoding precision (7). We confirmed this through controlled experiments in which we manipulated both spatial and temporal factors of encoding precision for one of our natural images. Specifically, we manipulated encoding precision temporally by reducing the encoding time in our task from 1,000 to 200 ms. We also manipulated encoding precision spatially by either adding Gaussian noise to the stimulus image or reducing its contrast significantly. We found that these manipulations produced priors that were significantly different from the original when we compared the resulting KDEs with the original findings (*P* < 0.001) (*SI Appendix*, *SI Text* and Fig. S9 have details). We also observed simplifications to the priors for shape images following similar experimental manipulations (*SI Appendix*, Fig. S11). In addition, we tested if the biases are generated during the encoding phase or if they emerge during the testing phase of the experiment when the image is reintroduced and participants produce a response. To do this, we substituted the image during the reproduction phase with a completely blank gray region, as well as the opposite: a blank gray region during the encoding phase, followed by a naturalistic image during the reproduction phase. If biases originate from visual processing of the images during the reproduction phase, we would expect to see biases that reflect the visual characteristics of the images shown at test time (e.g., the natural image if the image shown during the encoding phase was a blank gray region). However, we find the opposite: The pattern of biases corresponds to the visual characteristics of the image presented during the encoding phase and not the reproduction phase (*SI Appendix*, Fig. S10). Finally, we tested the effect of increasing the delay time (from 1,000 to 2,000 ms) and found that it did not produce any significant differences in the pattern of biases when compared with the original results (*SI Appendix*, Fig. S9), suggesting that the visuospatial information is preserved throughout the delay phase.

## Discussion

### Summary of the Results.

We developed an experimental paradigm that provides direct estimates of the geometry of visuospatial representations. We achieved this by adopting a spatial memory task (remembering the location of a point in an image) and incorporating it into transmission chains. Using this iterative paradigm, we show that visuospatial distortions are far more intricate and complex than previously suggested (Fig. 1). The traditional view formulated in terms of the cam holds that perceptual biases are due to an attraction toward prototypical landmarks in a scene. This view typically assumes fixed precision regardless of location. As a consequence, it predicts lower discrimination near landmarks (Fig. 1*D*). We tested this prediction empirically and found the opposite (Fig. 3). These results are consistent with a variable precision theory—namely, that biases are due to reduced perceptual noise near landmarks. We formalized these two interpretations in terms of Bayesian models and found that although both predict the biases and chain dynamics well, only the efficient encoding model (which is the Bayesian incarnation of the variable precision view) accurately predicted the discrimination results. We also show that the efficient encoding model, unlike an alternative non-Bayesian variable precision model, has the added benefit of predicting the consistent perceptual biases that are clearly present in the data and that have been reported in past work as well (11, 25). Furthermore, by manipulating the images shown during the encoding and reproduction phases of the experiment, we demonstrate that biases emerge during the encoding phase rather than during the delay or reproduction phase. We also show an interaction between the visual complexity of landmarks and encoding time: Shorter encoding times result in simplified internal representations (*SI Appendix*, Figs. S9 and S11). Both results are aligned with key predictions of the efficient encoding model, namely that biases emerge during the initial process of encoding spatial locations with respect to the image, rather than during memory retention or reproduction.

### CAM.

Previous work explains distortions as a consequence of being drawn to perceptual attractors. In this tradition, broadly referred to as the CAM, two distinct approaches have been taken to characterizing these attractors. The first approach asserts that perceptual attractors (or “prototypes”) are located at object centers (12, 31, 32). Object centers have typically been operationalized as the centers of mass of handcrafted semantic segmentations of images. We found that centers of mass were poor predictors of the priors revealed by the transmission chains, with an average correlation to the priors of *r* = 0.22 across all our primary images (*SI Appendix*, Fig. S15), as well as a representative sample of images used in prior work (12, 31) (*SI Appendix*, Fig. S16). In the second approach, prototypes are estimated using a descriptive model that asserts that each reconstruction R from memory linearly interpolates between the stimulus S and a prototype P (Eq. **1** and *Materials and Methods*). Previous work has typically estimated prototype locations by relying on a small number of experimentally observed reconstructions (11–13). This approach provides good pointwise approximations to the end result we measure in our paradigm for simple shape images (14) (*SI Appendix*, Fig. S12). However, in the case of natural scenes, where the number of modes is large and hard to estimate, this approach is prone to overfitting and produces mostly crude pointwise approximations of the distributions (*SI Appendix*, Fig. S13). Finally, a bootstrapping analysis indicates that using the cam fit to the data in the first iteration of the chains cannot produce estimates of the modes in the prior that are as reliable as those obtained using serial reproduction, even when equating the amount of data used by both methods in the comparison (*SI Appendix*, Fig. S14). These results demonstrate the practical advantages of our approach over estimation procedures that rely on parametric model fitting.

### Are Visuospatial Representations Low Level?

It is natural to ask if our results can be explained away using low-level features of the images. If internal representations are indeed more than a simple function of low-level features, we might expect to see biases anchored around regions that are physically absent and only implied by contextual information. We tested this prediction by repeating the transmission chain experiments using images possessing illusory contours (*SI Appendix*, Fig. S17). Illusory contours included a gray square with a smooth gradient that erased its upper right-hand corner entirely, as well as an image of a face in which a gradient erased its right half, with the other half implied by symmetry and context. Transmission chain results revealed biases concentrated around the illusory regions: a pattern around the upper right-hand illusory corner of the square that is largely identical to the pattern we observed with the original image, as well as biases centered over the illusory eye in the face image (*SI Appendix*, Fig. S17).

We also completed an additional manipulation in which we used human segmentation data of the images and replaced the entire textured images with uniform gray-scale regions corresponding to the segmented visual objects (*SI Appendix*, Fig. S15). Despite the removal of all of the fine structure, we found that the resulting KDEs are among the most predictive of the original findings, suggesting that semantic information rather than low-level textural information is responsible for a significant portion of the variance, with an average correlation across all images for which the semantic segmentations were available of *r* = 0.57. Finally, these experimental findings are in line with the results of additional supporting analyses (*SI Appendix*, Fig. S17) indicating that the presence of modes in the KDEs is not strictly a function of low-level information in the images, such as corners and edges extracted using classic image feature detectors (33).

### Attention.

We propose the efficient encoding theory as a Bayesian model that explains visuospatial distortions in terms of systematic variations in encoding precision. There are several physiological and neural processes that may support this process. For example, it is natural to speculate about whether precision and spatial memory are mediated by attention in our task, including overt attention in the form of eye movements (34).

To address this, we ran several controlled experiments in which we combined shorter encoding times (300 ms) with forward and backward noise masking. While shortening the encoding time caused notable simplifications in the structure of the priors, which is consistent with the view that biases are due to variations in encoding precision (both spatial and temporal), the presence of forward and backward masking had little to no effect (*SI Appendix*, Fig. S10). However, another possibility is that spatial memory priors reflect spontaneous patterns of free fixations over image regions and that these regions become spatial memory landmarks by virtue of being encoded with higher precision following sustained foveation. However, we found that patterns of free fixations were poor predictors of our original results (*SI Appendix*, Fig. S15).

Next, we tested an additional option: It is well known that overt attention can vary substantially according to the visual task (34) and that although free fixations might not be predictive of visuospatial memory priors, perhaps that fixation patterns produced by participants engaged in a different task might. In order to address this question, we repeated our experiments using images for which fixation patterns were available not just for free fixation but for cued object search and saliency search tasks as well (35). Although we found the fixation maps to be highly intercorrelated (*SI Appendix*, Fig. S18), none provided good predictions of the spatial memory priors obtained using our paradigm (average *r* < 0.2 in all cases, even with optimal smoothing and correction for attenuation) (*SI Appendix*, Fig. S19). In addition, we found that our KDE results were also not highly correlated with explicit measures of image regions obtained using a recent behavioral patch rating procedure known to be predictive of overt attention (36, 37) (*SI Appendix*, *SI Text* and Fig. S19 have details). These results suggest that overt attention only explains part of the variability in spatial memory priors, although we cannot completely rule out that unique eye movement patterns specific to our task could be mediating precision and bias, despite the fact that our noise masking experiments suggest otherwise. Further work is required to understand how attention is involved and whether additional mechanisms mediate how spatial memory representations are encoded, such as explicit verbal strategies (i.e., verbal descriptions of image regions to estimate locations).

### Modeling Assumptions.

Our experimental method is nonparametric in that it does not rely on model fitting. However, our interpretation of serial reproduction does rely on a number of experimentally verifiable assumptions. We assume that participants possess similar perceptual priors and that they perform the experiment by relying solely on the point location presented to them in a given trial (a Markovian assumption). These two assumptions are traditionally verified in experiments using transmission chains by way of a strictly within-participant design, in which each chain contains data from only a single participant (20, 38, 39) (*SI Appendix*, Fig. S1). We show the results of this within-participant design for one of our shape images and a natural image in *SI Appendix*, Figs. S20 and S21. The results are similar to the original findings, although the original results are less noisy, in line with previous work studying the effects of collective behavior on perception and decision making (40). Therefore, we opted to present the results of the fully between-subject design as our main findings. However, it is possible that individual differences exist with respect to the relative strength of different landmarks within a given image and that if this is true, the between-subject design we adopted cannot reveal this. We illustrate the results of a fully within-participant serial reproduction design, which can be used to detect individual differences with more data from each participant. However, further work will be required to fully characterize the role of individual differences.

We further tested the Markovian assumption by adding uniform dummy trials in between experimental trials in all of the chains, where the image was shown with a point in a random location rather than the location produced by the previous participant in the chain. Had participants relied on information carried over from previous trials, this manipulation would have produced a significant effect. However, we found that this manipulation had only a minor and nonsignificant effect on the results, supporting the validity of the Markovian assumption (*SI Appendix*, Fig. S9). Note that we used 20 iterations to estimate the prior based on several metrics (*SI Appendix*, Figs. S5 and S6), which reveal that convergence of the KDEs occurs by 20 iterations and that adding iterations to the chains did not alter the estimated distributions substantially (*SI Appendix*, Fig. S22). However, it is also visually apparent that there is some variation between images, so it is possible that results could be improved with additional iterations, although that would also come at the cost of completing longer and more data-intensive experiments.

Finally, while our method reveals more intricate structure than previous methods (Fig. 1), it is conceivable that even more refined details could be extracted either with more data or with more sophisticated data aggregation methods, such as averaging data over multiple participants before transmission to the next participant in a chain (41). In addition, we make a simplifying assumption in our modeling by not considering the possibility that additional reproduction noise may be contributing to the biases, but modeling reproduction noise would not change the qualitative nature of the relation between discrimination accuracy and biases (ref. 20 has a simulation that takes production noise into account in an auditory reproduction task).

### Bayesian Inference and the Efficient Encoding Model.

Our empirical findings are consistent with a variable precision interpretation of visuospatial biases, which predicts that chaining responses in the spatial memory task will result in a shift toward high-precision areas that act as absorbing states. According to this view, convergence in the chain is due to skewed perceptual noise toward the landmarks. However, the simplest form of the variable precision account might be to view the iterative process as an unbiased random walk, where step size decreases with lower perceptual noise, without perceptual biases (in other words, individual point reconstructions will not necessarily consistently point toward a nearby landmark). We favor a more complex version of the variable precision account that innovates on a recent Bayesian formulation of variable precision based on efficient encoding (7). The differences between these models are illustrated in Fig. 2. We see both empirical and theoretical arguments in support of a Bayesian interpretation and the efficient encoding model in particular.

First, unlike a simple variable precision account, the efficient encoding model predicts consistent perceptual biases. These biases correspond to the well-documented finding (11, 25) that people tend to produce responses that are consistently oriented toward the nearest landmark. We confirm this effect in our data (*SI Appendix*, Fig. S3 *C* and *G*) and also show that the efficient encoding model captures this effect (*SI Appendix*, Fig. S3 *C–H*), unlike a simple variable precision model, which completely fails to do so (*SI Appendix*, Fig. S3 *B–H*).

Second, there is a large body of work on spatial memory that explains systematic biases in terms of the cam. This work typically uses Bayesian inference to describe spatial memory (11, 25), so it is natural that our modeling approach should adopt the same formalisms. In addition, the Bayesian approach provides a unified account for describing multiple perceptual tasks and specifies clear and testable predictions regarding the precise mathematical relations between them, such as the relation between the magnitude of the biases and discrimination accuracy.

Third, earlier non-Bayesian incarnations of the cam describe perceptual attractors in terms of discrete prototypes rather than continuous distributions. However, our data clearly reveal modes that vary systematically in terms of their density, elongation, orientation, and shape, all characteristics that are hard to describe using a model that can only produce discrete pointwise categorical estimates (Fig. 1*D*). This is especially evident in the case of natural images, where fitting a large number of discrete modes provides a significantly poorer approximation of the biases compared with a baseline (*P* < 0.01 for all images) (*SI Appendix*, Fig. S13). By contrast, the Bayesian formulation overcomes this problem by describing perceptual representations in terms of continuous distributions rather than discrete pointwise entities.

Fourth, unlike a non-Bayesian variable precision account, the efficient encoding model provides a useful theoretical motivation for why encoding precision is higher in some visual regions and not others. Because it explains biases in terms of an optimal allocation of encoding resources, it makes a number of theoretical commitments that are both testable and useful for understanding perceptual biases. First, it predicts that biases emerge during encoding, rather than delay or reproduction. Therefore, it predicts that manipulating encoding resources directly should interact with the structure of the biases. In fact, when we manipulated encoding time, we observed a structural simplification in the complexity of the results. An unconstrained variable precision model does not provide any theoretical motivation for why decreasing encoding time would generate anything beyond increased additive noise, let alone a qualitative shift toward a simplified representation (*SI Appendix*, Fig. S11*B*). We observed a similar simplification using a spatial manipulation of visual complexity (*SI Appendix*, Fig. S11*A*).

Finally, in addition to predicting consistent perceptual biases, the Bayesian models provide a good fit to the dynamics of the serial reproduction chains. Fig. 2*A* and *SI Appendix*, Fig. S8 provide the results of additional self-consistency tests of the efficient and fixed encoding models in terms of how closely they approximate the complex chain dynamics of the serial reproduction data for one of our images. We show that using the data from the last iteration of the serial reproduction experiments can predict the rate of convergence and the dynamics of all previous iterations (after fitting the noise-magnitude parameter to the data) and in the case of efficient encoding, predicts the positive correlations between discrimination results and priors estimated from the serial reproduction experiment (Fig. 3). This supports the idea that in addition to predicting perceptual biases, the efficient encoding model produces good approximations to the perceptual distortions and discrimination accuracy measures, as well as the dynamics of the transmission chain results in our task.

However, as with any Bayesian model that invokes a “prior” and a “likelihood,” there comes a need to make a number of interpretative commitments that are worth discussing here. First, it is clear that any theory of spatial memory should somehow capture and quantify the concept of a “landmark” because it is a key concept in spatial memory. One could describe landmarks as discrete pointwise entities (along the lines of the cam in its descriptive non-Bayesian form), but our empirical data show that using a fixed number of discrete pointwise estimates is not sufficient to capture the behavioral results, which reveal graded continuous patterns (with varying elongations, orientations, and aspect ratios). We provide some quantitative evidence for this (*SI Appendix*, Fig. S13), but it is also visually apparent that there tends to be many landmarks particularly in complex scenes and that they are not discrete. Therefore, it is natural to quantify the concept of a landmark as some continuous function that determines the degree of landmarkness of visual regions in a scene. The prior accomplishes this since it is a continuous function that assigns a higher value to visual regions that are more landmark like (because landmarks make it easier to encode nearby locations). This function is also a probability density function, which lends it an additional interpretation in the Bayesian formulation: It is a belief state about probable point locations in a visual scene. However, even without adopting a Bayesian interpretation, it is clear that some kind of continuous function p(s) is needed in order to specify the degree of landmarkness of visual regions.

Another component of almost any theory of perception is some way to encapsulate the notion of perceptual noise. In other words, how accurately or noisily is a given point location perceived by an observer? Assuming that the noise is fixed regardless of location results in predictions that are incompatible with our discrimination accuracy data (this is the fixed precision model, illustrated in Fig. 2 *A* and *D*). As a result, we need to come up with some function that captures the idea that perceptual noise varies systematically from location to location in an image (variable precision). In Bayesian terms, the likelihood function is ideally suited to play this role. Again, even without adopting a Bayesian view of this idea, the concept of a continuous function that captures the degree to which perceptual noise influences spatial memory remains useful. The Bayesian account only specifies how the prior and likelihood are combined mathematically to form the posterior during inference. In this work, we assume that reproductions are a sample from the posterior, although previous work discusses alternatives to this, such as maximum a posteriori estimation, which models reproduction as the mode of the posterior rather than a sample (18). According to our model, the reproduction distribution is the net result of the encoding (determined by the likelihood) and decoding process (determined by the posterior). The chaining of these two processes results in the observed reproduction distribution p(R|S) (*SI Appendix*, Fig. S2).

Nevertheless, adopting the Bayesian interpretation comes at a cost: It is significantly more complex mathematically, although it does not introduce any additional degrees of freedom over a naive model where variable precision is given by an arbitrary noise term. Specifically, both the Bayesian and non-Bayesian formulations of variable precision depend solely on a scalar function defined over the entire space [σ(s) in the case of a variable precision model and p(s) in our case]. All of the predictions made by our model (e.g., the discrimination maps and chain dynamics) are determined only from this scalar function.

### Conclusion.

Exploring spatial memory biases using serial reproduction demonstrates that the study of shared perceptual representations can be approached by recasting experimentation as algorithm design and through the lens of information transmission inside carefully curated social networks. More broadly, this work demonstrates the benefit of bringing innovative experimental and psychophysical methods and computational statistics to bear on our understanding of otherwise hidden internal representations. The advantage of this approach lies in fully characterizing the structure of internal representations, revealing rich, complex, and ecologically valid perceptual spaces. This detailed understanding can spur theoretical insights with respect to how perceptual systems encode and process sensory information.

## Materials and Methods

### Participants.

Participants were recruited online using Amazon Mechanical Turk. The experiments were approved by the Committee for Protection of Human Subjects at the University of California, Berkeley and the Institutional Review Board at Princeton University. We obtained informed consent from all volunteers. *SI Appendix*, Fig. S23 presents the exact number of participants in each of the 85 experiments. The overall number of participants in all experiments was 9,202.

### Stimuli.

The images used in the transmission chain experiments were gray-scale images of a few simple shapes (circle, triangle, square, and pentagon), as well as gray-scale images of natural scenes. A detailed description of the images is provided in *SI Appendix*. *SI Appendix*, Fig. S23 shows the list of image file names for each of the experiments. All stimuli for the experiments are available in our open science repository (42).

### Procedure.

Transmission chain memory experiments were programmed using the Dallinger platform for laboratory automation for the behavioral and social sciences (43). Reproducible code for the Dallinger experiments is provided in the open science repository. Patch ratings experiments and discrimination experiments were programmed using the Amazon Mechanical Turk application programming interface (API).

#### Transmission chain memory experiments.

Participants were shown an image with a point overlaid on it for 1,000 ms (Fig. 1*A*). The initial point locations were sampled from a uniform distribution. Participants were asked to reproduce the position of the point as accurately as possible following a 1,000-ms delay, when the image reappeared on the screen without the point. To prevent participants from resorting to marking the absolute positions of the points on the screens during the task, the displays were shifted by a random offset on the screen during the stimulus phase and the probe (Fig. 1*A*). The response was then sent to another participant who performed the same task. A total of 20 iterations of this telephone game procedure were completed for each chain. We terminated each experiment after approximately 12 h. The number of total chains varied somewhat between experiments (mean 465, range 250 to 577 chains) (*SI Appendix*, Fig. S23). A typical experiment included 105 trials, and the average time needed to complete the task was about 12 to 14 min. *SI Appendix*, Fig. S23 presents the number of participants in each experiment. *SI Appendix* has additional details.

#### Visual discrimination experiments.

Participants saw an image presented for 1,000 ms with a red point overlaid on it (Fig. 2*B*). Following a 1,000-ms delay with a blank screen, the image reappeared with the point either in the same exact location relative to the image or in a shifted position (both the durations of the display and the gray-scale images were identical to those in the transmission chain experiments). In the shifted condition, the shifted point was offset by a six-pixel radial distance from the original point location, sampled uniformly along the circumference of the circle defined by the six-pixel radius centered at the original point location. In all cases, the overall display (the image and point) was shifted by a random offset in the second presentation to prevent participants from using absolute positions within the display. The second display remained for 1,000 ms on the screen and was followed by a 2AFC (“red point same” or “red point shifted”). *SI Appendix* has additional details, including the 2AFC data analysis. We obtained responses from a total of 20 participants for each grid point and for each condition (same or shifted).

### Nonparametric KDE.

For each chain, we used the data for all iterations. We computed the empirical mean and covariance matrix [$\mu_{i}={mean}_{j}\left(R_{ij}\right)$, $\Sigma_{i}={Cov}_{j}\left(R_{ij}\right)$], where $R_{ij}$ is the response in chain i and iteration j. To estimate the typical kernel width, we computed the square root of the eigenvalues of this matrix. These values ranged between 0.015 and 0.025 for shapes and between 0.020 and 0.040 for images (these values are reported in units of fraction relative to an image size of 1). Since the covariance estimate is based on a small number of points, we computed a regularized covariance matrix $\Sigma_{i}^{\prime }=\Sigma_{i}+\lambda^{2}I$ where λ was set to 0.015 for shapes and 0.020 for natural images and I is the identity matrix (values were chosen based on the estimates of the unregularized matrices above). For each chain, we computed a Gaussian distribution: $p_{i}\left(s\right)=\frac{1}{\sqrt{{\left(2\pi \right)}^{2}|\Sigma_{i}^{\prime }|}}\exp\left(-\frac{1}{2}{\left(s-\mu_{i}\right)}^{T}\Sigma_{i}^{\prime -1}\left(s-\mu_{i}\right)\right)$. Next, we computed the KDE as the normalized sum over all of the $p_{i}$ distributions. If N is the total number of chains, the nonparametric KDE for a given image becomes $P\left(s\right)=\frac{1}{N}\sum_{i}p_{i}\left(s\right)$. Results of this procedure are shown for the shape image results in Fig. 1*C*.

### Parametric KDE.

KDEs were computed using the data from the last iteration of the chains. For each point, we computed a Gaussian kernel centered at the point with a diagonal covariance matrix. We set the kernel width to a conservative value of 0.025 for shapes and 0.040 for natural images. These values were chosen based on the ranges of the estimates obtained from the unregularized nonparametric kernels. The final KDE was calculated by summing all of the Gaussian kernels and normalizing. Results of this procedure were used for all statistical analyses.

### Comparing Nonparametric Estimates with the CAM.

The CAM (13) describes the remembered position for a response vector i as a weighted average of the actual location at which the point was presented ($S_{i}$) and the weighted sum of the M spatial attractor locations using the following equations:

$$R_{i}=wS_{i}+\left(1-w\right)\overset{M}{\underset{k=1}{\sum }}v_{ik}P_{k}$$

where

$$v_{ik}=\frac{e^{-c∥S_{i}-P_{k}∥}}{\sum_{k^{\prime }=1}^{M}e^{-c∥S_{i}-P_{k^{\prime }}∥}}$$

and $R_{i}$ and $S_{i}$ are vectors in $R^{2}$ containing the two coordinates for the *i*th initial seed point (in iteration 0) and the corresponding *i*th response point in iteration 1, respectively. The $P_{k}$ terms are vectors corresponding to the prototype coordinates estimated by the model. The weight w corresponds to the relative strength of the fine-grained memory representation (as opposed to the strength of a prototype in the prior). The larger w is, the closer the memory reconstruction approximates a perfectly unbiased spatial location. $v_{ik}$ captures the relative pull of each of the locations $P_{k}$ for each point i. Finally, c corresponds to a “sensitivity” parameter that models the sharpness of the prototype boundaries.

In the case of simple shapes, we fit the model with only four prototypes using all of the data from the first iteration of our experiment for the fitting process (using the same number of parameters used in ref. 13). Our results are consistent with previous estimates (*SI Appendix*, Fig. S12) (13, 14). However, when fitting natural images, it is hard to estimate the number of modes, and the results are poor predictors of the priors estimated via serial reproduction (*SI Appendix*, Fig. S13). We fit the cam using 5, 10, and 20 prototype locations ($P_{k}$) terms for each of the natural images. We obtained the best estimates for the locations of the $P_{k}$ terms as well as the other parameters of the cam using all initial point locations and the positions in the first iteration for each of the images. We optimized the cam parameters using Matlab’s Optimization Toolbox and the nonlinear programming solver fmincon. The results of this comparison are presented in *SI Appendix*, Fig. S13. Finally, we also completed an analysis comparing the internal reliability of the transmission chain results with the predictions of the cam for one of our images, which shows that using the cam fit to the data in the first iteration of the chains cannot produce estimates of the modes in the prior that are as reliable as those obtained using serial reproduction, even when we equated the amount of data required (*SI Appendix*, Fig. S14).

#### Bayesian model of perceptual biases.

In visuospatial memory, a point location S is encoded into a remembered location T. The neural implementation of these representations in the brain can take many forms (44). However, we are interested only in describing these representations in terms of the distributions that are implied by them (2, 3, 7, 30, 45).

Bayesian models imply that regardless of the sensory encoding process, a reproduction R is based on inferring the original location S from T, following Bayesian inference:

p(R=r|T=t)=p(S=r|T=t)∝p(T=t|S=r)p(S=r). [2]

According to this view, perceptual distortions correspond to systematic (and normative) deviations between R and S, where *R* follows the distribution of the posterior p(S|T).

The degrees of freedom of this approach are 1) the likelihood p(T|S), which describes the noisy observation of the stimulus location; 2) the prior p(S), which describes beliefs that the participant possesses about the distribution of locations given an image; and 3) an assumption about how a reproduced point location is obtained from the posterior distribution. Here, we assume that a reproduction is a sample from the posterior, although other assumptions are possible (18).

#### Bayesian model of serial reproduction and discrimination experiments.

In our serial reproduction experiment, the reconstruction becomes the basis of another iteration, and this process is repeated. We assume that participants use only the current point location as a basis for their perceptual decision (the Markovian assumption) (*Discussion*). Formally, the transmission chain can be described in terms of a sequence of random variables (*SI Appendix*, Fig. S2*A*):

$$\ldots \to S_{t}\to T_{t}\to R_{t}=S_{t+1}\to \ldots ,$$ [3]

where $S_{t}$, $T_{t}$, and $R_{t}$ are the veridical location, sensory encoded representation, and the inferred location at step t, respectively.

Formally, our modeling approach assumes the following.

• Given an image, participants have a shared prior over point locations, represented as a probability distribution over the image: p(S). We assume that locations are encoded with respect to a sensory parsing of the image content. As a result of this process, a prior is generated that reflects the participant’s belief state about probable locations over the image.
• There is a likelihood function p(T|S) that varies in its form between the fixed and efficient encoding models (*SI Appendix* has details). The likelihood carries information about the shape and magnitude of the noise. Regardless of its shape, we assume it is available for sensory inference.
• Participants infer point locations by computing the posterior (Eq. **2**):

$$\begin{array}{cc} p\left(R=r|T=t\right) & =p\left(S=r|T=t\right)\\ & =\frac{p\left(T=t|S=r\right)p\left(S=r\right)}{\int p\left(T=t|S=r^{\prime }\right)p\left(S=r^{\prime }\right)dr^{\prime }} \end{array}.$$ [4]

• A participant’s response (the reproduction from memory of a point location) is a sample from the posterior (refs. 18 and 30 have other choices, such as choosing the mean of the posterior).

From this, we can derive

$$\begin{array}{cc} & p\left(S_{n+1}=r|S_{n}=s\right)=p\left(R_{n}=r|S_{n}=s\right)\;=\\ & \int p\left(R_{n}=r|T_{n}=t\right)p\left(T_{n}=t|S_{n}=s\right)dt \end{array}.$$ [5]

Given an initial distribution $p\left(S_{0}\right)$, the steps of the transmission chain experiment are fully determined by recursively integrating the following (this is demonstrated in Fig. 2*A* and *SI Appendix*, Fig. S2*B*):

$$p\left(S_{n+1}=r\right)=\int p\left(S_{n+1}=r|S_{n}=s\right)p\left(S_{n}=s\right)ds.$$ [6]

This formulation provides an explicit prediction with respect to discrimination accuracy (7). We can write the perceptual sensitivity ($d^{\prime }$) of a discrimination experiment with respect to two point locations $S_{1}$ and $S_{2}$ in the following way:

$$d\left(S_{1},S_{2}\right)=\frac{\tilde{\mu }\left(S_{1}\right)-\tilde{\mu }\left(S_{2}\right)}{\sqrt{\left(\tilde{\sigma }{\left(S_{1}\right)}^{2}+\tilde{\sigma }{\left(S_{2}\right)}^{2}\right)/2}},$$ [7]

where $\tilde{\mu }$ and ${\tilde{\sigma }}^{2}$ are the mean and variance of R, which can be computed from the formula for the posterior.

#### Serial reproduction converges to the prior.

Here, we prove that under the assumptions stated above, the prior p(S) is the stationary distribution of the Markov chain in Eq. **3**. We denote the prior as π(s)=P(S=s). Using Eqs. **2** and **5**, it follows that

$$p\left(S_{n+1}=r|S_{n}=s\right)=\int \frac{p\left(T_{n}=t|S_{n}=r\right)\pi \left(r\right)}{\int p\left(T_{n}=t|S_{n}=r^{\prime }\right)\pi \left(r^{\prime }\right)dr^{\prime }}p\left(T_{n}=t|S_{n}=s\right)dt.$$ [8]

We will now show that π(s) is the stationary distribution of the chain: $\int p\left(S_{n+1}=r|S_{n}=s\right)\pi \left(s\right)ds=\pi \left(r\right)$.

This follows from a direct computation:

$$\begin{array}{cc} & \int p\left(S_{n+1}=r|S_{n}=s\right)\pi \left(s\right)ds\;=\\ & \int \left[\int \frac{p\left(T_{n}=t|S_{n}=r\right)\pi \left(r\right)}{\int p\left(T_{n}=t|S_{n}=r^{\prime }\right)\pi \left(r^{\prime }\right)dr^{\prime }}p\left(T_{n}=t|S_{n}=s\right)dt\right]\pi \left(s\right)ds\;=\\ & \int \frac{p\left(T_{n}=t|S_{n}=r\right)\pi \left(r\right)}{\int p\left(T_{n}=t|S_{n}=r^{\prime }\right)\pi \left(r^{\prime }\right)dr^{\prime }}dt\int p\left(T_{n}=t|S_{n}=s\right)\pi \left(s\right)ds\;=\\ & \int p\left(T_{n}=t|S_{n}=r\right)\pi \left(r\right)dt=\pi \left(r\right) \end{array}.$$ [9]

The first equality holds true by substituting the formula above for $p\left(S_{n+1}|S_{n}\right)$. The second equality is due to a change in the order of integration. The last equality holds true because $\int p\left(T_{n}=t|S_{n}=r\right)dt=1$. Note that in past work (18, 19), S is observed by both the participant and the experimenter, whereas in our case, T is observed by the participant and S is observed by the experimenter. In the former case, the chain converges to a stationary distribution π(t) equal to the prior predictive distribution: π(t)=∫p(T=t|S=s)p(S=s)ds, whereas in our case, it converges to the prior π(s)=p(S=s).

#### Numerical simulations.

We computed simulations of the dynamics of the transmission chain experiments as well as the discrimination experiment results analytically (Figs. 2*D* and 3*A*). We provide two-dimensional (2D) illustrations of the efficient encoding and fixed encoding models in Fig. 2 *D* and *E*, showing the opposing predictions of the two models regarding discrimination accuracy. We assumed that the prior is given as a discrete distribution $p\left(S=x_{i}\right)$ on grid points $x_{i}$. Note that there are N grid points in the one-dimensional (1D) case and $N^{2}$ grid points in two dimensions (N is the number of grid points per dimension). We also assume that the likelihood is given as a matrix $p\left(T=x_{j}|S=x_{i}\right)$. This matrix represents the probability associated with a noisy observation $x_{j}$ originating from a veridical location $x_{i}$. Note that in the 2D case, this matrix will be of size $N^{4}$. We then use Eqs. **4**–**6** computed numerically on the grid points. We also use Eq. **7** for computing the predicted discrimination accuracy ($d^{\prime }$). In the 2D case, we used the following approximation: We projected the 2D distributions to the 1D line connecting the two points. In this way, we can avoid the more complex analysis associated with 2D signal detection theory (46). Additional details about the simulation of the discrimination experiments are provided in *SI Appendix, Discrimination simulations*. Code for the 1D and 2D simulations and $d^{\prime }$ computation is given as part of the open science folder associated with this paper (https://osf.io/cza25/).

## Supplementary Material

Supplementary File

## Data Availability

All data and materials reported here are available on the Open Science Framework (OSF), in the repository named Serial Reproduction Reveals the Geometry of Visuospatial Representations, at https://osf.io/cza25/ (46).
