# Supplementary material for: Serial reproduction reveals the geometry of visuospatial representations
Source: Proc Natl Acad Sci U S A. 2021 Mar 26;118(13):e2012938118. doi: 10.1073/pnas.2012938118 (PMC8020772; doi:10.1073/pnas.2012938118)
Supplement: Supplementary File [file pnas.2012938118.sapp.pdf]

PNAS
www.pnas.org

Supplementary Information for

Serial Reproduction Reveals the Geometry of Visuospatial Representations

Thomas A. Langlois, Nori Jacoby, Jordan W. Suchow, and Thomas L. Griffiths

Thomas L. Griffiths. E-mail: tomg@princeton.edu

This PDF file includes:

Supplementary text
Figs. S1 to S23
References for SI reference citations

Supporting Information Text

Participants

Participants were recruited online using Amazon Mechanical Turk (AMT). The experiments were approved by the Committee for Protection of Human Subjects (CPHS) at the University of California, Berkeley and by Princeton University's Institutional Review Board (IRB) for Human Subjects under protocol #10859 (Computational Cognitive Science). We obtained informed consent from all volunteers. Participants took part in the experiment anonymously, and no demographic information was collected (see (1) for information about AMT workers). For the serial reproduction experiments, compensation was between $1.4 and $1.6, depending on the participant's performance. Typical participation included 105 trials, and the average time needed to complete the task was about 12-14 minutes. Participants could take part only once per experiment; however, they could take part in more than one experiment. A typical experiment included about 100 participants. For the discrimination experiment, compensation was between $0.75 and $1.0, and typically included 160 trials. For the patch rating tasks, participants received $1.5 for participating, and completed 136 trials. Participants could take part in as many discrimination and patch rating experiments as they wished. Fig. S23 presents the exact number of participants in each experiment. The overall number of participants in all experiments was 9202. We only recruited participants who had 95% or more of their completed HITs approved.

Stimuli

Stimuli for all 85 experiments are available in an open science database (link: https://osf.io/cza25/), and Fig. S23 presents a summary of all stimuli used. We used a range of images that included objects, natural scenes, indoor scenes, reachable scenes (2), man-made and natural objects, and faces. We selected images from databases that also contained human semantic segmentations and eye-tracking fixations (3) and in some cases, eye-tracking fixations on a variety of perceptual tasks (3, 4).

Experiments 1-4 (Fig. 1): Serial reproduction experiments with shaded shapes. We used an image of a uniform gray circle, triangle, square, and pentagon, as previous work explored biases using simple geometric shapes (e.g (5)).

Experiments 5-11 (Fig. 1): Serial reproduction experiments using natural images. All the natural images we used were obtained from the PASCAL-S dataset, a subset of the PASCAL VOC 2010 segmentation challenge dataset (3, 6–10). We selected this database as it provided annotations of segmented regions in the images as well as free-fixation eye movement data. We used grayscale versions of these images so that the red point used in the experiments would be clearly visible. We used seven images depicting an airplane, a boat in a harbor, a bird on a branch, a horse, a room, a human face, and a lighthouse.

Experiments 12-16 (Fig. S15): Serial reproduction experiments using semantic segmentations of natural images. The PASCAL-S dataset contains human-generated segmentation maps (11). The segmented regions were rendered to an image with the same dimensions as the original images, with each segmented region shown in a unique shade of gray. We used segmented images that corresponded to the natural grayscale images used in experiments 5-11, although they were not available for the face and lighthouse images.

Experiments 17-27 (Fig. 3, Fig. S7): Visual discrimination experiments. We used the same shape and natural images used in experiments 1-11.

Experiments 28-29 (Fig. S17): Serial reproduction experiments using images with illusory corners. We used the image of the shaded square manipulated with a smooth gradient such that the upper right corner vanished into uniform white. We also used the image of the human face modified such that a gradient erased the right side of the image.

Experiments 30-32 (Fig. S9A-B): Serial reproduction experiments: precision manipulations. We used the airplane image used in Experiments 5-11, as well as two versions of the image with reduced contrast and added Gaussian noise.

Experiments 33-34 (Fig. S9C-D): Serial reproduction experiments: payoff and Markovian assumption manipulations. We used the plane image used in Experiments 5-11.

Experiments 35 (Fig. S9B): Serial reproduction experiments: delay manipulation. We used the plane image used in Experiments 5-11.

Experiments 36 (Fig. S20): Serial reproduction experiments (within-subject design). We used the pentagon shape image used in Experiments 1-4.

Experiments 37 (Fig. S21): Serial reproduction experiments (within-subject design). We used the plane image used in Experiments 5-11.

Experiments 38-45 (Fig. S18, Fig. S19): Serial reproduction experiments: comparisons to fixation maps. We used a subset of 8 images from the database of images used by (4), for which eye-movement fixation maps were available for a free-viewing task, a cued object search task, and a saliency search task.

Experiments 46-61 (Fig. S19): "Graspability" and "meaning" map experiments. We used the same images used in Experiments 38-45.

Experiments 62-68 (Fig. S10): Forward and backward noise masking experiments, blank encoding, and blank reproduction manipulations. We used the lighthouse image from Experiments 5-11, as well as a blank grayscale rectangular image with the same aspect ratio as the lighthouse image.

Experiments 69-72 (Fig. S16): Center of mass (COM) model comparison. We used two grayscale versions of landscape images used in prior work studying spatial memory biases (12–14), as well as grayscale versions of the two images containing only uniform segmented regions corresponding to distinct RGB profiles rather than the original image textures.

Experiments 73 (Fig. S11): Serial reproduction experiments: Temporal encoding manipulation with complex shape images. We used a 19 -sided regular shaded polygon (a uniform gray region with the same inner gray values as the shapes used in experiments 1-4).

Experiments 74-85 (Fig. S11): Serial reproduction experiments: Spatial complexity manipulation with complex regular polygon images. We generated shaded regular polygons with 3, 4, 5, 7, 9, 11, 15, 19, 21, and 25 edges, as well as a shaded circle such that all spanned the same area and were the same uniform gray as the stimuli used in experiments 1-4.

Procedure

Experiments 1-16, 28-45, and 62-85 were programmed using the Dallinger platform for laboratory automation for the behavioral and social sciences (15). The discrimination and patch ratings experiments were programmed as Amazon Mechanical Turk experiments using JavaScript.

Experiments 1-4 (Fig. 1): Serial reproduction experiment with shaded shapes. Participants were presented with an image of a gray shape with a red point initialized somewhere on the image (both inside and outside the shape boundaries) for 1000 ms. The initial locations were sampled from a uniform distribution over the image. Participants were instructed to reproduce the exact location of the point relative to the image. Overall positions of the displays, including the point and image, were shifted by a random horizontal and vertical offset between 0 and 80 pixels on the screen canvas so that participants could not track the absolute positions of the points. The canvas dimensions were 590 by 590 pixels. The response was then sent to another participant who performed the same task. A total of twenty iterations were completed for each chain. We terminated each experiment after approximately 12 hours. As a result, the number of total chains varied between experiments (250-577 chains see Fig. S23). Most results were obtained by aggregating the results of two separate experiments containing about 250 chains each. Typical participation included 105 trials, and the average time needed to complete the task was about 12-14 minutes. A typical experiment included about 100 participants. Fig. S23 presents the number of participants in each experiment. For the serial reproduction experiments, compensation was between \$1.4 and \$1.6, depending on performance. Participants could take part only once per experiment; however, they could take part in more than one experiment. We only retained the chains that were full, and discarded any chains that did not reach twenty iterations.

Experimental trials. Following ten practice trials, there were 95 experimental trials. Only a given shape or image was presented throughout an experiment in both the practice and experimental trials. For each of the 95 experimental trials, the presentation time was 1000 ms. Participants were given trial-by-trial feedback regarding their accuracy. If their responses were within a box around the presented ("objective") location to be remembered that was 8% percent of the width and height subtended by the shape, they received a small monetary bonus and positive feedback (a message in green: "This was accurate"). If not, they received no additional bonus and were presented with negative feedback (a message in red: "this was not accurate"). Incorrect trials were discarded from the experiment, and the corresponding node in the transmission chain was randomly reassigned to another participant; this was done to eliminate the possibility of false responses by bots (16) and discourage inattentive participants. Participants could take part only once within each chain (see Fig. S1).

Practice trials. Practice trials were identical to the experimental trials, except that the margin of error was reduced to a box that was 5% of the width and height subtended by the shape image, and the presentation time was 4000 ms. In these trials, the point location was randomized uniformly within the image.

Experiments 5-16 (Fig. 1, Fig. S17, and Fig. S15): Serial reproduction experiments using natural images, and image segmentation maps. The procedure was identical to Experiments 1-4 except that due to the increased task difficulty, allowable margins of error were 7% for the practice trials and 15% for the experimental trials.

Experiments 17-27 (Fig. 3 and Fig. S7): Visual discrimination experiments. We used the exact same stimulus images that were used in the serial reproduction experiments with the natural images. For the shape images, we used versions that were reduced in size to limit the number of trials required to obtain full d' maps (although we preserved the aspect ratios of the gray shapes in the images). We produced a regular grid of point locations that spanned the full area of each of the images. The grid points were 7 pixels apart. During the task, participants saw an image presented for 1000 ms with a red point placed over it (Fig. 3B). Following a 1000 ms delay with a blank screen, the image reappeared with the point either in the same exact location relative

to the image or in a shifted position (the durations of the display were identical to those in the serial reproduction experiments). In the "shifted" condition, the shifted point was offset by 6 pixels somewhere along a circular radius around the original point location, sampled at random. The second display remained for 1000 ms on the screen and was followed by a 2AFC ("red dot same", or "red dot shifted"). Participants could take as long as they liked to choose a response, although they had to complete the experiment within one hour before the HIT expired. We obtained responses from a total of 20 participants for each grid point, and for each condition ("same" or "shifted"). The full instructions at the start of the experiment were as follows: "In this experiment, you will see two images presented one after the other (the gray triangles below). These images will have a red dot placed over them. Your task is to determine if the red dot is in the same spot relative to the image for both images in the pair, or if the red dot appears displaced the second time it is presented. NOTE: The displays will be displayed at random positions on the screen, even in cases when the red dot is placed in the EXACT SAME spot over the image! So part of the challenge is to ignore the random shifting of the overall display, and focus on the RELATIVE positions of the dots in relation to the images, ignoring the random overall displacements. Finally, in the actual experiment, the image will be a natural black and white photograph instead of the gray triangle in these instructions." For the discrimination experiment, compensation was between \$0.75 and \$1.0, and typically included 160 trials. Participants could take part in as many discrimination experiments as they wished.

Experiments 28-29 (Fig. S17): Serial reproduction experiments using images with illusory corners. The procedure was identical to the one used for Experiments 1-4 for the square with an illusory corner, and identical to the one used for Experiments 5-11 in the case of the face with the illusory eye.

Experiments 30-32 (Fig. S9A-B): Serial reproduction experiments: precision manipulations. We manipulated encoding precision through either stimulus manipulations, or a timing manipulation for one of the natural images (the plane image). For the timing manipulation, we reduced the encoding time during the presentation phase from 1000 ms to 200 ms. For the stimulus manipulations, we kept the original encoding time of 1000 ms during the presentation phase, but we changed the stimulus image by (1) reducing the contrast of the stimulus image, or (2) adding Gaussian noise to the stimulus image (see Fig. S9A-B). Aside from these changes, the transmission chains were identical in design to the one that produced the original finding for the same image (See Fig. S9E).

Experiments 33-34 (Fig. S9C-D): Serial reproduction experiments: context manipulations. We manipulated the context in two ways: (1) by interleaving the experimental trials with trials in which point locations were presented in random locations (to test carry-over effects and the Markovian assumption), and (2) by introducing a payoff in the task. The payoff manipulation examined whether a monetary incentive can alter participant response patterns. We enforced uniformity by adding dummy trials between each of the trials in which a point location sampled from a uniform distribution over the image was presented. In the payoff manipulation, we rewarded correct responses that were within 2.5% of the width and height subtended by the image, and to the right of the true point location with double the normal bonus (correct responses to the left of the true location were only awarded the normal bonus). We provided trial-by-trial feedback indicating that the response was awarded double the normal bonus or just the normal bonus. The instructions at the beginning of the experiment also indicated that correct responses that were to the right of the true location would be awarded double.

Experiments 35 (Fig. S9B): Serial reproduction experiments: delay manipulation. The design was identical to the design used in Experiments 5-11 except that the delay phase was extended from 1000 ms to 2000 ms.

Experiments 36 (Fig. S1B and Fig. S20): Serial reproduction experiments (within-subject design). We used a fully within-subject design, where each participant was assigned a set of chains to complete in full (rather than the between-subject design in which participants only participated in a chain once).

Experiments 37 (Fig. S1B and Fig. S21): Serial reproduction experiments (within-subject design). The design was identical to the within-subject design used for experiment 36.

Experiments 38-45 (Fig. S18, Fig. S19): Serial reproduction experiments: comparisons to fixation maps. The procedure was identical to the one used for Experiments 5-11.

Experiments 46-61 (Fig. S19): "Graspability" and "meaning" map experiments (Fig. S19). We used the procedure described by (17–19) to generate dense "graspability" and "meaning" maps for 8 images in the database of images used by (4) for which detailed eye-movement fixation patterns were available. To do this, we extracted a 20 by 20 grid of fine-scale circular image patches from each of the images, and a 12 by 12 grid of coarse-scale circular image patches from the same images. The patches were extracted from high-resolution versions of the images that were full-color 2430 by 2430 pixel images. The diameter of the fine-scale patches was 256 pixels, and the diameter of the coarse-scale patches was 442 pixels (see Fig. S19A). We presented each of the patches along with a small thumbnail of the full image that included a green circular marker over the image to indicate where the patch was extracted from, for context. Participants either rated the "informativeness or recognizability" of the image content revealed by each of the patches using a Likert scale (1 = "Very low recognizability", 2 = "Low recognizability", 3 = "Somewhat low recognizability", 4 = "Somewhat high recognizability", 5 = "High recognizability", 6 = "Very high recognizability"), or they rated the "graspability" of the image content revealed by each of the patches (also

using a Likert scale, see Fig. S19B). Participants rated a total of 136 random patches from a given image per experiment, and we obtained judgments from 10 unique participants for each image patch over AMT. Participants were paid $1.5 for their participation.

Experiments 62-68 (Fig. S10): Forward and backward noise masking experiments, blank encoding, and blank reproduction manipulations. The procedure was nearly identical to the one used for Experiments 5-11. In the case of the forward and backward masking experiments, the differences were the following: the encoding time was reduced from 1000 ms to 300 ms. In addition, we introduced 500 ms forward and backward noise masking to the encoding phase. The noise sequences were composed of random $1/f$ "pink" noise images that were the same dimensions as the natural and blank images. In one experiment, the lighthouse image was used during the encoding and reproduction phase, in the second, the lighthouse image was shown during the encoding phase, but replaced with a blank uniform gray frame during the reproduction phase. In the case of the blank encoding and blank reproduction manipulations, the encoding time was also manipulated from 1000 ms to 300 ms, but with no noise masking during the encoding phase. In the first experiment, we used the lighthouse image during the encoding phase, followed by the blank uniform gray frame during the reproduction phase. In the second, the order was reversed. Finally, we repeated the experiment using only uniform gray frames as a control experiment (shown for 1000 ms) as well as using the lighthouse image throughout with a 300 ms encoding time.

Experiments 69-72 (Fig. S16): Serial reproduction experiments: landscape images. The procedure was identical to the one used for Experiments 5-11.

Experiments 73 (Fig. S11): Serial reproduction experiments: Temporal encoding manipulation with complex shape images. The procedure was identical to the one used for experiments 1-4, except that the encoding time was changed from 1000 ms to 300 ms).

Experiments 74-85 (Fig. S11): Serial reproduction experiments: Spatial complexity manipulation with complex regular polygon images. The procedure was identical to the one used for experiments 1-4.

Statistical Analysis

The Jensen-Shannon Divergence (JSD). In order to compute the distance between distributions we used the Jensen-Shannon Divergence (JSD). The JSD of two distributions P and Q is defined by the following:

$$JSD(P,Q) = \frac{1}{2}KL\left(P \,||\, M\right) + \frac{1}{2}KL\left(Q \,||\, M\right)$$

where $M = \frac{1}{2}(P+Q)$ and $KL\left(P_1 \| P_2\right)$ is the Kullback-Liebler (KL) divergence:

$$KL\left(P_1 \| P_2\right) = \int_s P_1(s)\log_2 \frac{P_1(s)}{P_2(s)}ds$$

The JSD is symmetric, and bounded between 0 and 1. It is equal to 0 when $P_1 = P_2$.

Between-subject and within-subject serial reproduction designs. Our main findings were obtained using a strictly between-subject design (see Fig. S1A). In this design, AMT participants could only participate in a chain once (each trial corresponded to a node inside a different chain). We also completed two within-subject serial reproduction experiments. In these cases, participants were assigned full chains, and completed all the iterations for their assigned chains (see Fig. S1B). We show the results for all iterations of the chains for both designs, and for both a shape and a natural image in Fig. S20A-B, and Fig. S21A-B. The within-participant design requires that each participant complete entire chains, so for an experiment with 100 trials, that limits the number of chains to only 5. This means that each participant reconstructs point locations shown in a very restricted part of the space. The task then becomes obviously biased in a few spatial locations which may appear repetitive to a participant, since the same 5 points repeat in approximately the same places. However, when a participant completes trials in a fully between-subject design he/she participates only once per chain and is allowed to participate in 100 different chains initialized in 100 unique random locations in the image (for 100 trials). This makes the task more engaging and potentially explains the reduced noise in this case.

Encoding precision manipulations, payoff, and other manipulations. We completed direct manipulations of encoding precision using the serial reproduction paradigm for one of our natural images (airplane image). We also completed a payoff manipulation and a uniformity manipulation to test the Markovian assumption.

Encoding precision manipulations. We manipulated encoding precision in two ways: by reducing the encoding time during the presentation of the point location from 1000 ms to 200 ms, or by (1) reducing the contrast of the stimulus image, or (2) adding Gaussian noise to the stimulus image (see Fig. S9A-B). We found that these manipulations had a significant effect on the structure of the resulting priors (Fig. S9F-G), which appear simplified relative to the original finding using the same image (Fig. S9F). We confirmed this quantitatively using the Jensen-Shannon-Divergence (JSD), and the following analysis: We started by obtaining 1000 split-half random sample pairs of the data from the original experiment (1000 unique partitions of

the chains). This yielded 1000 data pairs of approximately 250 unique chains (two equal sized random partitions of the full 500 chains). We then fit a KDE to the data in the 20th and final iteration of each of the two partitions for all 1000 pairs, yielding 1000 KDE pairs. We then computed the JSD between each of the pairs, which yielded 1000 JSD values. This distribution provides a measure of the internal consistency and variation of the KDEs of the original data. In order to test if the encoding precision manipulations had an effect on the structure of the priors, we repeated the same procedure described above, except that instead of comparing KDEs fit to random splits of the original data, we compared KDEs fit to random partitions of the original data to KDEs fit to random partitions of the data obtained from the manipulations. This yielded a distribution of 1000 JSD values for each of the experimental manipulations, each providing a measure of how much the manipulations produced distributions that diverged from the original distribution of point locations in the 20th iteration of the chains. We then obtained 1000 JSD differences by subtracting each of the 1000 JSD values for each of the manipulations from the 1000 JSD values obtained from split-half samples of the original data. Finally, we tested whether each of these distributions of differences were significantly different from 0 (see Fig. S9G). We found that JSD differences for the 200 ms encoding time, contrast, and Gaussian noise stimulus manipulations were significantly different from 0 in all cases ($p < 0.001$). We applied the Bonferroni correction to adjust for multiple comparisons.

Payoff and other manipulations. We tested the effect of introducing a payoff manipulation Fig. S9C-D. In addition we tested the Markovian assumption by interleaving experimental trials with trials that have a uniformity prior on point locations throughout the chains. This way, if contextual information from previous trials is important we expected to measure a change in the structure of the prior. We found that neither manipulation produced significant changes in the structure of the priors when compared to the original findings. We evaluated this quantitatively using the JSD measure in the same way that we evaluated the effect of the encoding precision manipulations (see section above for details).

Testing Deviation from a Uniform Distribution Using JSD Distance (Fig. S5). To quantitatively test whether the distribution of seeds as well as the subsequent iterations deviated significantly from a uniform distribution over the image, we computed the mean JSD distance between the parametric KDE from all experimental points of a given iteration and KDEs of points sampled from a uniform distribution over the image. To evaluate statistical significance, we created two randomized data sets where the same number of points as in the experimental data were sampled from a uniform distribution. We then computed the JSD between the KDEs of these two data sets. This was necessary because the JSD between two distributions is always non-negative, and therefore any distribution evaluated from a finite number of points would have a non-zero distance from a uniform distribution. As expected, the JSD distance between the initial seeds and uniform samples was not significantly different from the null distribution. The data for the first iteration deviated significantly from a uniform distribution for the triangle, square and pentagon ($p = 0.035$, 0.001, 0.004), but not significantly for the circle ($p = 0.09$). However, for all subsequent iterations (iterations 2-20) the distributions significantly differed from uniform ($p < 0.001$ for all shapes and iterations. We applied the Bonferroni corrections for multiple comparisons. Similarly, the JSD distances between the initial seeds of natural images (Experiments 5-11) were not significantly different from the null distribution ($p > 0.13$ for the face, lighthouse, bird, room, plane, horse, and boat images); marginally significant for iterations 1-4 ($p = 0.001$-0.3 for the 7 images); and highly significant for iterations 5-20 ($p < 0.001$ for all iterations and images, Bonferroni correction applied). Fig. S6 shows the results for natural images, and shapes (Experiments 1-11). Fig. S20A and S21A show the distributions of points for all 20 iterations for the pentagon shape, and a natural image, respectively.

Transmission Chain Convergence Analysis (Fig. S5 and S6). To assess whether the transmission chain process converges within 20 iterations, we used three methods: distributional distance between each iteration and the last iteration, distributional distance between adjacent iterations, and copying accuracy.

JSD distance between each iteration and the last iteration. This method is used to assess whether the last iteration is characteristic of a converged state. If the chain converged we expect the distance to the last iteration to decrease and stabilize as the iteration number approaches the final iteration (the distance between iteration 1 and 20 should be larger than the distance between iteration 5 and 20, and so on). For each of the 20 iterations and initial seeds we computed the parametric KDE as explained in the Methods section. We then computed the JSD distance between each iteration and the last iteration. In other words, we tested the difference between JSD distances of pairs of iterations (For example, we compared the distance between the two last iterations (19 and 20) with the distance between iteration K and 20 (K = 0 is the initial seeds). We then tested whether these differences were significant, and when they ceased to be significant). To test for statistical significance, we randomized 1000 datasets by sampling the data for all iterations with replacement and computing the distance between the KDEs fit to the bootstrapped data from different iterations. Bonferroni corrections were applied in all cases.

The JSD distance between iteration 1 and 20 was significantly larger than the one between iteration 19 and 20 for all experiments ($p < 0.001$ for the shapes and the natural images). However, the distance decreases with a monotonous trend (see Fig. S5). The distance between iteration m and 20 was not significantly different from the distance between iteration 19 and 20 for all $m \geq K$ where K was 18, 16, 15, 18 for the circle, triangle, square and pentagon; and $K = 12$, 12, 13, 11, 10, 11, 13 for the face, lighthouse, bird, room, plane, horse and boat images).

JSD distance between subsequent iterations. In this method, we compared the distance between pairs of subsequent iterations (comparing the distance between iterations 1 and 2 to the distance between iterations 2 and 3, and so on). At a converged state of the process we expect that the distance between subsequent iterations will not significantly change. We found that

for geometric shapes, the distance between iteration 0 and 1 was significantly different from the distance between iteration 19 and 20 for the triangle and pentagon ($p < 0.001$, $p = 0.001$) but not significant for the circle and square ($p = 0.371$, $p = 0.121$), corrected for multiple comparisons). The distance between iteration m and $m{+}1$ was not significantly different from the distance between iteration 19 and 20 for all $m > K$ where K was larger than 1 for all the shapes. Note that the results for the natural images did not show a significant decrease in this metric for all iterations, indicating a gradual change with fixed temporal dynamics. The results of this analysis are shown in Fig. S5.

Copying accuracy. In this method we computed the copying accuracy using the root of the mean squared Euclidean distances between stimulus and response vectors. To test for significance, we randomized 1000 datasets by sampling with replacement from the experimental data and computing the copying accuracy for each iteration. The results of this analysis are also shown in Fig. S6. We found that for geometric shapes, copying accuracy of the first two iterations was significantly different from the copying accuracy for the last iterations, for the circle, square, triangle, and pentagon ($p = 0.044$, $p < 0.001$, $p < 0.001$, $p < 0.001$). The copying accuracy of iteration m and $m{+}1$ was not significantly different from the copying accuracy of the last iterations for all $m > K$ where K was 5, 15, 15, 9 for the circle, square, triangle, and pentagon respectively). Note that as the results of the analysis of JSD distances between subsequent iterations show, the copying accuracy for natural images did not reveal a significant decrease, suggesting that the average step size of the process stabilized right from the beginning.
In a regime where there are numerous nearby landmarks in the scene (as with natural images), copying accuracy does not necessarily decrease with additional iterations, as the bias is approximately related to the distance between the current point and the nearby landmark. In the case of shape images however, where there are only a few landmarks (vertices that can be far from a point location to be remembered) the analysis shows a systematic decrease in the copying accuracy for the first few iterations.

Temporal Encoding Manipulation (Fig. S11). We predicted that using shorter exposure times would force participants to use more compact internal representations. When participants were presented with a 19-sided regular polygon shape for 300 ms, their shared internal representation tended towards a pattern of biases that was more similar to the results for the circle when compared to the results obtained for the 19-sided polygon using a 1000 ms presentation duration. We obtained 1000 bootstrapped samples of the final iteration results with replacement for both manipulations and compared the KDEs fit to these samples with KDEs fit to 1000 bootstrapped samples of the final iteration of the circle result. We found that the JSDs of the results for the 300 ms encoding time manipulation (average JSD =0.1647 , SD = 0.011) were smaller than the JSDs between the results for the 1000 ms manipulation (average JSD = 0.2336, SD = 0.012, $p < 0.001$), indicating that reduced encoding time for a complex polygon does indeed result in a simpler pattern of biases that is closer to the pattern for the circle (Fig. S11). This finding is consistent with a theoretical prediction of the efficient encoding theory, namely that constraints on encoding resources will result in simplified internal representations rather than simply noisier versions of the representation obtained without reductions in encoding time.

Complexity Manipulation (Fig. S11). The apparent increase in peaks in visual memory KDEs for more complex regular polygons led us to consider changes to the internal representation in the limit, as the regular polygons become more complex and start to approximate a circle. Using the same bootstrapping procedure used for quantifying the differences in JSD for the temporal encoding manipulations, we compared the JSDs obtained from comparing the final results for each of the regular polygons to the final results for a circle of the same area as the polygons. We found that the JSDs obtained for the 21-sided and the 25-sided polygons (average JSD = 0.137, SD = 0.009, and average JSD = 0.075, SD = 0.007) were significantly different from each other ($p < 0.001$), as were the JSDs obtained for the 15-sided and 17-sided polygons (average JSD = 0.275, SD = 0.013, average JSD = 0.205, SD = 0.011, respectively, $p < 0.001$), and the JSDs obtained for the triangle and diamond shape (average JSD = 0.624, SD = 0.009, average JSD = 0.672, SD = 0.004, $p < 0.001$). Overall, the pattern shows a near-monotonic decrease in the JSD means as the shape complexity increases, indicating that as polygons acquire more edges, the resulting memory biases begin to resemble those obtained for a circle. This finding is also in line with predictions of the efficient encoding model, which theorizes that limits in encoding resources will result in simplified internal representations rather than just noisier ones.

CAM bootstrapping reliability analysis (see Fig S14). We compared the internal reliability of the transmission chain results with the predictions of the CAM for one of our images (the plane image). To do so, we used a variant of bootstrapping. This variant aims to (a) simulate different amounts of chains: this is done by bootstrapping with replacement, and (b) avoid overfitting in each of the methods: this is done by separating the data into training and testing split-half datasets. In the procedure, we start by performing a random split of the data, keeping one split-half as the testing dataset and the other split-half as the training dataset from which we sample with replacement and fit the CAM. The exact analysis is described below:
To compare the internal reliability of the chain results to the CAM estimates, we did the following 1) we computed the correlation between KDEs fit to the data in the last iteration of two random partitions (splits) of the data (KDE split-half reliability). 2) We then compared that correlation to the correlation between a KDE fit to the data in split 1 to a CAM estimate fit to the data in split 2. We varied the number of K chains sampled from the chains in split 2 to fit the CAM from the stimulus and response pairs in iteration 1 of the chains. We also used the same K chains in split 2 to fit a KDE when computing the KDE split-half reliability estimates. For each value of K, we computed the correlation to the KDE fit to the data in the last iteration of the 250 chains in split 1 to the CAM and KDE estimates obtained from the K chains in split 2. Finally, we repeated the analysis for each value of K 100 times by obtaining 100 random partitions (splits) of the chains. This procedure is illustrated

in Fig. S14A. It shows, for a given random partition of the data into two equal splits, the model fitting and comparison that we completed to compare the internal reliability of the serial reproduction chain estimates to estimates made using the CAM. Fig. S14B shows the results comparing the internal reliability of the KDEs fit to random splits of the data, as well as the reliability of the CAM estimates, using 5, 10, and 20 prototypes, for each value of K samples from the 2nd partition of 250 chains. The shaded error bars correspond to 100 random partitions of the full chains into two equal parts. The x-axis in the graph (Fig. S14B) is normalized according to the number of participant trials used for the estimation (equating for the fact that the serial reproduction estimates are made from multiple iterations, and therefore more data). This analysis shows that the serial reproduction results are significantly more internally reliable than the CAM estimates for all values of K, even when K is large. In other words, even when the two methods are equated for the amount of data used, the serial reproduction results produce more reliable estimates. This indicates that using the CAM fit to the data in the first iteration of the chains cannot produce estimates of the modes in the prior that are as reliable as those obtained using serial reproduction.

Calculating d'. d' scores were computed for each image, and for each condition ("same" or "shifted" condition in the discrimination task) by calculating the False Alarm (FA) rate (the number of times a given label was selected when the image shown was not an instance of that label, over the number of times that the presented images were not instances of that label), and the HIT rate (the number of times that a given label was selected when the image shown was an instance of that label, over the number of times that all the presented images were instances of that label). d' is given by: $d' = Z(\mathrm{HIT}) - Z(\mathrm{FA})$ where the function $Z(p), p \in [0, 1]$, is the inverse of the cumulative distribution function of the Gaussian distribution.

Model Comparisons: Predicting the Spatial Memory KDE using Local Image Features (Fig. S17, Fig. S15). We extracted local gradient-based corner and edge features using the Canny edge detector, and the Harris corner detector (20–22). We used all allowable parameter ranges and the OpenCV implementation (23) as explained in detail below. We then computed the correlations between the feature maps and the final spatial memory KDEs obtained for the natural images. For each feature detector, we performed a detailed grid search of all the parameter settings within the ranges that are specified for these algorithms. In addition, we added a smoothing parameter (the standard deviation of an isotropic Gaussian kernel that was convolved with the final feature map). We searched for the maximally predictive parameters for a given feature detector as measured by its peak correlation to the concatenated KDEs. The reported result was the one that provided the best correlation among all searched parameters including the smoothing parameter. We selected the parameters that were optimal for predicting the final spatial memory KDEs for all images represented as a single concatenated matrix of each of the individual KDEs, using the corresponding concatenated feature maps. For the other features (centers of mass (CoM), fixations, segmentation image KDEs, discrimination d' maps), there were no parameters aside from the smoothing parameter, which was determined based on which provided the best correlation to the concatenated matrix of the final spatial memory KDEs for the images for which fixations and segmentation maps were available. We then obtained the predictions for each of the individual KDEs using the optimal parameter settings obtained for the concatenated matrix of all KDEs. When testing whether feature maps produced significantly different predictions (correlations to the prior KDEs across all the images), we compared the correlations of the concatenated feature maps to the concatenated image KDEs, and used 1000 bootstrapped samples of the data in the final iteration of the chains to estimate the standard deviation for each of the feature predictions. In addition to showing the results for the individual images, we report significant differences between the predictors across all images where applicable in the main text.

Edges. For each image, we extracted 2,400 Canny edge maps, each corresponding to the feature map for a unique set of parameter settings (20). The Canny edge detector has four parameters: the first and second threshold for the hysteresis procedure, the aperture size of the Sobel filter (which computes the gradient in the image), and the norm of the gradient magnitude (either an L2 norm or an L1 norm). Increments of 10 within a range of 0 to 190 were used for the first and second parameters, three aperture sizes (3, 5 and 7 pixels) were used for the third parameter, and a Boolean setting indicating which norm to use for computing the magnitude was used for the fourth parameter.

Corners. We obtained 280 feature maps for the Harris corner detector (21). As with the Canny edge maps, each corresponded to a unique set of parameter settings from a grid search for its three parameters: the size of the neighborhood considered for corner detection, called the "block size", the aperture size for the Sobel derivative operator, or "k-size", and a free parameter used in the Harris detector equations. For our evaluation, we varied the block size between 2 and 9 pixels, the Sobel filter aperture size between 1 and 7 pixels, and the final free parameter between 0.01 and 0.13 at increments of 0.02 (The appropriate range for this parameter setting specified in the documentation for the OpenCV implementation (23)).

Segmentation maps. A subset of the natural images (all except the face and lighthouse images) which were obtained from the PASCAL-S images came with segmentation maps (11). We used these images and obtained KDEs using our serial reproduction task using the maps instead of the original images, and compared the results to our original KDEs obtained from using the original grayscale natural images.

Centers of mass. We computed centers of mass of the segmented regions by averaging the coordinates of the pixels contained in each segmented region.

Fixations. Fixation data for the small set of images used were obtained from the PASCAL-S dataset. As explained in (10), for each image, 8 subjects performed a "free-viewing" task for 2 seconds. The eye gaze data was recorded using an Eyelink 1000 eye-tracker, at a sampling rate of 125Hz.

Disattenuated correlations. For the correlations presented in Fig. S17, we computed disattenuated correlations to account for measurement error in the KDEs and discrimination accuracy maps. Given samples X and Y of two random variables X' and Y' with correlation r_{xy} and a known (internal) reliability measure for each (r_{xx} and r_{yy}), the estimated disattenuated correlation between X' and Y' is given by $r_{x'y'} = \frac{r_{xy}}{\sqrt{r_{xx}r_{yy}}}$. For the prior estimate reliability, we computed r_{xx} as the mean correlation of 100 pairs of KDEs fit to 100 random partitions of the data in the last iteration of the chains) for each of the images. For the discrimination maps, we computed r_{yy} as the mean correlation of 100 pairs of maps generated from 100 random partitions of the data for each of the images. As for the features that were generated from deterministic algorithms, with no measurement noise (harris corners, and canny edges), the reliability measure r_{yy} was set to 1.

"Meaning" and "Graspability" image patch model comparison (Fig. S19). We used the procedure described by (18, 19). For each of the tasks ("meaning" and "graspability"), we started by averaging the 10 participant responses for each of the patches, and across each of the patch scales (fine and coarse grid scales). Next, we applied a smoothing factor (using Matlab's *imgaussfilt* function to each of the patches. We selected the smoothing factor that maximized the correlation of the maps to the KDEs we obtained for the same images. In addition, we simulated the center bias in overt attention by down-weighting the edges in the maps (using a Gaussian kernel with a fixed standard deviation centered in the image). This procedure is illustrated in Fig. S19C, and examples are shown in Fig. S19D and E.). Finally we computed disattenuated correlation matrices containing all pairwise correlations of the meaning and graspability maps, free fixation, object search, saliency search eye-movement maps, and spatial memory KDEs. For the disattenuated correlations, we used reliability estimates obtained for each of the maps by averaging the correlations between 100 random splits of the data (split-half reliability measures). Example results are shown in Fig. S19E and G. Fig. S19I shows the average over all 8 images. The results replicate the findings by (18, 19), revealing that meaning maps with the center bias were predictive of the attention maps. However, we show that none of the maps (with or without the center bias) were strongly predictive of the spatial memory KDEs, nor were the attention maps with optimal smoothing applied to find the maximal correlation of each map to the KDEs.

Discrimination Map Estimation from Grid 2AFC Responses (Fig. 3 and S7). We created a regular 2D grid of point locations over each image (natural images in Experiments 5-11, as well as the shaded shapes from Experiments 1 and 4). The points were separated by 7 pixels in both the horizontal and vertical dimensions. For each point, we generated trial pairs: "same" and "shifted" conditions where a point was either presented twice in the same location over the image, or it was shifted in the second presentation somewhere over the circumference of a circle of radius 6 pixels centered on the original point location. Each experiment contained 160 trial pairs chosen at random from the full grid of trial pairs. We obtained d' values for each of the discrimination grid points by using the 2AFC responses obtained for each as explained above (see section on calculating d'). We then convolved the grid of raw d' values with a Gaussian kernel to maximize the correlation to the corresponding KDE values at the same point locations. We imputed missing values as the average of the four nearest neighbor values on the grid. Next, we generated full d' map estimates by interpolating the missing values between the grid points using cubic interpolation. Fig. S7 shows the results including the raw d' grid point values, the smoothed d' grid point values before the interpolation, and the smoothed d' interpolated maps (discrimination accuracy maps) for two natural images. Also shown are the smooth d' maps obtained for the shape images.

Models

In our serial reproduction experiment, the reconstruction becomes the basis of another iteration and this process is repeated. We assume that participants use only the current point location as a basis for their perceptual decision (the *Markovian assumption*, see Discussion). Formally, the transmission chain can be described in terms of a sequence of random variables:

$$\ldots \rightarrow S_t \rightarrow T_t \rightarrow R_t = S_{t+1} \rightarrow \ldots \quad [1]$$

where S_t, T_t and R_t are the veridical location, sensory encoded representation, and the inferred location at step t, respectively (see Fig. S2, and Fig. S4). The inferred location in our model is assumed to be sampled from the posterior $P(S|T)$, which depends on both the prior and the likelihood: $P(S|T) \propto P(T|S)P(S)$, as schematically illustrated in Fig. S2 and Fig. S4. Fig. 2A shows the combined effect of encoding $P(T|S)$ and decoding $P(R|T)$. Near a mode in the prior, the posterior becomes distorted, and its mode is shifted towards the mode of the prior. The net effect of both encoding and decoding produces a bias (this is shown in Fig. 2A and C). Far from a mode in the prior, the posterior becomes less distorted, and as a result the reproduction bias is smaller (Fig. 2A and C). Without any further assumptions, one can show that the chain approximates a Gibbs sampler on the joint distribution of T and S, and that it converges to a sample from the prior $p(S)$ (see methods for a direct proof and discussion of a similar but not identical model (24)). This is significant, because it means that distributions of visuospatial memory priors can be approximated directly by iterating the task.

However, Bayesian perception leaves open the question of the exact form of the likelihood and the prior. In the case of fixed encoding, in its simplest form the likelihood is additive, constant over the image, and Gaussian:

$$p(T = t|S = s) = G(t, s, \sigma^2 \cdot I), \quad [2]$$

where σ is the noise and I is the identity matrix, and $G(x, \mu, \Sigma)$ is the Gaussian probability density evaluated at point x with mean μ and covariance matrix Σ. This implies that the noise's covariance structure is the same regardless of location (Fig. S4D). The net effect of encoding and decoding (the reconstructed point) results in a small bias (see Fig. 2A). Note that the only degree of freedom of the model is the magnitude of the noise σ since we assume that the prior is given and can be estimated from the data of the final iteration of the transmission chains.

Having a variable prior and a fixed likelihood implies that discrimination ability is reduced near the modes of the prior and increased between the modes. This "perceptual magnet effect" is due to the contraction of the inferred locations towards the modes of the prior (Fig. 2A). This effect can be evaluated independently of the serial reproduction results through experiments of discrimination accuracy using the same stimuli, where the effect predicts a negative correlation between prior density in the serial reproduction experiment and discrimination accuracy in the discrimination experiment.

An alternative to the fixed encoding model, is a variable precision model where precision varies over the image. A simple non-Bayesian version of this model assumes that a reproduction is a symmetric Gaussian variable with precision $1/\sigma(s)$ that varies from location to location in the image:

$$p(R = r|S = s) = G(r, s, \sigma(s)^2 \cdot I), \quad [3]$$

This model captures variable precision over the image because of changes in $\sigma(s)$, which predicts increased discrimination accuracy in the "absorbing states," which are the regions in the image where $\sigma(s)$ is smallest. As such, the symmetric variable precision model is a non-Bayesian model (there is no inference step), and the reproduction distribution is explicitly specified a priori. This model explains the transmission chain results as a random walk with absorbing states near the landmarks, where $\sigma(s)$ is smallest. This model has predictions that deviate from the Bayesian models with respect to single-trial biases: It does not predict that responses will tend to be oriented towards the nearest landmark. However, we discuss evidence indicating that single-trial biases are clearly present in the data (Fig. S3). This calls for a model that can produce both variable precision and single-trial biases.

One option is to extend the non-Bayesian model by specifying an additional variable bias function $b(s)$ for each point in the image:

$$p(R = r|S = s) = G(r, s + b(s), \sigma(s)^2 \cdot I), \quad [4]$$

However this model requires specifying both $b(s)$ and $\sigma(s)$, increasing the number of parameters of the model significantly, as both these functions need to be defined for all locations in the image. In addition, this model is not comparable with the fixed-precision model described above and often used in previous Bayesian accounts of spatial memory biases (25).

However, there is a relatively recent Bayesian formulation of variable precision that captures "anti-Bayesian" phenomena (26, 27), namely improved discrimination near the modes of the prior. This formulation predicts both non-zero single-trial biases and variable precision without adding any additional degrees of freedom to the model. This model was described for the one-dimensional case in (26, 27) and extended in the present work to higher-dimensional cases. The model predictions are constrained because the model makes a strong assumption regarding the relation between the likelihood and the prior.

According to this model, perceptual biases emerge because of variations in internal noise (precision) due to a non-isotropic likelihood function (Fig. S4C). Moreover, this model provides an alternative account of the prior's origin. A prior distribution over locations in an image may result from selective allocation of coding resources to different visual regions during encoding, a process that produces a transformed internal representation. We assume that an initial sensory parsing of an image is used to determine a coordinate transformation F, which maps Euclidean distances to an internal coordinate system (psychological space) in just-noticeable difference (JND) units (see Fig. 2C and Fig. S4A-C). A perceived point location in this psychological space $F(T)$ becomes the following in the external coordinate system:

$$T = F^{-1}(F(S) + n), \quad [5]$$

where $F(S)$ is the deterministic function and $n \sim N(0, \sigma^2 \cdot I)$.

The transformation F can be interpreted as one that efficiently maps Euclidean distance units into Just-Noticeable-Difference (JND) distance units, and the inverse F^{-1} transforms the internal representation back into a Euclidean coordinate representation (see Fig. 2C).

Fig. 2C illustrates why the "uniform" internal space (in JND units) produces a shift toward the (landmark). In internal space, the posterior is symmetric and Gaussian. However, when it is projected to external space it becomes biased towards the mode because the higher density region accumulates mass closer to the landmark. As a result, the averaged reproduction is shifted toward the landmark (left side of Fig. 2C). Far away from the mode (right side of Fig. 2C), the distortion is much less pronounced and the bias is smaller.

This geometry also explains why discrimination is higher near the landmarks (modes): because pairs of points are perceived to be farther apart in internal units when they are near a mode, as opposed to far from a mode (even when the pairs of points are the exact same distance apart in Euclidean distance units), they are also easier to discriminate given some perceptual noise magnitude σ. This is illustrated in Fig. 2C. More specifically, near a landmark the posteriors are narrow in external units (with only a small bias toward the mode). The net result is increased discriminability (despite a small bias towards the mode).

Far away from the mode, the distortion is less pronounced and the posterior densities are more dispersed, resulting in reduced discriminability. Note that of the two competing effects—the bias, and the reduction in variance, the reduction in the variance is the dominant effect, which is why discrimination increases. This prediction is not obvious a priori, and comes from the specific mathematics described here. In the case of fixed encoding, there is no variance shrinkage and therefore the net effect is a reduction in discrimination accuracy, since point locations with equal variance that are perceived to be closer together will be harder to tell apart.

The transformation F can also be interpreted as one that efficiently maps the external coordinate system (Euclidean space Fig. S4B) into an internal coordinate system (psychological space Fig. S4B) in just-noticeable difference (JND) units. (This "internal geometry" is illustrated in Fig. S4A-B). In the context of our task, the intuition is as follows: Efficient variations in internal noise encode some visual regions (visuospatial anchors) with higher resolution, resulting in a dilation of these regions. In internal units, where regular intervals correspond to units of just-noticeable difference, perceptual noise is isotropic and Gaussian. However, because of the inverse F^{-1}, the transformation of the isotropic perceptual noise into physical space results in a non-isotropic likelihood (see Fig. S4B). This idea is similar to how variations in perceptual sensitivity are reflected in neural representations such as the somatosensory homunculus (28) or retinotopic map (29), where increased resolution is imparted to physical extremities or areas in the visual field that are over-represented by the brain. Note that given the prior, the only degree of freedom of this model is the variance of the noise σ, because the transformation F can be uniquely determined from the prior, and vice-versa. In addition, the transformation F determines the likelihood function $p(T|S)$ (via equation 5), and the posterior $p(S|T)$ (see methods for formula for the posterior). From this we can compute the reproduction $P(R|S)$ which is displayed in Fig. 2C.

To summarize, both models assume an initial step that processes the image content. In the case of the fixed-encoding model, this step generates a belief state of the point locations (prior). This prior is then used during perceptual inference. In the case of the efficient-encoding model, the initial processing step is used to produce an internal coordinate transformation during encoding. This coordinate transformation then determines perceptual inference during decoding.

Notably, the fixed-encoding model predicts a negative correlation between the prior and discrimination sensitivity measured in a discrimination task (Fig. 2D), and (26, 27). In contrast, the efficient-encoding model predicts a positive correlation in the very same experiments (Fig. 2E). We evaluated the models by fitting both to the results of the serial reproduction experiments and testing their discrimination accuracy predictions. We found that the efficient-encoding model predicts detailed discrimination accuracy maps, and higher accuracy in the modes (See Fig. 3).

Fixed-encoding model. Given a prior, the fixed-encoding model has one degree of freedom σ, which corresponds to the noise (in (26) this refers to low-level sensory noise). In this case, the likelihood is $p(T=t|S=s) = G(t, s, \sigma^2 \cdot I)$, where I is the identity matrix and $G(x, \mu, \Sigma)$ is the pdf at point x of a Gaussian distribution with mean μ and covariance matrix Σ. Given the likelihood and the prior and the noise magnitude the dynamics of the model are fully determined from the equations in methods subsection "Bayesian model of serial reproduction and discrimination experiments" and can be computed numerically as explained below.

Efficient-encoding model. We assume that the prior and likelihood originate from a coordinate change given by: $T = F^{-1}(F(S)+n)$, where F is a deterministic function mapping Euclidean (veridical) coordinates into an internal coordinate system in which the prior is uniform, the likelihood is symmetric and Gaussian, and $n \sim N(0, \sigma^2 \cdot I)$. In our case we are given the prior from the transmission chain $p(S)$ and we would like to compute the transformation F and the likelihood $P(T|S)$. In the transformed space, the likelihood is symmetric, isotropic and Gaussian. In the external coordinate system given by F^{-1} (see Fig. S4B) the likelihood is given by:

$$\begin{aligned} p(T=t|S=s) &= p(F^{-1}(F(S)+n)=t|S=s) \\ &= G(F(t), F(s), \sigma^2 \cdot I) \end{aligned} \qquad [6]$$

In the one dimensional case, F is the cumulative distribution associated with the prior $p(S)$ (as computed in (26)): $F(x) = \int_{\infty}^{x} p(S=s)ds$. The prior can be computed from the transformation by taking the derivative.

In this paper, we generalize this approach to the two-dimensional case. For simplicity, we assume that the prior is a probability density with a compact support in $\mathbb{R}^2$. We assume that F is a mapping from Euclidean space to a Riemannian manifold $F: \mathbb{R}^2 \to M$ where areas and distances on the manifold dilate and contract such that the prior becomes uniform (Fig. S4A-B; (30)). In this case, we can compute F from the prior (and vice versa) from noticing that the probability associated with a differential area is invariant under coordinate transformations: $p(S'=s')ds'_1ds'_2 = p(S=s)ds_1ds_2$, where $S' = F(S)$. Consequently, if we choose:

$$ds'_i = \sqrt{P(S=s)}ds_i \qquad [7]$$

we obtain a uniform distribution in the latent space.

We model noise in the transformed space by computing a Gaussian distribution in the transformed coordinate system by defining the following probability density function (30)

$$p(s_1, s_2) = k \cdot \exp\left(-\frac{dist(s_1, s_2)^2}{2\sigma^2}\right), \qquad [8]$$

where $dist(s_1, s_2)$ is the geodesic distance between s_2 and s_1 on M, k is a normalization constant, and σ is the standard deviation of the noise. σ is the only degree of freedom of the model (given the prior). The inverse transformation of this isotropic distribution by F^{-1} is typically asymmetric and non-isotropic (Fig. S4B), and its local variability is inversely proportional to the prior density. In other words, the precision is higher in high-density regions under the prior.

Numerical computation of the likelihood function of the efficient-encoding model. We compute the likelihood $p(T|S)$ expressed in the discrete set $(\{x_i\}_{i=1,\ldots,N^d})$ of N^d points (where d is the dimension). In the one-dimensional case, F is the cumulative distribution of $p(S)$ and can be expressed as $F(x_i) = \Sigma_{j \leq i} p(S = x_j)$. From equation 6 we can compute the likelihood: $p(T = x_i|S = x_j) = G(F(x_i), F(x_j), \sigma^2 \cdot I)$. In the two-dimensional case, we define a regular grid on $\mathbb{R}^2$. We use a fixed resolution within a finite domain that includes the support of $p(S)$. Using a technique similar to Isomap (31), we compute a graph in which each grid point $s_{i,j}$ is associated with the i, j location within the regular grid. We define local distances on the graph $d(s_{i,j}, s_{i+1,j}) = d(s_{i,j}, s_{i,j+1}) = \sqrt{p(S = s_{i,j})}$. This provides a discrete estimate to Equation 7 . We then estimated the geodesic distance $dist(s_{i,j}, s_{k,l})$ as the shortest path on the grid using the Floyd-Warshall algorithm (31). After all pairwise distances were computed, we computed the probability density implied by equation 8 numerically, noting that the unit area $dA(s_{i,j})$ was dilated by exactly a factor of $p(s_{i,j})$. This results in the following explicit formula for the likelihood of the model:

$$p(F(S = s_{i,j})|F(S = s_{k,l})) = k \cdot \exp\left(-\frac{dist(s_{i,j}, s_{k,l})^2}{2\sigma^2}\right) \cdot p(s_{i,j}) \quad [9]$$

where k is computed to satisfy the normalization constraint. This computation fully specified the likelihood function. We then compute the posterior by Bayesian inference. Because this model significantly contracts areas with very small density under the prior, this could cause numerical instabilities. Therefore, we modified the prior used for the numerical computation slightly. Instead of the prior measured from the transmission chain experiment ($P(S)$), we used $p'(S)$, given by $p'(S) = c \cdot max(\epsilon, p(S))$, where c is a normalization constant. In our simulations, ϵ was determined to be 1/100 of the maximal value of a uniform density function over the domain of $p(S)$, c was determined by numerical integration. Note that $p'(S)$ and $P(S)$ only deviate slightly in low probability areas of the space.

Efficient coding and previous work. Wei and Stocker (26, 32) derive the formula for the transformation F based on principles of efficient coding. According to their approach, the sensory encoding T of the stimulus S is determined by a trade-off between preserving as much information as possible (maximizing the mutual information) and a limitation on the capacity of sensory encoding (the constraint on the Fisher information). An optimum is achieved when this trade-off results in a coordinate system change in which the Fisher information is constant. This means that the coding noise is a result of an efficient compression of information that maximally preserves the natural statistics in the prior during the coding process.
Formally, we denote by $J(S)$ the Fisher information:

$$J(S = s) = \int \left(\frac{\partial lnp(T = t|S = s)}{\partial s}\right)^2 p(T = t|S = s)dt \quad [10]$$

Wei and Stocker (26, 33) show that the mutual information between the stimulus and the sensory representation $I(S;T)$ can be maximized under the following constraints:

$$C = \int_s \sqrt{J(S = s)}ds \leq C_0, \quad [11]$$

where C_0 is some limited capacity if S has the following form: $T = F^{-1}(F(S) + n)$. The end-result of this analysis is that in the transformed space the prior is uniform. This simple result has an additional justification: in the transformed space the prior is non-informative (it is a Jeffreys prior see (34)). This holds true since if F maps the prior to a uniform distribution then the Fisher information is also constant (26) which satisfies the condition for a Jeffreys prior:

$$P(F(S)) \propto \sqrt{|\det(J(S))|} \quad [12]$$

Discrimination simulations (Fig. 3D). In this analysis, we take as a starting point an estimate for the prior (taken from the last iteration of the transmission chain experiment) in order to predict the results of a separate discrimination experiment. Note that each model (fixed and efficient-encoding) has in this case just one degree of freedom (the magnitude of the model's noise σ). From this we can compute the d' values for the entire image numerically using Equation 7.

In order to produce the simulated d' predictions for Fig. 3D, we performed a grid search over the range of $\sigma = 0 - 0.08$ (relative to an image size of 1). For each value we computed the prediction of the discrimination map and the correlation between the last iteration of the chain experiment and the simulated discrimination experiment. Error bars (blue and red regions) show the standard deviations of the predictions over all the images for each model. Due to edge artifacts produced by the fixed-encoding model's predictions, we exclude values within 6 pixels from the edges of the model predictions as well as the empirical d' maps in order to make a fair comparison between the models and the data. We then recomputed the adjusted correlations between the simulated and real discrimination data and the priors for each of the images, and this reduced the measured correlations for the empirical data (which were in the range $r = 0.45 - 0.63$, see green line in Fig. 3D). However, the correlations between the empirical d' data and the priors after this adjustment were still highly positive ($p < 0.001$ via

bootstrapping), matching the efficient-encoding model predictions. The correlations of the efficient-encoding model with the exclusion were similar to the ones without the exclusion.

Importantly, both models provide a good fit to the empirical chain dynamics given the prior when iterated forward twenty times (Fig. 3A and SI Appendix Fig. S8). Specifically, with the right noise magnitude, both models predict the convergence speed to the prior measured as the Jensen–Shannon divergence (see section on the JSD for formal definition) distance to the prior at each iteration (Fig. S8D), and show consistency with each iteration of the KDE maps for each iteration. To compute the convergence speed of the model data to the prior we estimate the vector of JSD distances $\{c'_j\}_{j=0,1,\ldots,20}$ between each simulated distribution of each iteration P'_j, and the prior P: $c'_j = JSD(P'_j, P)$, where iteration 0 is defined as the initial distribution of the stimulus at iteration 1. Similarly, we can compute the convergence speed for the real data $\{c_j\}_{j=0,1,\ldots,20}$ (see Fig. S5 and SI Appendix for other convergence measures). Fig. S8D shows a plot of this vector for the face image. We fit each model by varying σ so that the L_2 distance between the entire vector of distances is minimized: $score(P'_j) = \Sigma_{j=0,1,\ldots,20}({c'}_j - c_j)^2$, where c and c' are the data and model distances from the prior (for all iterations). We performed a grid search over the noise magnitude. The best fits are displayed in Fig. 3A and SI Appendix Fig. S8. However, only the efficient-encoding model predicts positive correlations between the serial reproduction results and the discrimination experiments (Fig. 3 and SI Appendix Fig. S8A-C). Note that the correlations predicted from the efficient-encoding model were slightly larger than the ones predicted by the empirical discrimination data, possibly due to the empirical reliability of the discrimination map estimates (see analysis of disattenuated correlations in the results section). We also cannot rule out that some small discrepancy between the model predictions and the empirical results are due to perceptual factors that were not modeled in the simulation, such as interference between the memory traces of the stimulus images in the trial sequence or production noise. It is worth noting that the fixed-encoding model with the noise magnitude fitted to the results from the serial reproduction experiments predicts discrimination accuracy maps that deviate from the actual data by simulating a much smaller dynamic range in d' values, and significant edge artifacts. Neither the data nor the efficient-encoding model produced these artifacts (see SI Appendix Fig. S8).

Analysis of consistency of the response bias. Single-trial biases correspond to people's tendency to produce responses that are consistently biased towards a nearby landmark, and that as a result, nearby responses tend to point in the same direction towards the landmark. The symmetric variable precision model predicts that responses are unbiased with respect to the stimulus. Single-trial biases have the following implication for our results: we denote by B the difference between a response R and a stimulus S. From equation 3, we see that B will be a random sample with 0 mean. If the symmetric variable precision model is accurate, this means that if we take neighboring data points in our experiment, we should expect that the difference between the stimulus and response locations will NOT produce response vectors that point in the same direction as they are expected to be independent samples from B (Fig. S3B). On the other hand, if there is a bias (equation 4), where $b(s)$ is a sufficiently large bias parameter that can vary from place to place in the image, we should expect that nearby points with similar $b(s)$ will be biased approximately toward the same direction, for example towards a nearby landmark (see Fig. S3A). To quantify the presence of single-trial biases, we first binned all the data points into bins that subtended 0.04 of the image width and height. We used all the data across all iterations and within each bin we averaged the bias (the differences between response locations and stimulus locations). We only considered bins that contained at least 4 response and stimulus pairs where the estimates are reliable. According to the symmetric variable precision model, we expect these averages to have a random direction and as a consequence, that average responses of adjacent bins will be uncorrelated. However, if there are large single-trial biases then we would expect a different behavior. We would expect the average bias difference vectors in adjacent bins to be pointing consistently in approximately the same direction.

Fig. S3C shows the actual data from the triangle experiment (where the landmark directions are easy to see). We average the response biases inside each bin and plot the direction of the bias (yellow arrows). The empirical data clearly show that average responses in adjacent bins are biased in similar directions. It is visually apparent that biases point toward the landmarks (the modes near the triangle's vertices). On the other hand, the symmetric variable precision model predicts random directions (Fig. S3D), as expected. In these simulations we used variance $\sigma(s)^2$ inversely proportional to the density of the end-state of the serial reproduction experiment, but note that this model would produce random directions regardless of the form and magnitude of the variance. In comparison, the efficient encoding model (with noise value of $\sigma = 0.01$) predicts consistent biases towards the landmarks (Fig. S3E), and it is visually apparent that the model produces single-trial biases that are similar in that respect to the actual human data.

We quantify consistent single-trial biases by measuring the angular differences between the directions of average response vectors in adjacent bins both vertically and horizontally. Fig. S3F shows a histogram of these angular differences. We see that the data (continuous green line) show a clear peak at small angles ($p < 0.001$ via bootstrapping). However, the symmetric variable precision (flat dashed cyan line) predicts a flat histogram of angular differences that is not different from the random distribution (gray). In contrast, the predictions of the efficient encoding model (dashed dark blue line) show a clear peak for small angular differences, which is consistent with the data. We repeated the analysis for all 7 natural images, and obtained similar results (Fig. S3G).

To compute statistical significance of the magnitude of the peak in the histograms and to explore the effect of the noise parameter sigma in both the symmetric variable precision and efficient encoding models, we computed the probability of small angular differences within +/-12 degrees as shown in between the dashed vertical lines in Fig. S3F for the triangle image. We plotted these values as a function of the noise parameter for all natural images and shapes (Fig. S3H). The data show significant small angular differences (horizontal green lines in Fig. S3H). The probability of small angular differences (phases) predicted by the efficient encoding model (dashed dark blue lines) varies with the noise magnitude, but the symmetric variable

precision model (see 7 nearly overlapping cyan lines) predicts no phase consistency regardless of the noise magnitude.

References

1. Ross J, Irani L, Silberman M, Zaldivar A, Tomlinson B (2010) Who are the crowdworkers?: shifting demographics in mechanical turk in *CHI'10 extended abstracts on Human factors in computing systems.* (ACM), pp. 2863–2872.
2. Josephs EL, Konkle T (2019) Perceptual dissociations among views of objects, scenes, and reachable spaces. *Journal of Experimental Psychology: Human Perception and Performance* 45(6):715.
3. Li Y, Hou X, Koch C, Rehg JM, Yuille AL (2014) The secrets of salient object segmentation in *Proceedings of the IEEE Conference on Computer Vision and Pattern Recognition.* pp. 280–287.
4. Koehler K, Guo F, Zhang S, Eckstein MP (2014) What do saliency models predict? *Journal of vision* 14(3):14–14.
5. Wedell DH, Fitting S, Allen GL (2007) Shape effects on memory for location. *Psychonomic Bulletin & Review* 14(4):681–686.
6. Bruce N, Tsotsos J (2006) Saliency based on information maximization in *Advances in Neural Information Processing Systems.* pp. 155–162.
7. Cerf M, Harel J, Einhäuser W, Koch C (2008) Predicting human gaze using low-level saliency combined with face detection in *Advances in Neural Information Processing Systems.* pp. 241–248.
8. Judd T, Ehinger K, Durand F, Torralba A (2009) Learning to predict where humans look in *Computer Vision, 2009 IEEE 12th international conference on.* (IEEE), pp. 2106–2113.
9. Borji A, Sihite DN, Itti L (2013) What stands out in a scene? a study of human explicit saliency judgment. *Vision Research* 91:62–77.
10. Mottaghi R, et al. (2014) The role of context for object detection and semantic segmentation in the wild in *Proceedings of the IEEE Conference on Computer Vision and Pattern Recognition.* pp. 891–898.
11. Li J, Levine MD, An X, Xu X, He H (2012) Visual saliency based on scale-space analysis in the frequency domain. *IEEE Transactions on Pattern Analysis and Machine Intelligence* pp. 1–1.
12. Holden M, Shipley T, Newcombe N (2007) Memory for location is influenced by part-based segmentation of space. *Journal of Vision* 7(9):914–914.
13. Holden MP, Curby KM, Newcombe NS, Shipley TF (2010) A category adjustment approach to memory for spatial location in natural scenes. *Journal of Experimental Psychology: Learning, Memory, and Cognition* 36(3):590.
14. Holden MP, Newcombe NS, Shipley TF (2013) Location memory in the real world: Category adjustment effects in 3-dimensional space. *Cognition* 128(1):45–55.
15. Suchow J, et al. (2019) Fully automated behavioral experiments on cultural transmission through crowdsourcing. *Collective Intelligence 2019.*
16. Dreyfuss E, Barrett B, Newman LH (2018) A bot panic hits amazon's mechanical turk.
17. Rehrig G, Peacock CE, Hayes TR, Henderson JM, Ferreira F (2020) Where the action could be: Speakers look at graspable objects and meaningful scene regions when describing potential actions. *Journal of Experimental Psychology: Learning, Memory, and Cognition.*
18. Henderson JM, Hayes TR, Rehrig G, Ferreira F (2018) Meaning guides attention during real-world scene description. *Scientific reports* 8(1):1–9.
19. Henderson JM, Hayes TR (2017) Meaning-based guidance of attention in scenes as revealed by meaning maps. *Nature Human Behaviour* 1(10):743–747.
20. Canny J (1986) A computational approach to edge detection. *IEEE Transactions on Pattern Analysis and Machine Intelligence* (6):679–698.
21. Harris C, Stephens M (1988) A combined corner and edge detector. in *Alvey vision conference.* (Citeseer), Vol. 15, pp. 10–5244.
22. Shi J, , et al. (1994) Good features to track in *1994 Proceedings of IEEE conference on computer vision and pattern recognition.* (IEEE), pp. 593–600.
23. Bradski G (2000) The OpenCV Library. *Dr. Dobb's Journal of Software Tools.*
24. Griffiths TL, Kalish ML (2005) A bayesian view of language evolution by iterated learning in *Proceedings of the 27th Annual Meeting of the Cognitive Science Society.* Vol. 27.
25. Huttenlocher J, Hedges LV, Duncan S (1991) Categories and particulars: prototype effects in estimating spatial location. *Psychological Review* 98(3):352.
26. Wei XX, Stocker AA (2015) A bayesian observer model constrained by efficient coding can explain 'anti-bayesian' percepts. *Nature Neuroscience* 18(10):1509.
27. Wei XX, Stocker AA (2017) Lawful relation between perceptual bias and discriminability. *Proceedings of the National Academy of Sciences* 114(38):10244–10249.
28. Penfield W, Boldrey E (1937) Somatic motor and sensory representation in the cerebral cortex of man as studied by electrical stimulation. *Brain* 60(4):389–443.
29. Tootell RB, Switkes E, Silverman MS, Hamilton SL (1988) Functional anatomy of macaque striate cortex. ii. retinotopic organization. *Journal of Neuroscience* 8(5):1531–1568.
30. Pennec X (1999) Probabilities and statistics on riemannian manifolds: Basic tools for geometric measurements. in *NSIP.* (Citeseer), Vol. 3, pp. 194–198.

31. Tenenbaum JB, De Silva V, Langford JC (2000) A global geometric framework for nonlinear dimensionality reduction. *Science* 290(5500):2319–2323.
32. Van Den Berg R, Shin H, Chou WC, George R, Ma WJ (2012) Variability in encoding precision accounts for visual short-term memory limitations. *Proceedings of the National Academy of Sciences* 109(22):8780–8785.
33. Wang Z, Wei XX, Stocker AA, Lee DD (2016) Efficient neural codes under metabolic constraints in *Advances in Neural Information Processing Systems.* pp. 4619–4627.
34. Jeffreys H (1946) An invariant form for the prior probability in estimation problems. *Proceedings of the Royal Society of London. Series A. Mathematical and Physical Sciences* 186(1007):453–461.

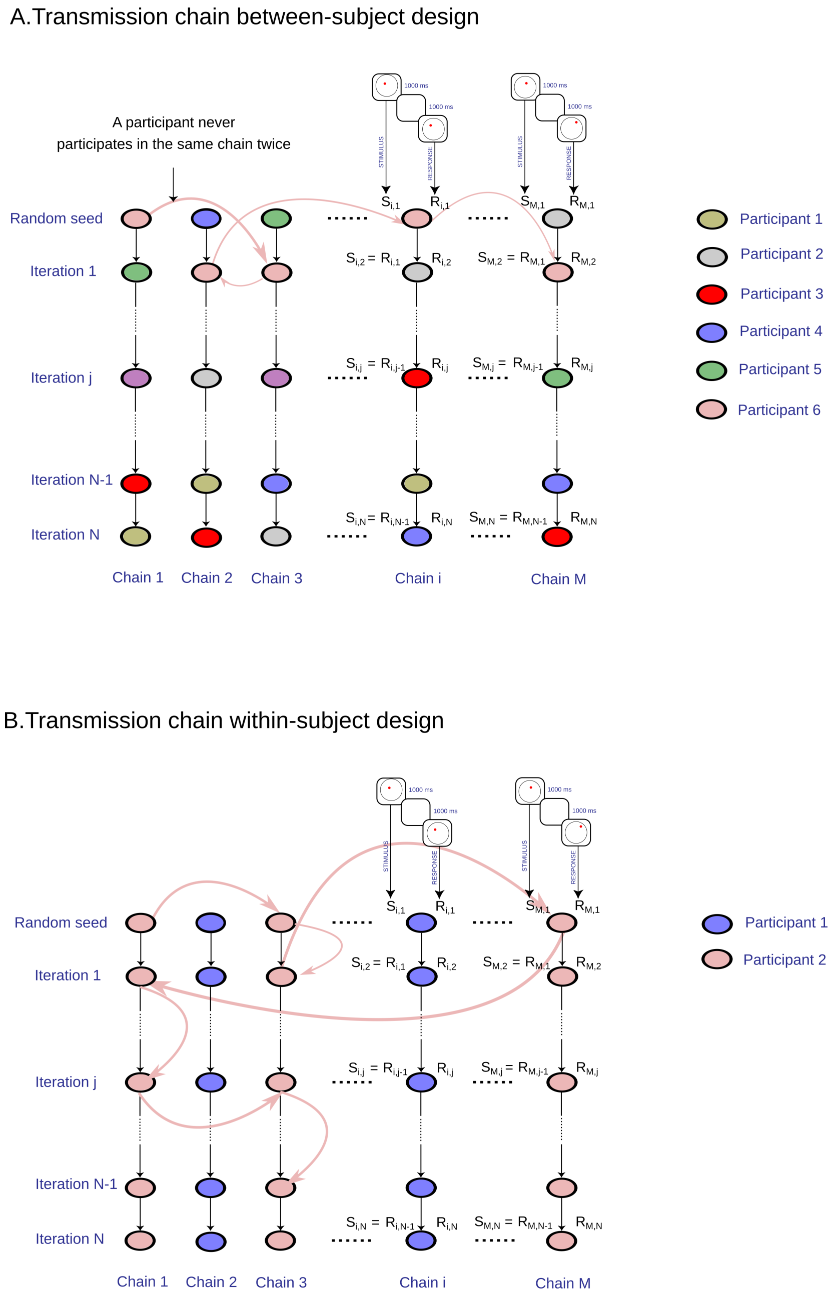

Fig. S1. Serial reproduction experiment designs: The between-subject and within-subject designs. A. Between-subject design. Each chain was composed of nodes representing individual trials. Each trial contained a stimulus $S_{i,j}$, delay, and response $R_{i,j}$. Chains contained $N = 20$ iterations. Each chain began with an initial seed point location sampled from a uniform distribution, and subsequent nodes in the chain contained the response to the previous node as the stimulus (the "telephone game" procedure). Participants were randomly assigned to trials in different chains and never participated in the same chain twice. B. Within-subject design. Participants completed entire chains, alternating between nodes in their assigned chains. In this design, no chain contained data from more than a single participant.

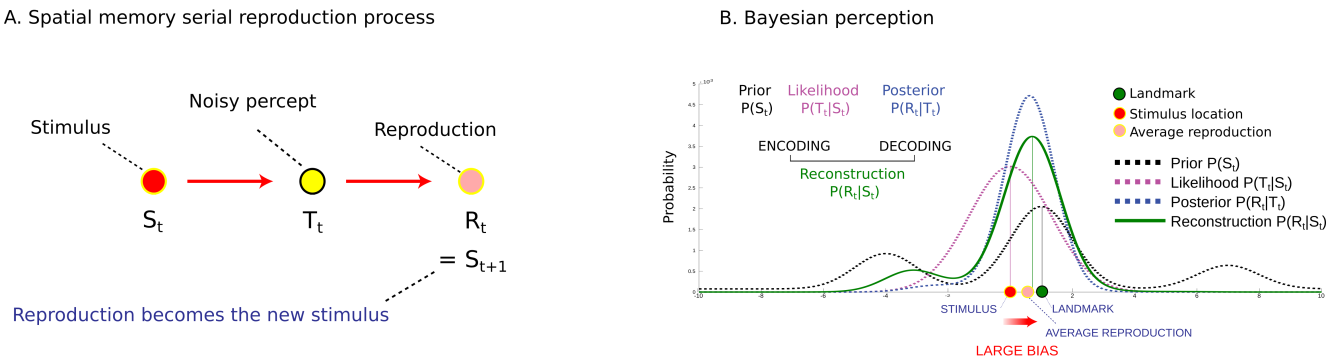

Fig. S2. A. Spatial memory serial reproduction process. A stimulus point location S_t is perceived as a noisy percept T_t and reproduced as a location R_t. This reproduced point becomes the stimulus for the next participant in the serial reproduction chain (S_{t+1}). B. Bayesian perception. A stimulus location is remembered following an inference process during which a noisy percept of the actual location (the likelihood $P(T_t|S_t)$, purple dotted line) is integrated with a belief state about probable point locations (the prior $P(S_t)$, dotted black line) resulting in the posterior ($P(R_t|T_t)$, blue dotted line). The reconstruction (the net result of the encoding and decoding) $P(R_t|S_t)$ is shown in the solid green line. A stimulus point location (red dot) near a landmark (green dot) will tend to be misremembered with a bias towards the landmark, and on average will be reproduced closer to the landmark (pink dot).

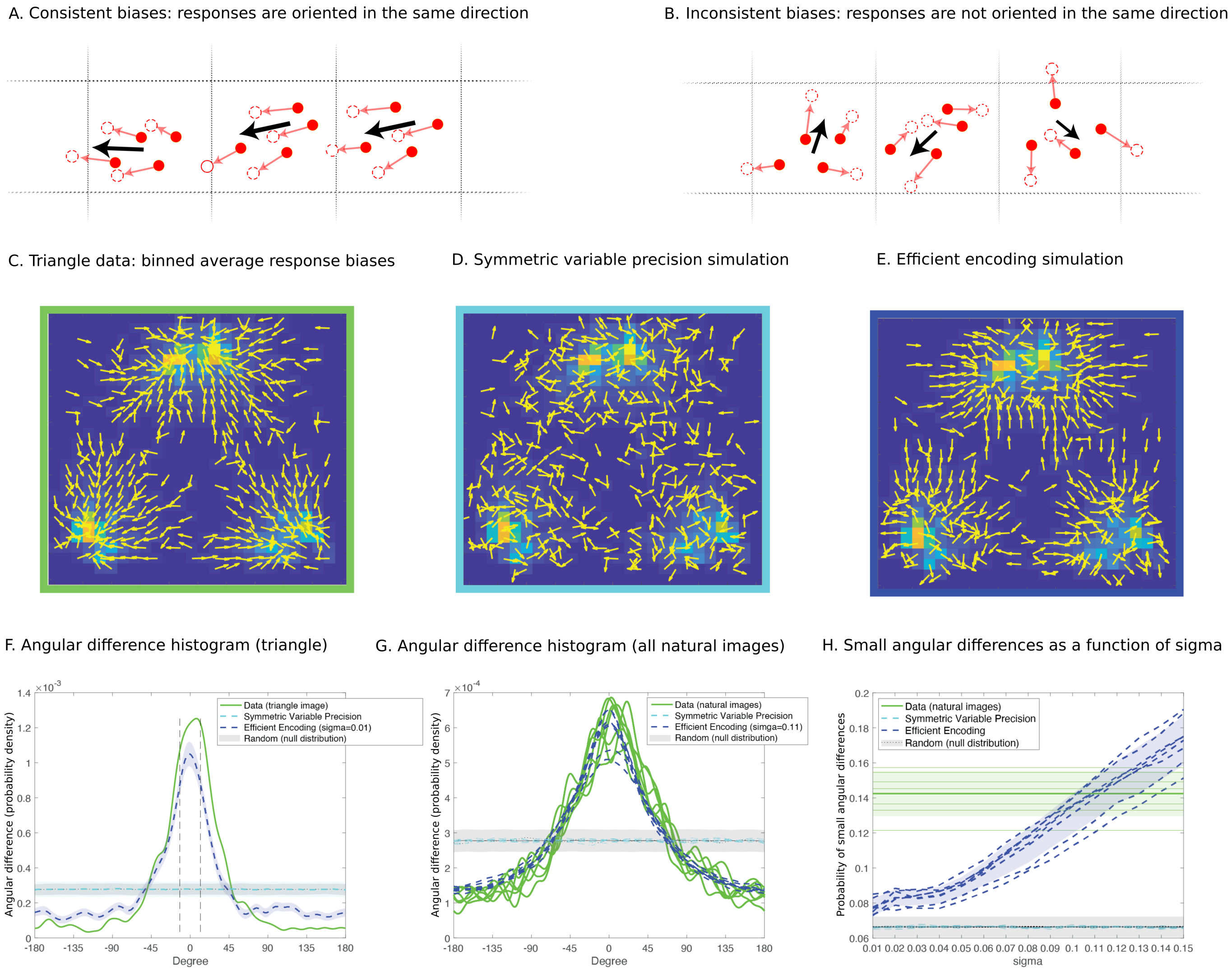

Fig. S3. Analysis of the consistency of the bias. A. Prediction for consistent biases. Average response vectors (black arrows) for nearby point reconstructions (white dots with red outline) will tend to point in the same direction. B. Prediction for inconsistent (random) biases in responses. Average response vectors (black arrows) for nearby point reconstructions will not point in the same direction. C. Coherent biases in the triangle data. We averaged all differences between response and stimulus pairs across all iterations that fell within bins that subtended 0.04 of the image width and height, in a grid over the image. We computed the direction of the average bias (yellow arrows). The direction vectors are plotted on top of the KDE of the last iteration, which we downsampled to the grid resolution. D. The symmetric variable precision model predictions. Directions are incoherent (random). E. The efficient encoding model predictions. The model qualitatively replicates the pattern seen in the data. F. Quantification of the bias consistency. Angular difference histogram for the triangle image. The x-axis shows the angular difference (in degrees) of the average directions of the biases in adjacent bins. The angular differences for the actual data (green line) and efficient encoding model (simulated dashed dark blue line) are concentrated near 0 degrees, showing significant consistent biases ($p < 0.001$ via bootstrapping). The symmetric variable precision model (cyan line) is not different from the uniform distribution (gray area). Shaded areas show one standard deviation of the histograms computed by simulating the models 1000 times. G. Results for all natural images. H. Small angular differences (probability of angular differences between -12 and 12 degrees) as a function of model and noise magnitude. The efficient encoding model (dashed blue lines) predicts different levels of bias consistency depending on the noise magnitude but the symmetric variable precision model (overlapping cyan lines) predicts random phases regardless of noise magnitude that are not different from the uniform distribution (shaded gray area).

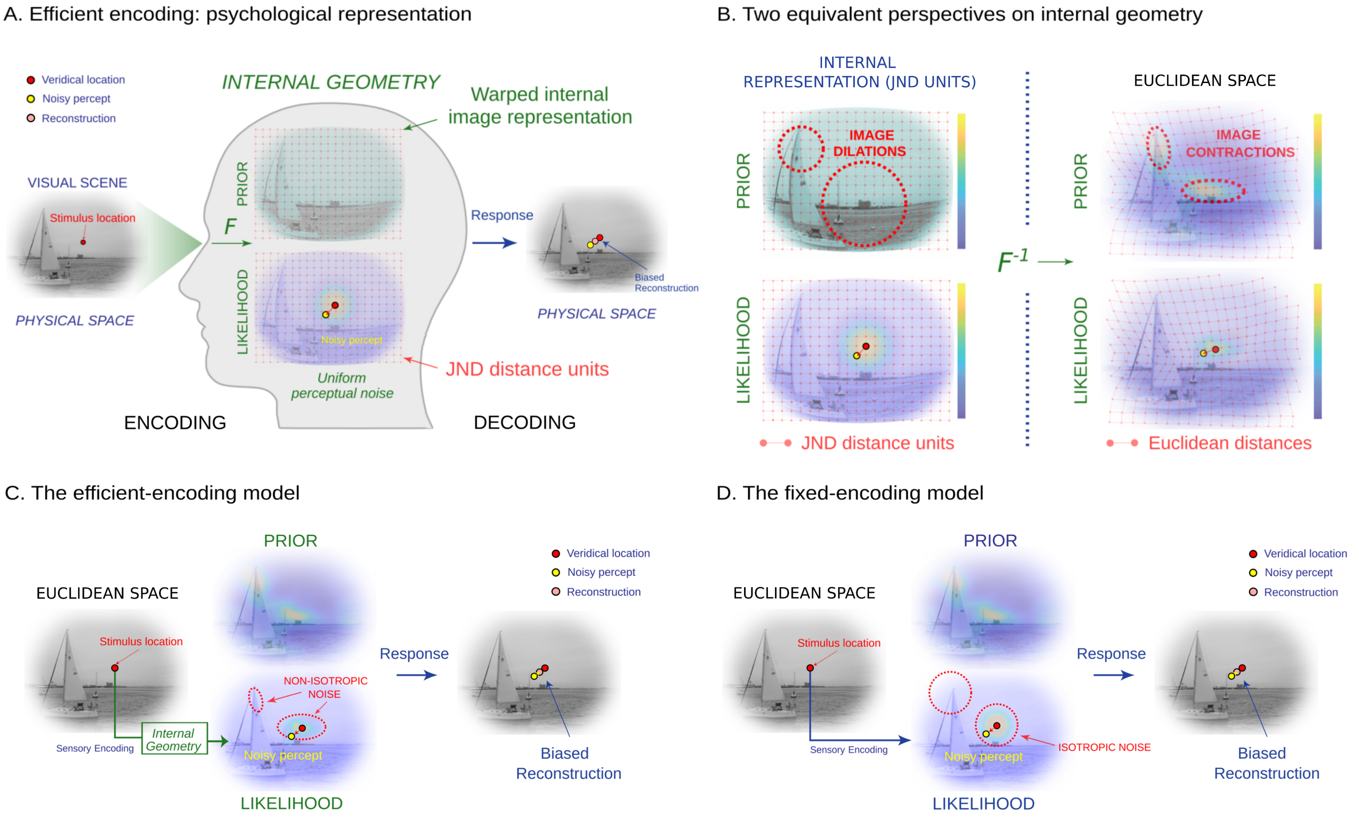

Fig. S4. Rational models of visuospatial memory. A. The efficient-encoding model. Variations in sensory noise encode informative visual regions with higher resolution, resulting in warped internal representations where some visual regions are over-represented relative to others (“internal geometry” of the psychological representation produced by the function F). In internal just-noticeable difference (JND) units, perceptual noise is isotropic and Gaussian, and the prior is uniform. The distortion of the internal scene representation is inversely proportional to the density in the prior. In panels A-D, original location (red point) is inferred by combining the prior with a noisy percept (yellow point), and the participant produces a biased reconstruction (pink point). B. Two equivalent perspectives on the psychological representation. According to the model, the same internal distribution can be described in two equivalent coordinate systems. The deterministic function F maps Euclidean distances to a representation in internal JND distance units, while F^{-1} inverts this representation, transforming JNDs back to Euclidean distances (Euclidean space). In JND units the prior is uniform and the likelihood is Gaussian. This implies that in the Euclidean space, the prior is non-uniform and the likelihood varies across parts of the image. C. The “efficient-encoding” model. Unlike in the “fixed-encoding” model, encoding precision varies systematically depending on the location in the visual scene. D. The “fixed-encoding” model. Encoding precision is fixed throughout the image.

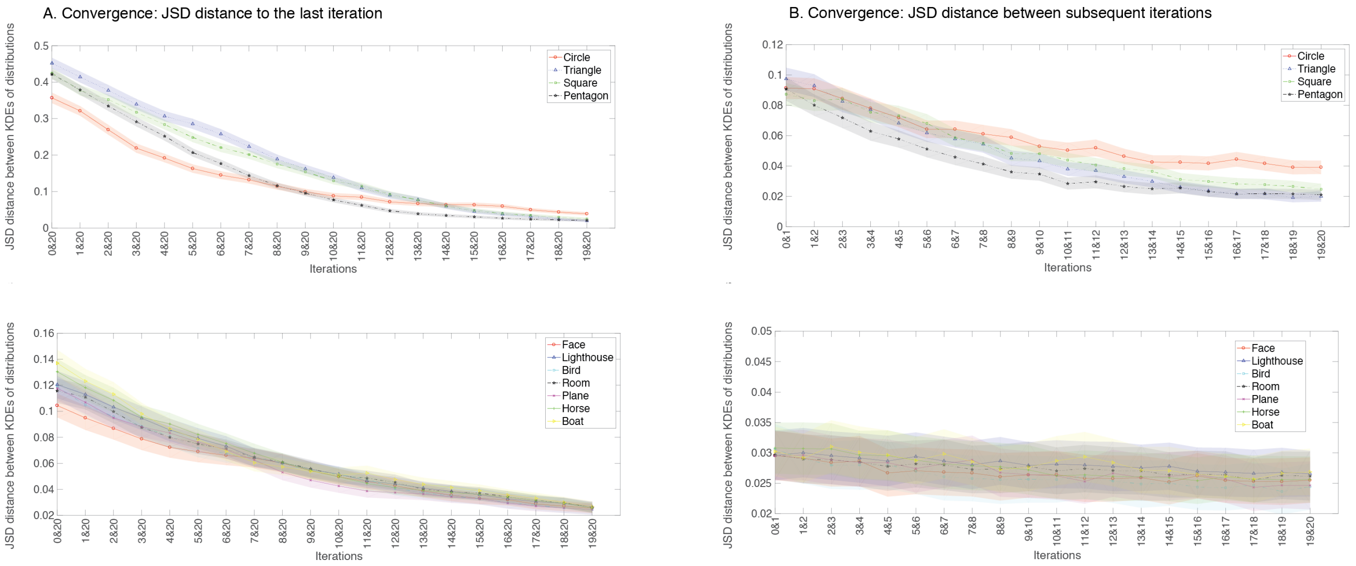

Fig. S5. Convergence using JSD. The shaded regions correspond to standard deviations from 1000 KDEs obtained from bootstrapped samples. We denote by “iteration 0” the initial seed distribution. A. JSDs between distributions of each iteration and the final iteration distributions. B. JSDs between subsequent iterations.

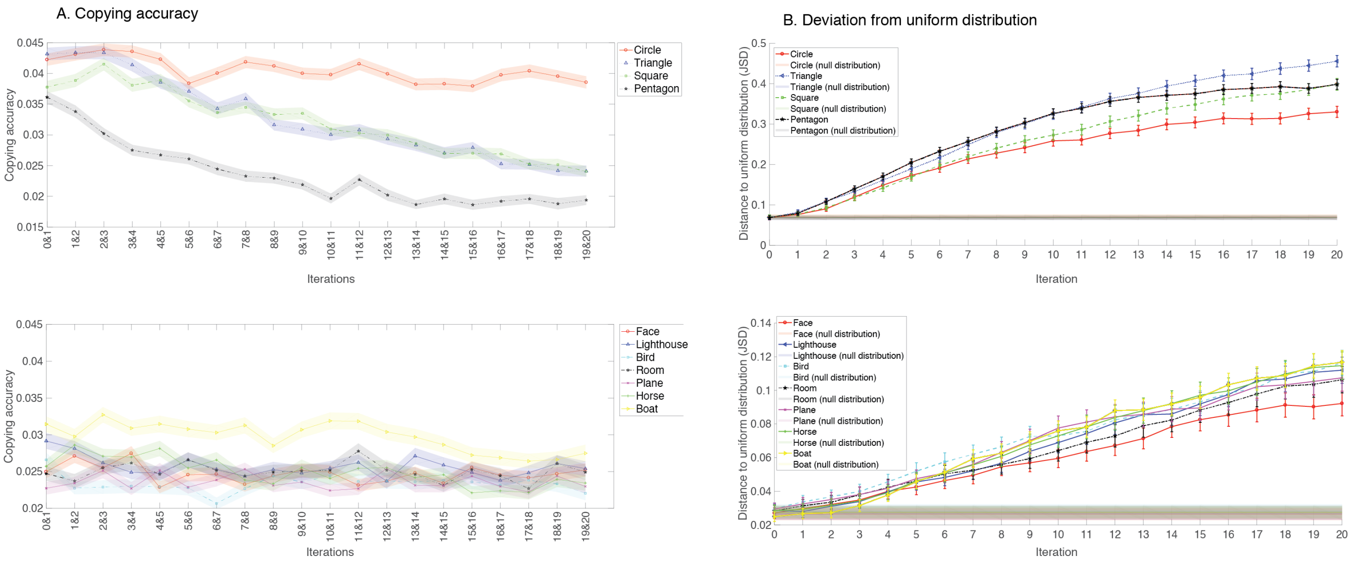

Fig. S6. A. Copying accuracy, computed by the root mean squared Euclidean distance (in normalized units). The shaded regions correspond to standard deviations from 1000 datasets randomized with replacement from the experimental data. We denote by iteration 0 the initial seed distribution. B. Deviation from uniformity. JSD distance from randomized samples of a uniform distribution. The shaded regions correspond to standard deviations from 1000 KDEs obtained from bootstrapped samples.

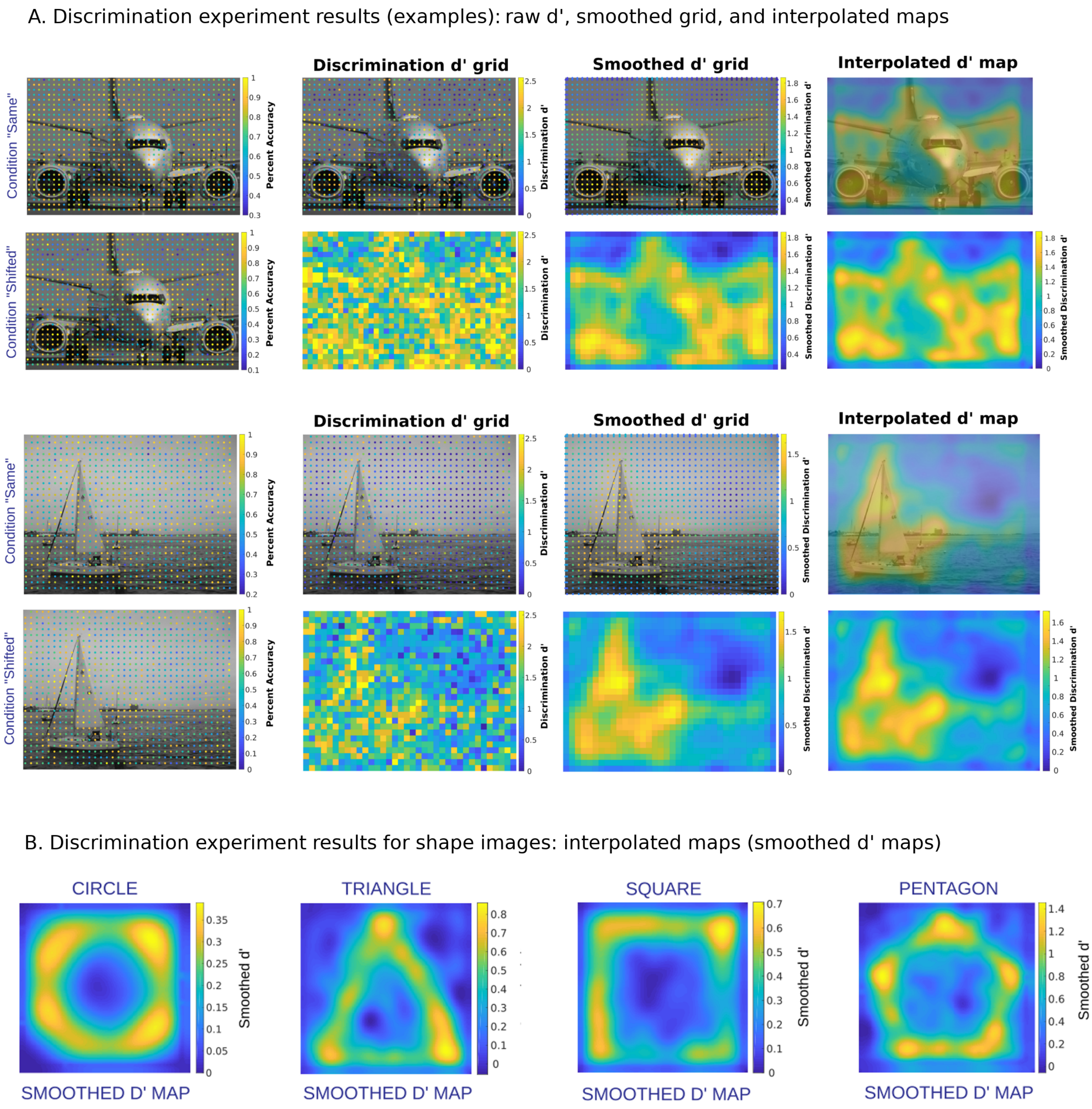

Fig. S7. Discrimination maps: Natural images and shape images. A. Discrimination percent accuracy for "same" and "shifted" conditions for the plane image (top left), including raw d' grid point values, smoothed d' in top right row, plotted over the image, and shown without the image (in the second row). The interpolated d' map is also shown in the far right column, overlayed over the image, and without the image. Examples are also shown for the boat image. B. Smoothed d' maps for shape images.

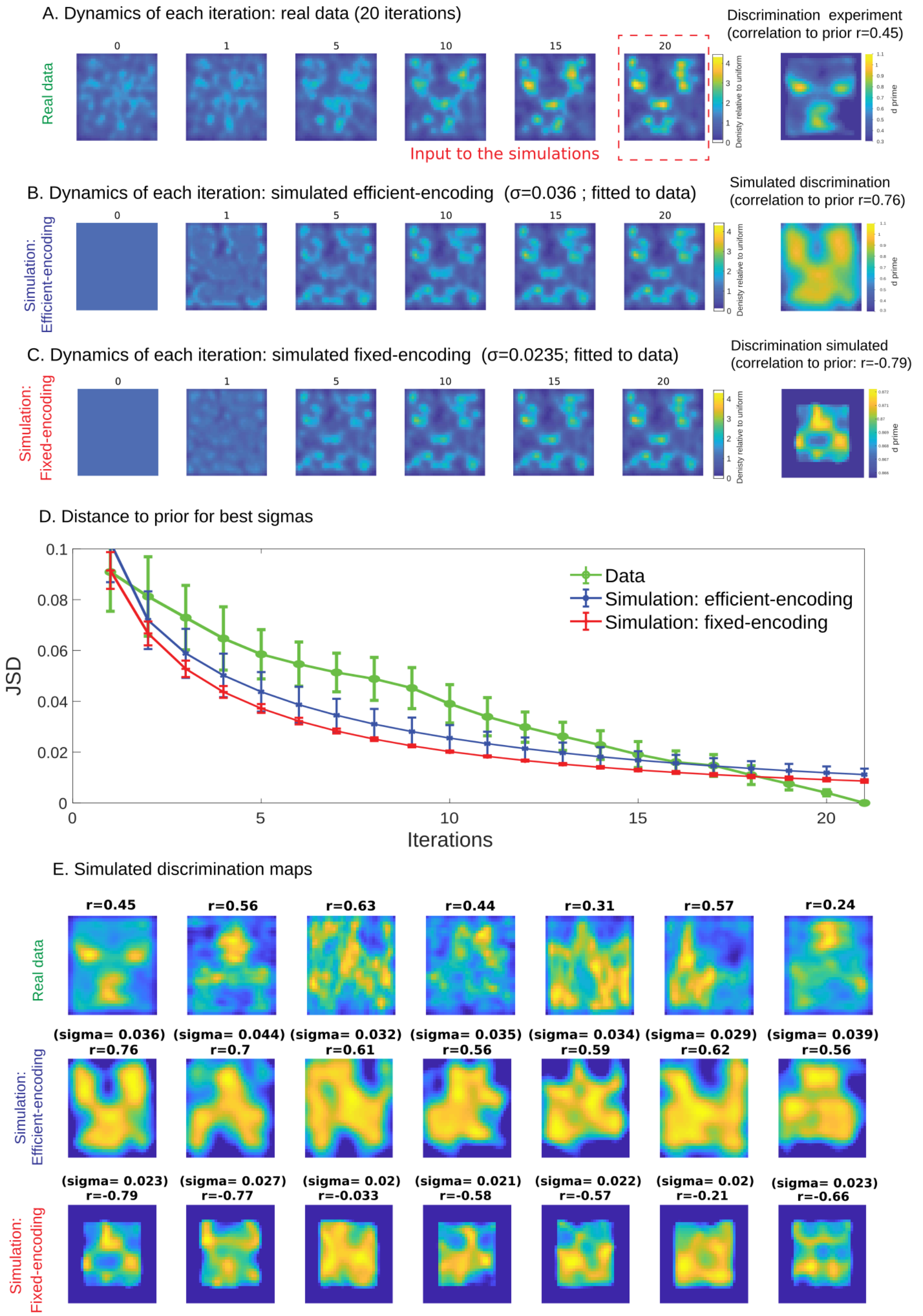

Fig. S8. Representative example of the simulated chain dynamics for the efficient-encoding and fixed-encoding models, and real chain results (face image). We also show simulated d' maps that are predicted from the serial reproduction experiment and compare them to the actual d' results. A. Dynamics of the empirical serial reproduction experiment. Each panel shows a KDE fit to the point locations at each iteration, where iteration 0 corresponds to the initial uniform distribution. Also shown are the smoothed discrimination experiment d' results. Discrimination is positively correlated with the KDE fit to the data in the last iteration of the chains. B. Simulation of the chain dynamics using the efficient-encoding model. The input for the simulation was the empirical prior obtained from the serial reproduction experiment (KDE fit to the data in the last iteration, shown inside the dotted red bounding box in A). C. Dynamic simulation for the fixed-encoding model. D. Fitting the noise parameters based on the serial reproduction experiment. The graph shows the JSD distance between the prior (final iteration KDE), and the model predictions at each iteration (blue and red lines), and empirical chain iterations (green line). We performed a grid search over the magnitudes of the noise parameter (σ). We selected the value for the noise magnitude that produced chain dynamics that most closely approximated the empirical dynamics (green curve) in panel D. The best values were 0.036 and 0.0235 for the efficient encoding and fixed encoding model, respectively. E. Simulated d' results for the efficient-encoding and fixed-encoding models and empirical d' maps. Top row shows empirical d' results and correlations to the corresponding priors. Second row shows the efficient-encoding model predictions. Correlations of the predicted discrimination maps to the prior KDEs (displayed above each image) are all positive for the efficient-encoding model. Bottom row shows discrimination predictions of the fixed-encoding model. Correlations between the predicted discrimination maps and the prior KDEs are all negative at the fitted noise level for the fixed-encoding model. Note that the fixed-encoding model produced edge artifacts (which were not observed in the data). To facilitate the comparison, we excluded values 6 pixels from the edges of the predicted discrimination maps when computing the correlations

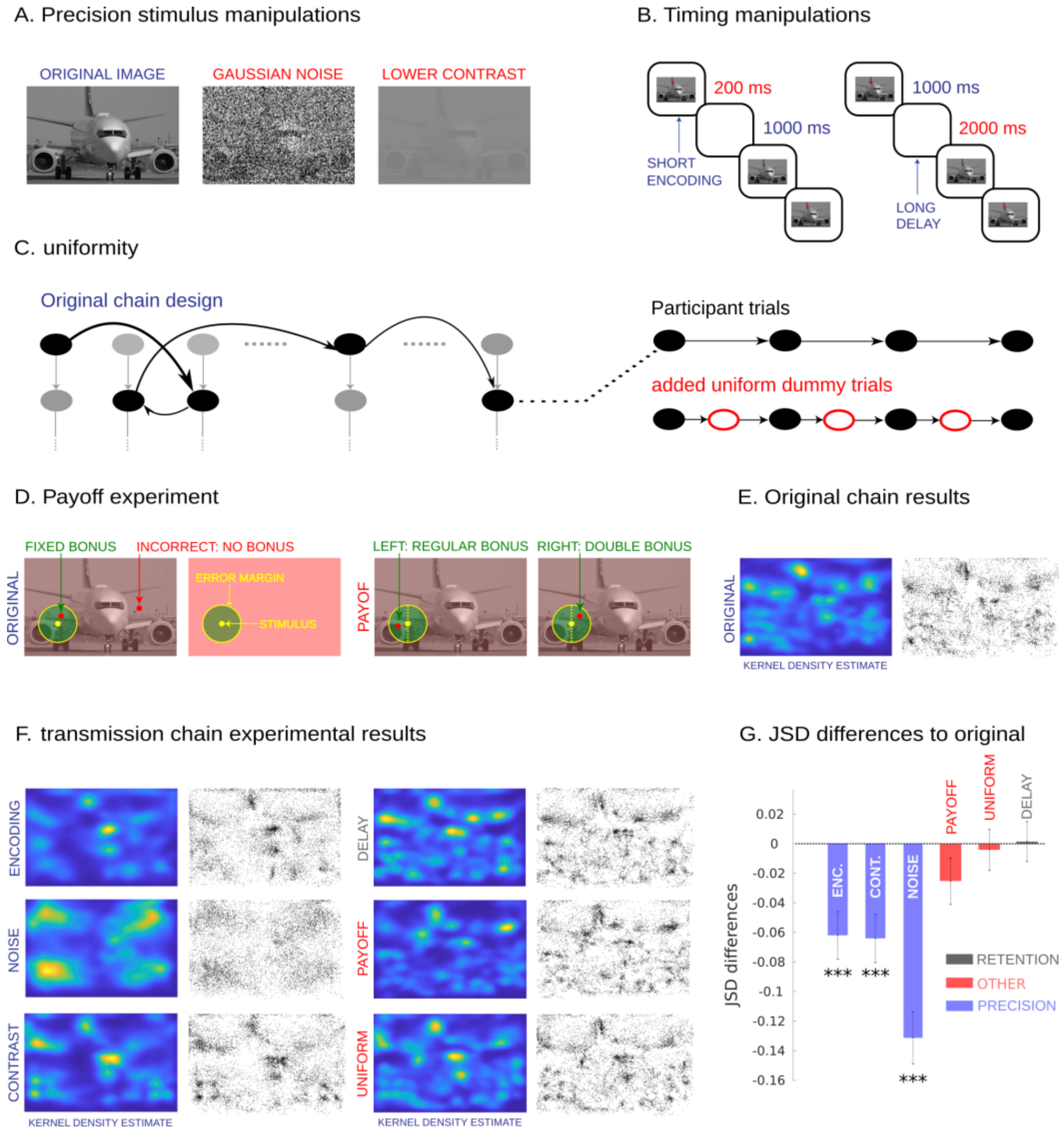

Fig. S9. Encoding precision direct experimental manipulations. A. Encoding precision stimulus manipulations. We repeated the serial reproduction experiment with manipulations to the image by adding Gaussian noise or reducing the contrast. B. Timing manipulations. We repeated the serial reproduction experiment with a reduced presentation period (200ms instead of 1000ms), or a longer delay period (2000ms instead of 1000ms). C. Other manipulations: We tested the Markovian assumption by interleaving experimental trials with dummy trials in which points where presented in random locations. D. Other manipulations: payoff experiment. We tested the effect of introducing a payoff to the task by rewarding accurate responses to the right of the original stimulus with double the bonus awarded to accurate responses to the left of the original stimulus. E. The KDE and scatterplot of the original serial reproduction experiment results. F. Results of the precision, delay and other manipulations: scatterplots and KDEs. G. JSD differences comparing KDEs from each of the manipulations to the original results. Results show differences are significantly different from zero ($p < 0.001$) in the case of the precision manipulations (blue bars), and not significantly different from zero in the case of the delay and other manipulations (red and gray bars, respectively).

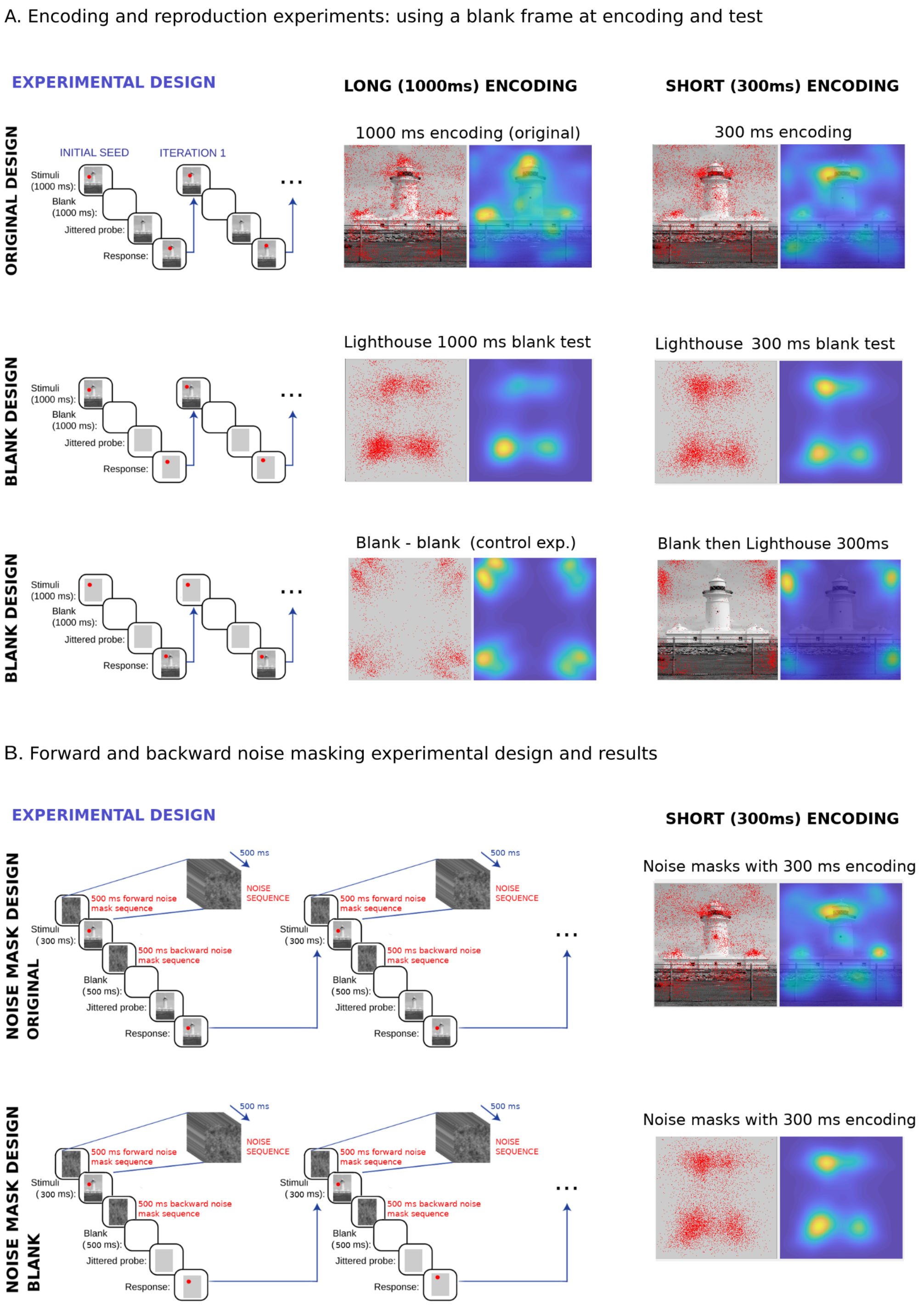

Fig. S10. A. Encoding and reproduction experiments. In order to confirm that biases emerge during the encoding phase, and not during the reproduction phase, we compared the results of substituting a natural image with a blank (uniform gray) probe image at test time, or during the encoding phase. A. The experimental design of the original experiment, as well as the two manipulations, and results using a 1000 ms encoding duration, or a short 300 ms encoding duration. Results clearly show that the prior is biased towards the landmarks of the images presented during the encoding phase and not the reproduction phase. The results of a control experiment, in which the blank gray frame was presented both during the encoding phase and the reproduction phase is also shown. B. Forward and backward noise masking experimental design and results. We introduced forward and backward masking to the encoding phase of the original experimental design, and reduced the encoding time to 300 ms instead of the full 1000 ms. The masks were generated as a sequence of random $1/f$ "pink" noise images. We also ran an experiment in which we substituted the natural image with a blank image for the reproduction (response) phase. The results of the first experiment show that masking had little to no effect on the outcome of the experiment relative to the same experiment without masking. Finally, the results of the second experiment using a blank frame during the response phase reveals that masking had little to no effect on the outcome of the experiment relative to the same experiment without the masking. These findings suggest that overt attention in the form of eye movements during encoding are likely not responsible for the patterns of biases. They also show that eye-movements at test time cannot explain the patterns of biases either.

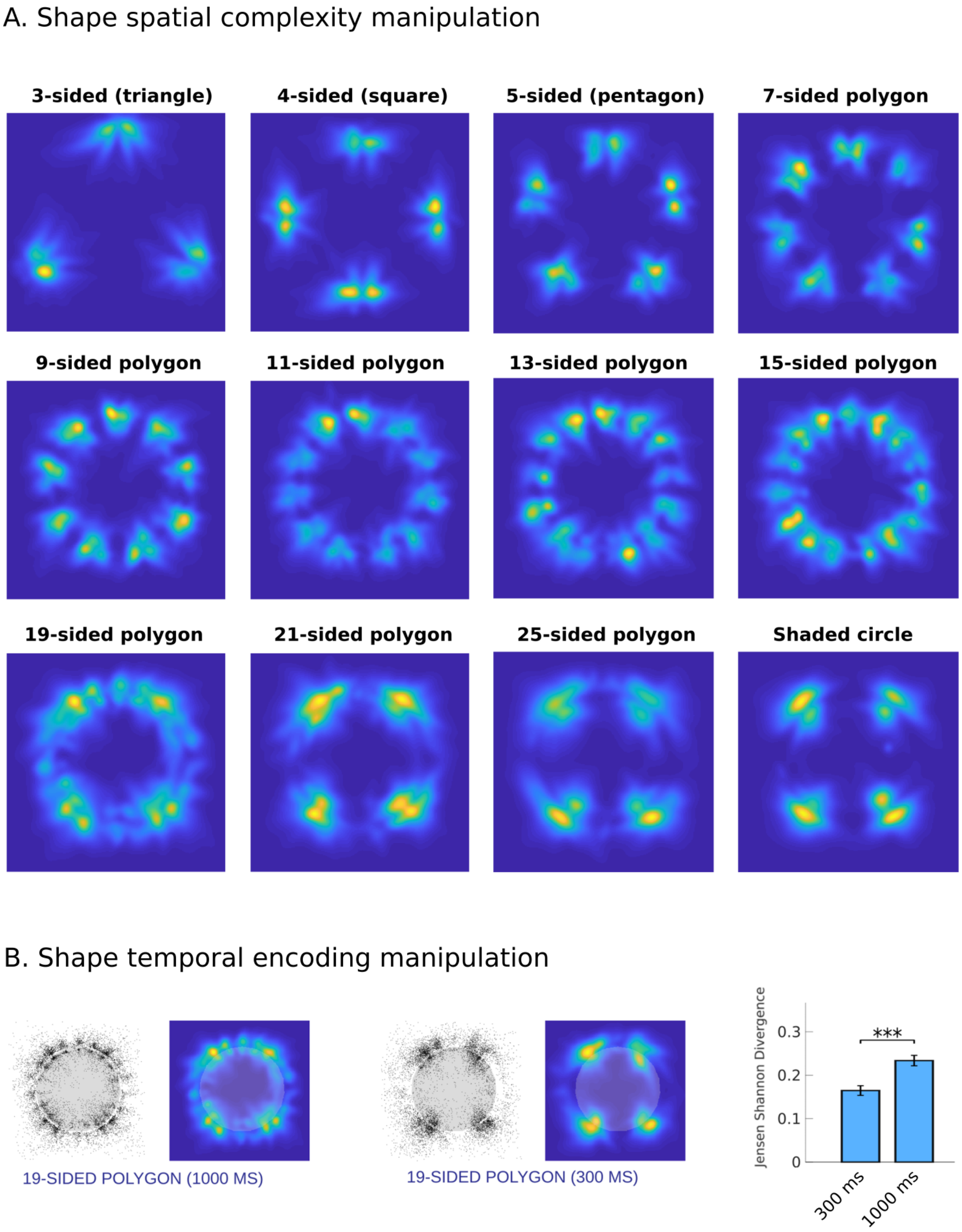

Fig. S11. A. Spatial manipulation of encoding precision. The apparent increase in peaks in spatial memory KDEs for more complex regular polygons led us to consider changes to the internal representation in the limit, as the regular polygons become more complex and start to approximate a circle. Each panel shows the non-parametric kernel density estimates (KDE) of the serial reproduction results obtained using regular polygon images with an increasing number of corners. As the image complexity increases, the KDE structures begin to resemble the results for the circle. B. Temporal encoding precision manipulation: When the presentation time of a 19-sided regular polygon stimulus image is reduced from 1000 to 300 ms, the resulting biases are significantly simplified towards the spatial memory representation for a circle. The barplot shows the relative differences in the effect of changing the encoding time on the KDE structure using the JSD. All distances were computed as the JSD distances between the final distribution of points for both manipulations and the final distribution for a shaded circle. Error bars represent the standard deviation of the distance estimated by bootstrapping (after applying the Bonferroni correction; *: $p < .05$; **: $p < .01$; ***: $p < .001$).

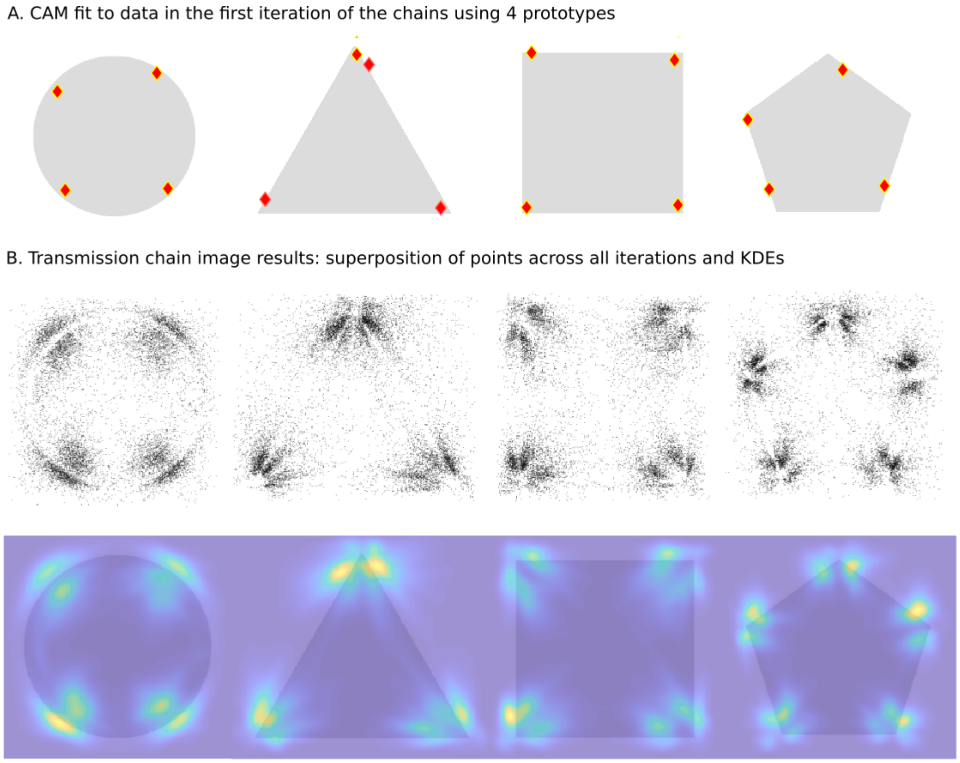

Fig. S12. Category Adjustment Model (CAM) estimates for simple shape images. The CAM asserts that each reconstruction from memory linearly interpolates between the stimulus and a prototype (see methods). We fit the CAM using 4 prototype location terms to the data for each of the shapes, using the exact same procedure as (5). We used all initial point locations and the positions in the first iteration for each of the images. B. Scatter plots showing the superposition of responses across all iterations of the chains for each of the shapes, and the corresponding Kernel Density Estimates (KDEs).

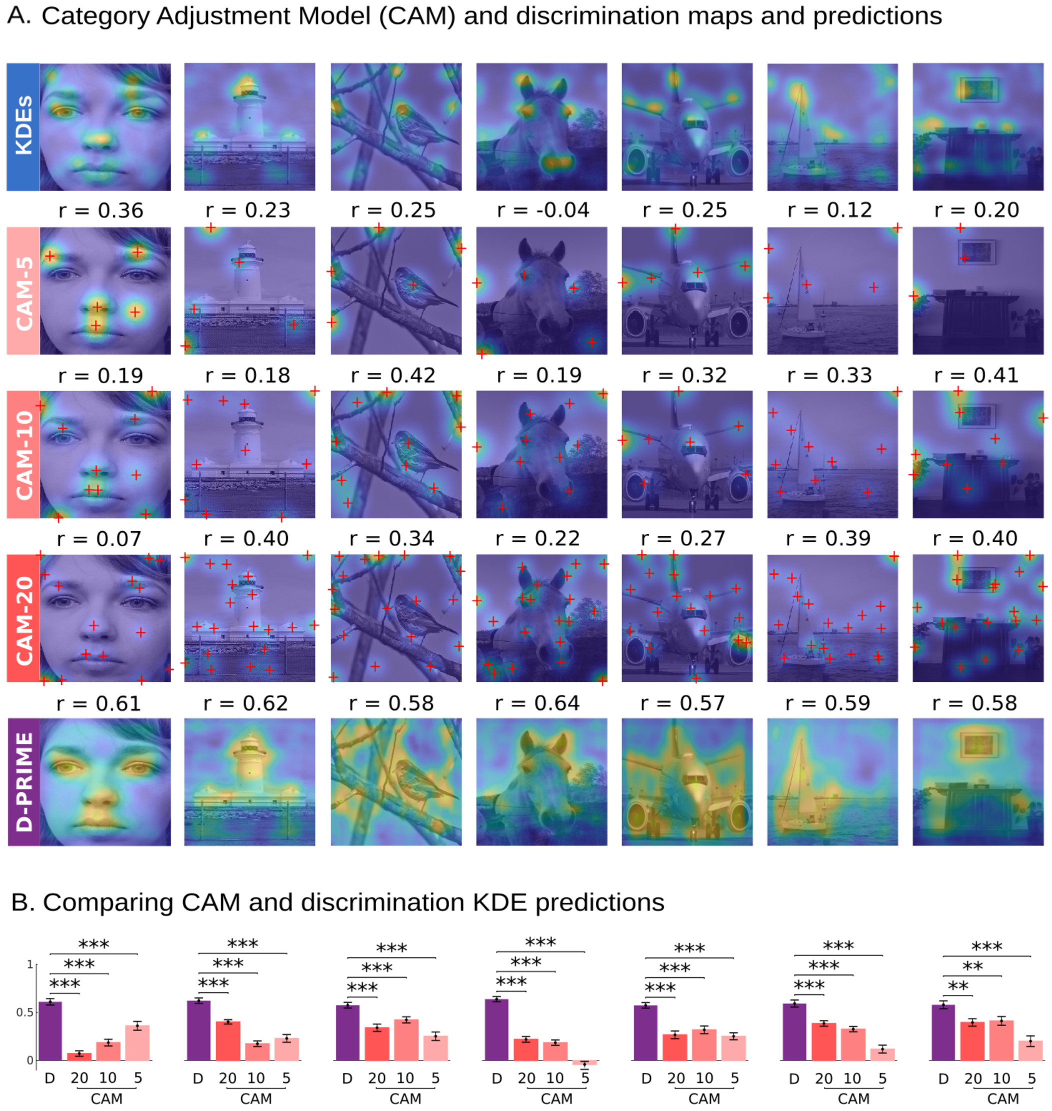

Fig. S13. Category Adjustment Model (CAM). This model asserts that each reconstruction from memory linearly interpolates between the stimulus and a prototype (see methods). A. We fit the CAM using 5, 10, and 20 prototype location terms to the data for each of the natural images. We used all initial point locations and the positions in the first iteration for each of the images. B. Correlations of KDEs fit to the CAM point-estimates to KDEs of the chain data are shown, as well as correlations between chain KDEs and smoothed discrimination d' maps for comparison. In some cases, the model produced estimates of the prototype locations that were nearly overlapping.

A. Split-half chain sampling, KDE and CAM model-fitting procedure, and comparison (r) for one split-half sample

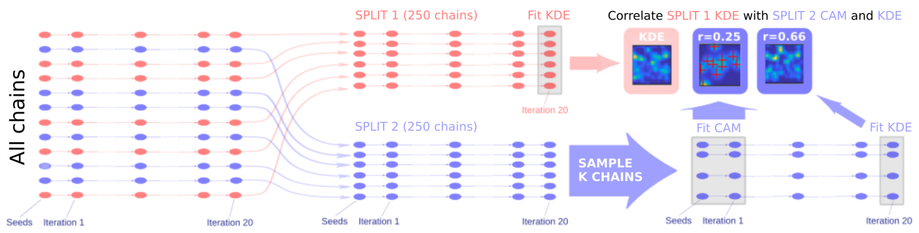

B. Plane image: comparison between split-half KDE reliability, and CAM estimate reliability (100 split-half samples)

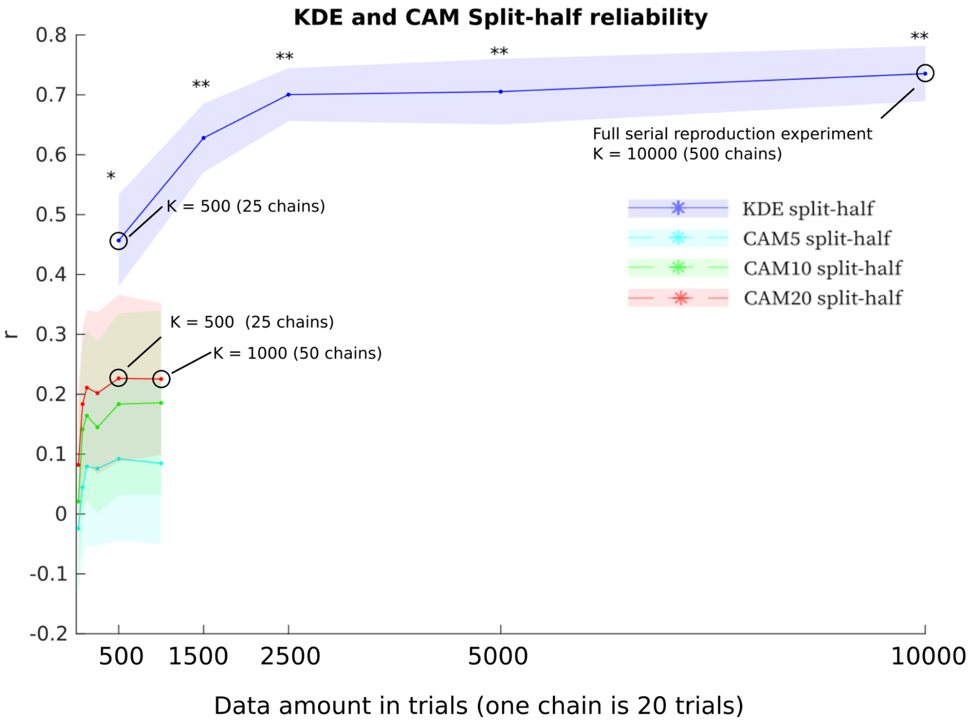

Fig. S14. CAM and serial reproduction chain reliability analysis. A. For a given random partition of the data into two equal splits (split 1 in red, and split 2 in blue), we fit a KDE to the 20th iteration data in split 1 and compared it to a KDE fit to data in K chain samples from split 2, as well as CAM estimates fit to the same K chain samples in split 2. B. Results comparing the internal reliability of the KDEs fit to random splits of the data (blue line), as well as the reliability of the CAM estimates, using 5 (cyan line), 10 (green line), and 20 (red line) prototypes, for each value of K samples from the 2nd partition of 250 chains. The shaded error bars correspond to 100 random partitions of the full chains into two equal parts. For all values of K, the internal reliability estimates of the KDEs were significantly higher than reliability of the CAM estimates. The x-axis in the graph is normalized according to the number of participant trials used for the estimation (equating for the fact that the serial reproduction estimates are made from multiple iterations, and therefore more data). The analysis indicates that even when the two methods are equated for the amount of data used, the serial reproduction results produce more reliable estimates.

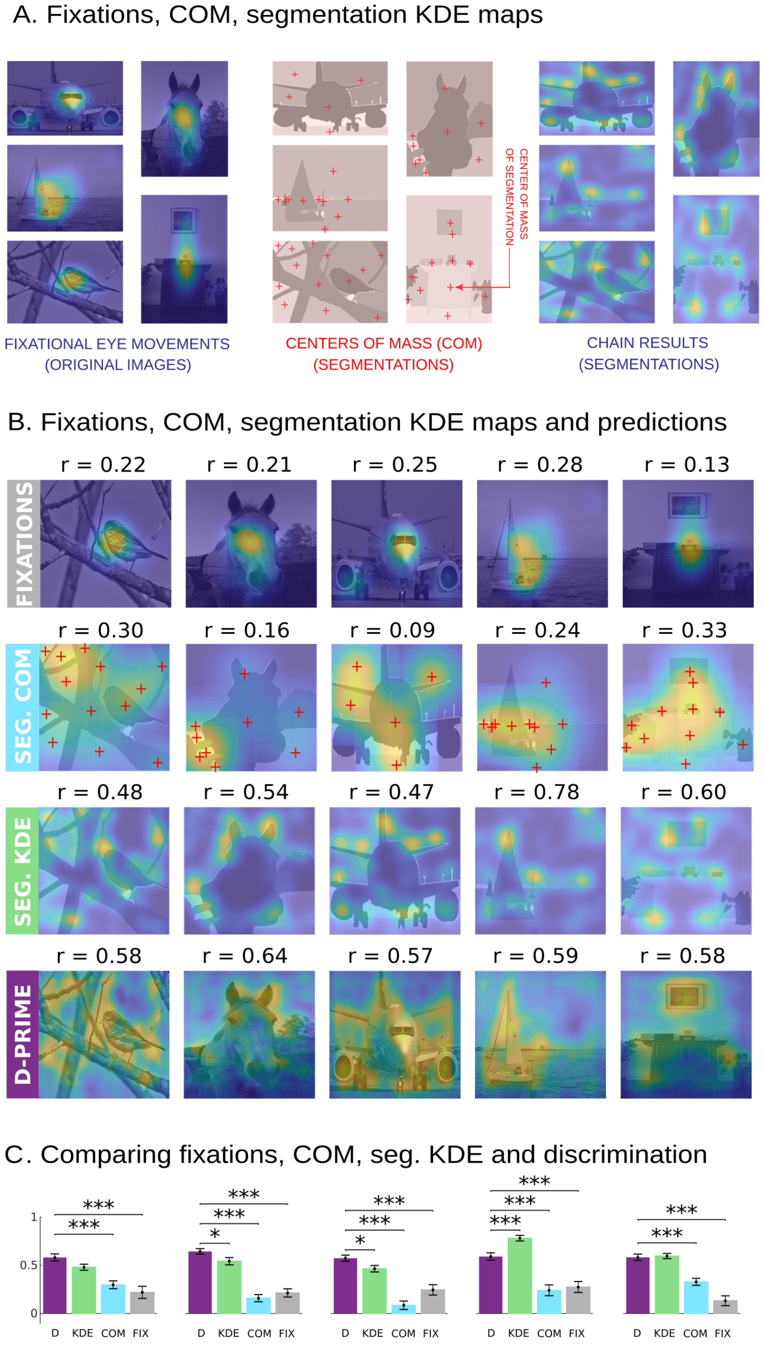

Fig. S15. KDE predictions using Centers of Mass (COM), Fixations, and Segmentations. A. Eye-movements obtained for the natural images, centers of mass (COM) computed as the average x and y coordinate values inside each of the human-made segmentations of the objects in the images, and transmission chain results (KDEs) obtained using segmentation images instead of original grayscale images. B and C. Model comparison comparing discrimination maps, KDEs fit to transmission chain data obtained for segmentation images, COM, and fixations. The model comparison shows that the COMs and fixations were the weakest predictors of the original image KDEs

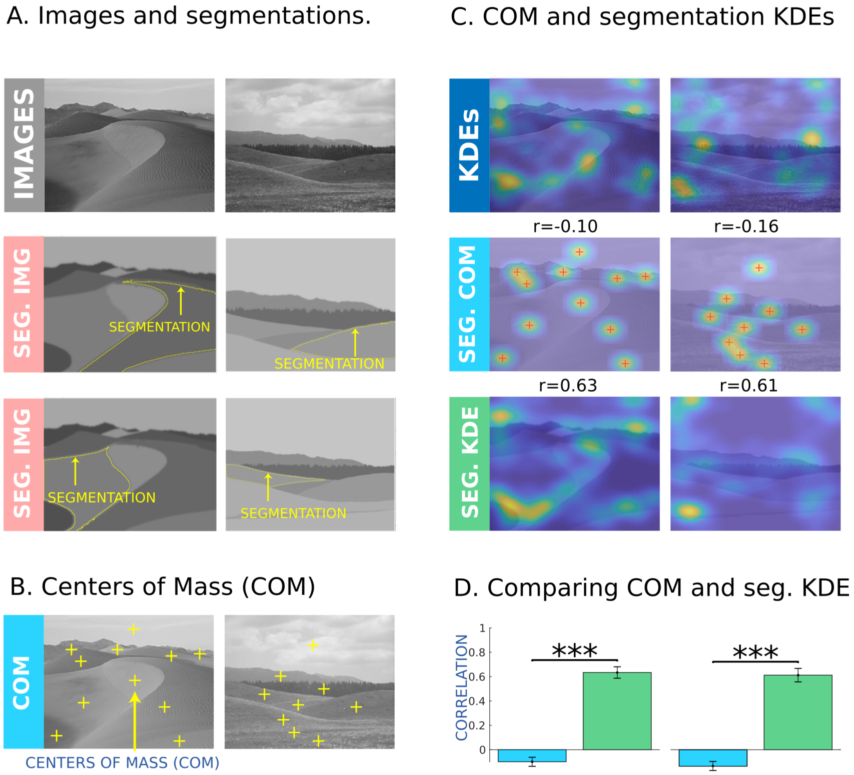

Fig. S16. Centers of Mass (COM) are poor predictors of spatial memory priors obtained via serial reproduction. A. Images were landscape images used in previous work, and segmentations were obtained using k-means clustering of the RGB values of the original color images, using the same procedure described by (12–14). Two examples of the segmentations are shown for both images. B. Centers of mass (COM) were computed by averaging the x and y coordinates of the pixels in each of the segmented regions. C. KDEs obtained using our serial reproduction results for the grasycale images as well as the segmentation images. Also shown are the COM maps obtained by smoothing the COM with an optimal smoothing parameter (maximizing its correlation to the KDEs obtained for the original grayscale images). D. Barplots showing the optimal performance of the COM maps, and the KDEs fit to the results from the serial reproduction chains using the segmentation images used for computing the centers of mass. Results clearly show that COM maps are poor predictors of the KDEs.

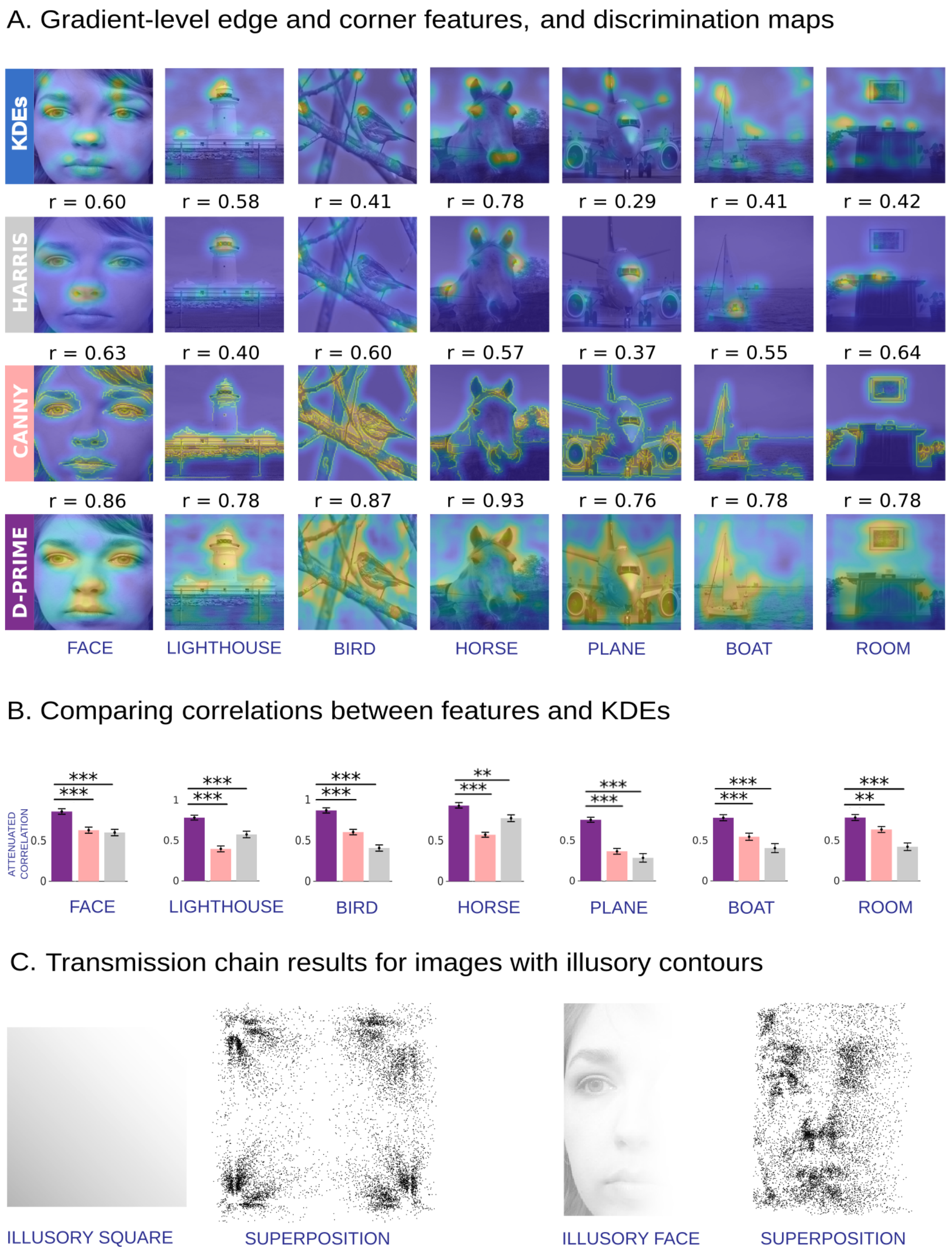

Fig. S17. Model comparison using attenuation. Discrimination accuracy maps are more predictive of the spatial memory KDEs than optimized corner (Harris) and edge (Canny) feature detectors implemented using (23). We performed a detailed grid search over the allowable parameter settings for both the Harris corner and Canny edge detectors and selected the settings that maximized the correlations to the KDEs. A. feature maps (with optimal smoothing) displayed over the images. Also shown are correlations with corrections for attenuation over each map. B. Barplots of the disattenuated correlations. Errorbars represent 1000 bootstrapped samples of the chain data. C. Chain results for a square with an illusory upper-right-hand corner and a face with an illusory right eye. Transmission-chain results reveal biases concentrated around the illusory regions: a pattern around the upper right-hand illusory corner of the square that is largely identical to the pattern we observe with the original image, as well as biases centered over the illusory eye in the face image.

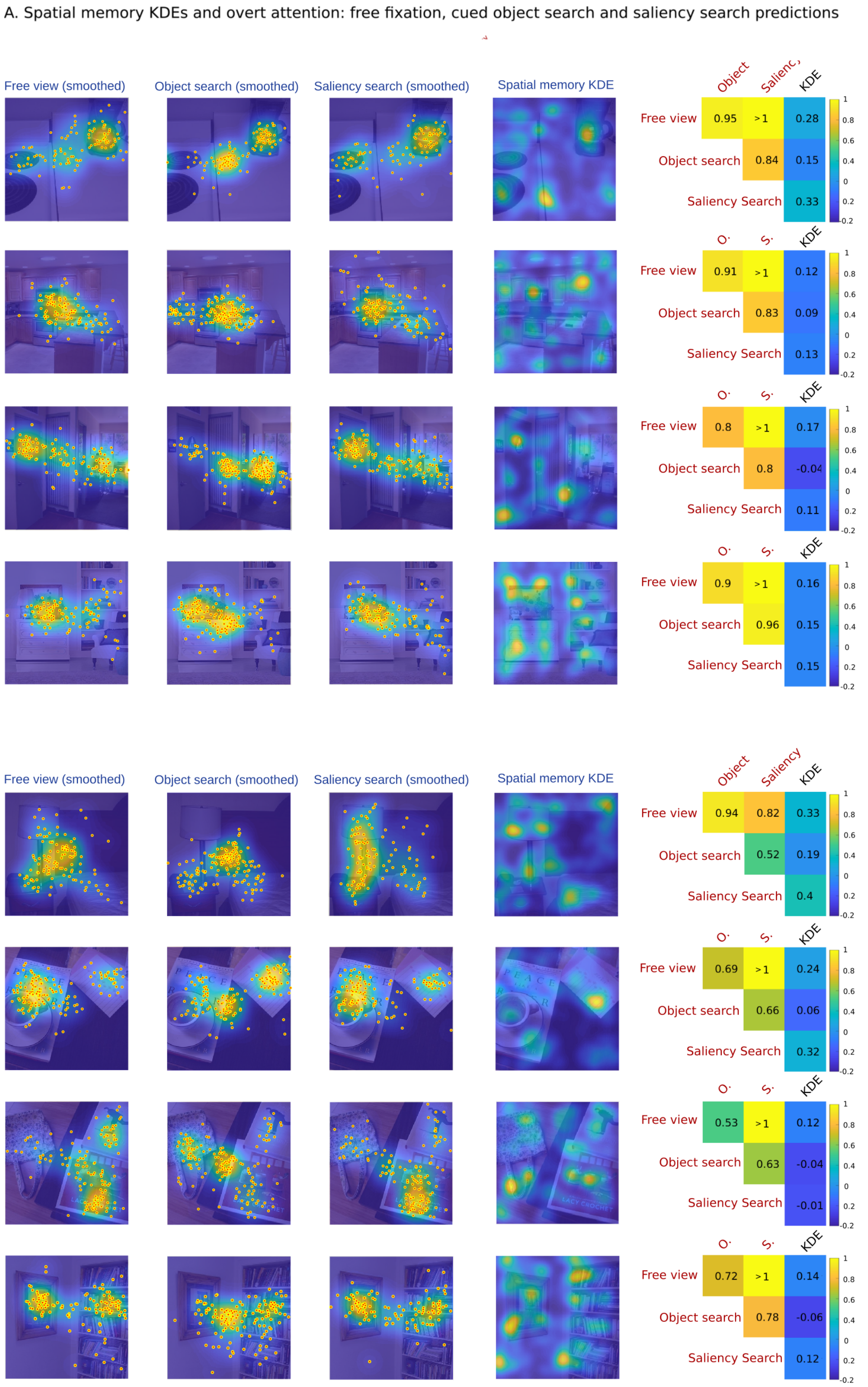

Fig. S18. Overt attention and spatial memory priors. Spatial memory KDEs, free fixations, cued object search fixations, and saliency search fixations. A. Fixation maps with optimal smoothing for free-fixation, cued object search, and saliency search tasks are not predictive of spatial memory priors. We show all the fixation maps and spatial memory KDEs for 8 images taken from the database used in (4). We applied a smoothing parameter to all the maps using Matlab's *imgaussfilt* function. We selected the smoothing parameter that maximized the correlations of the maps to the spatial memory KDEs. Also shown are correlation matrices with disattenuated correlations. We computed the disattenuated correlations using internal reliability estimates for each of the fixation maps and the chain data. We estimated the internal reliability by averaging the correlations between 100 split-half pairs of the data, for each of the predictors.

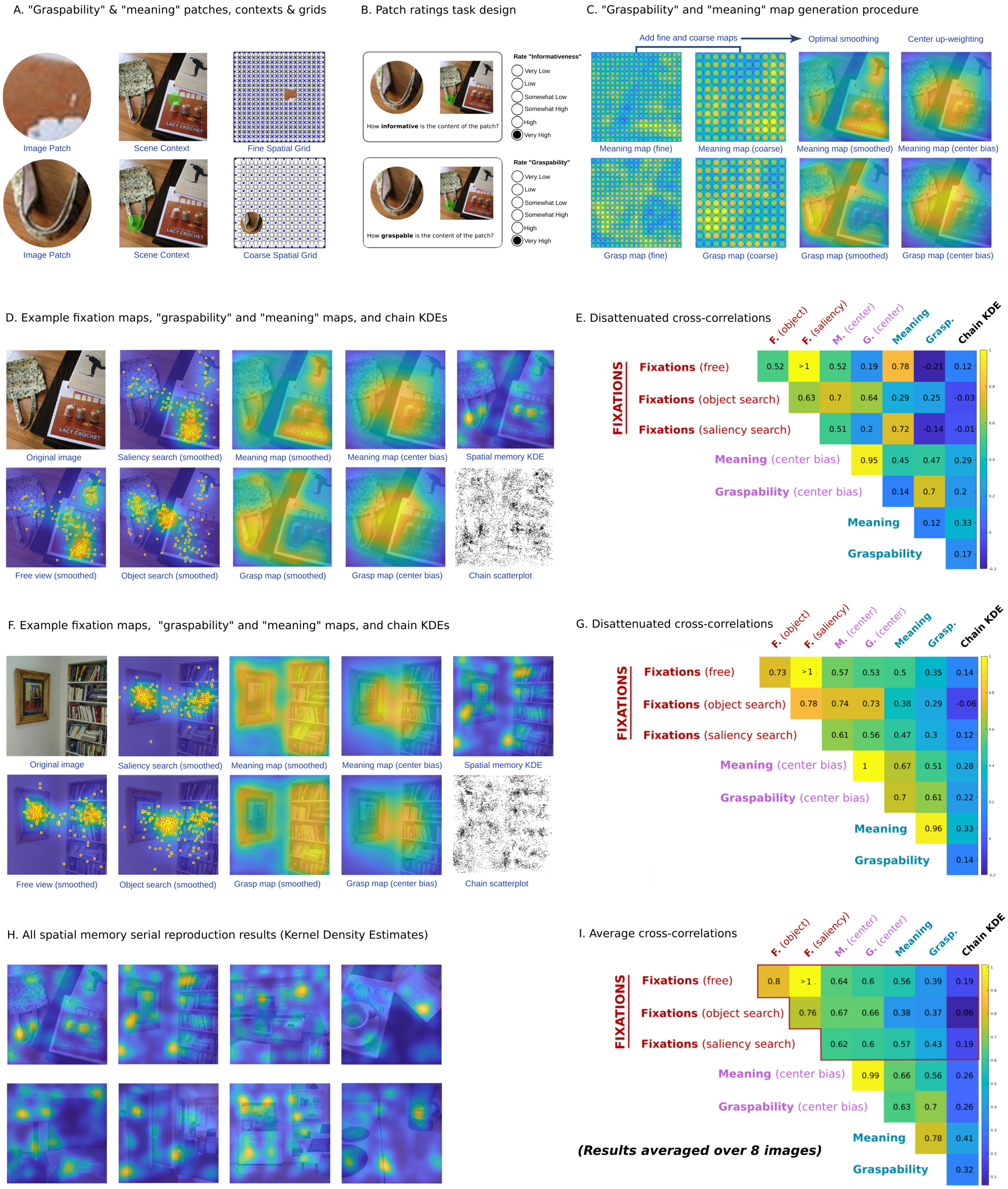

Fig. S19. Patch ratings experiments. We reproduced the experiments by (18, 19) for 8 images for which detailed eye-movement fixation data were available for free-viewing, cued object search and saliency search tasks (4). A. We extracted circular image patches from each of the images using fine and coarse spatial grids. B. We obtained 10 ratings of the "informativeness" and "graspability" for each of the patches on AMT in two separate experiments. C. For each experiment ("informativeness" vs. "graspability"), we averaged the ratings for each of the patches, and across fine and coarse scales. We then applied an optimal smoothing parameter (to maximize the correlation of the resulting maps to the KDEs). Finally, we down-weighted the edges of the maps to simulate the center bias in overt attention, using the same procedure as (18, 19). D. Example fixation maps, meaning and graspability maps, centered meaning and graspability maps, and KDE results. E. Disattenuated correlation matrix showing that neither the fixations, nor the meaning and graspability maps (with and without the center bias) are predictive of the spatial memory prior (Chain KDE) for the example shown in D. F-G shows another representative example, with similar results. H. All chain KDEs for the 8 images. I. Average correlation matrix with disattenuated correlations. Across all the images, neither the fixations, nor the meaning and graspability maps (with and without the center bias) are strongly predictive of spatial memory priors (Chain KDEs).

A.Transmission chain between-subject design results

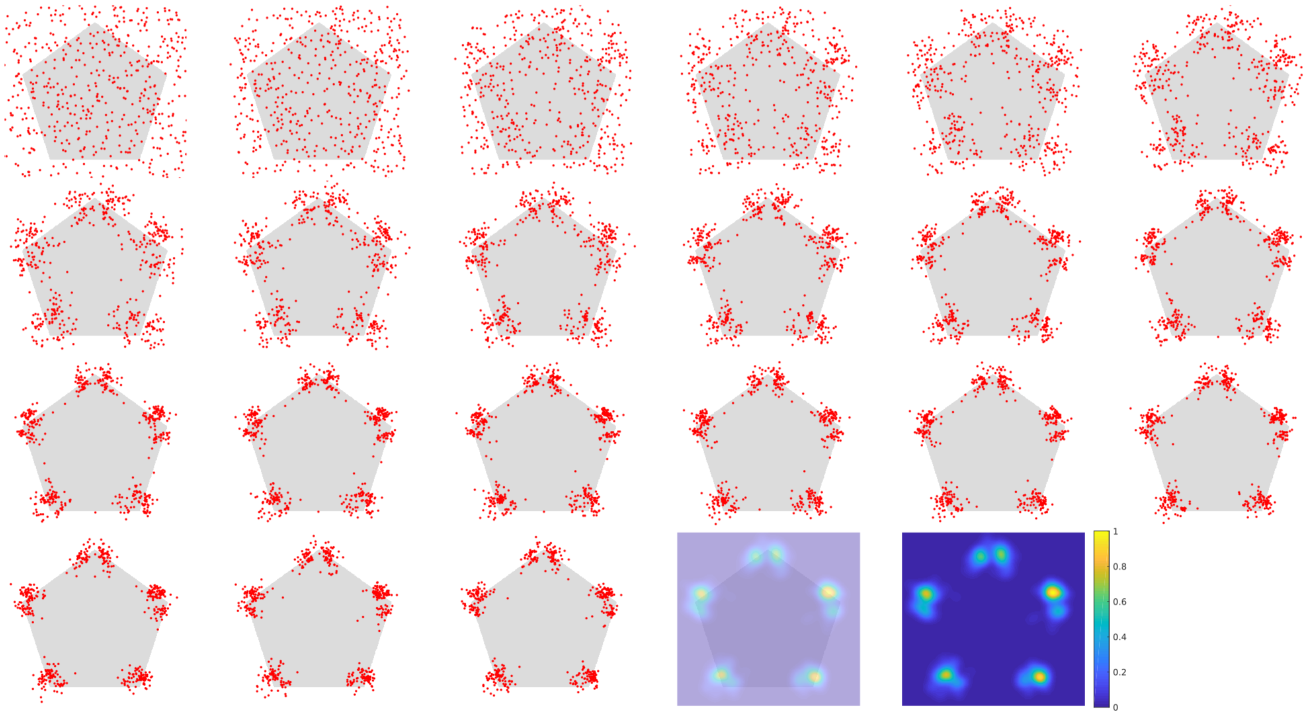

B.Transmission chain within-subject design results

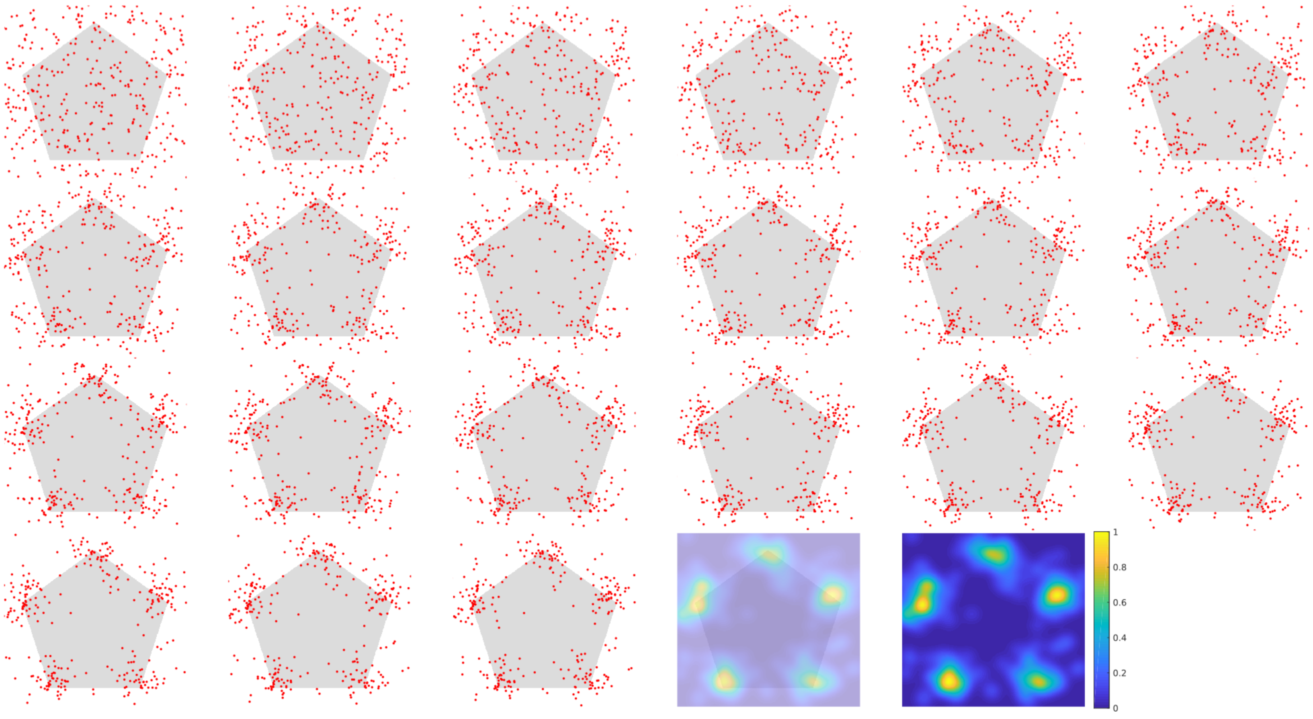

Fig. S20. Full serial reproduction results for a shaded pentagon (all chains and all iterations). A. Main results Using the between-subject design. B. Results using the within-subject design. The bottom right sub-panels show the parametric KDEs, and the next-to-last sub-panels show an overlay of the parametric KDEs over the image.

A.Transmission chain between-subject design results

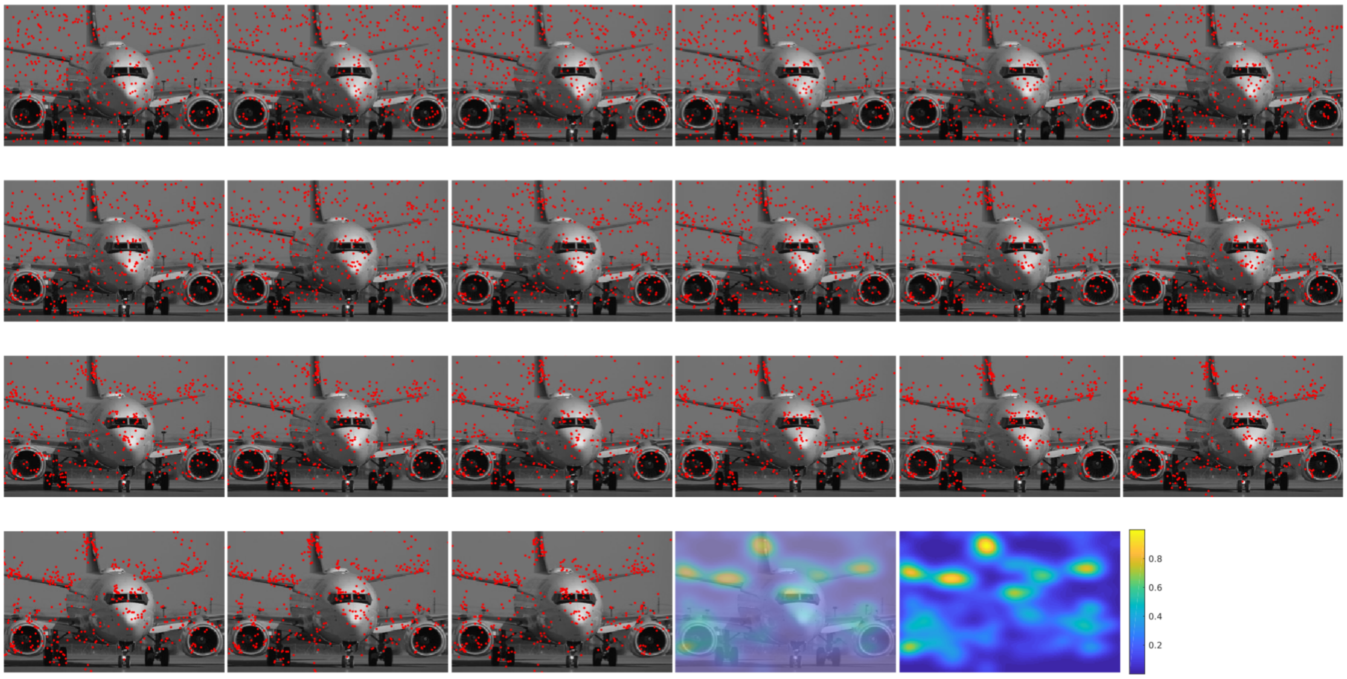

B.Transmission chain within-subject design results

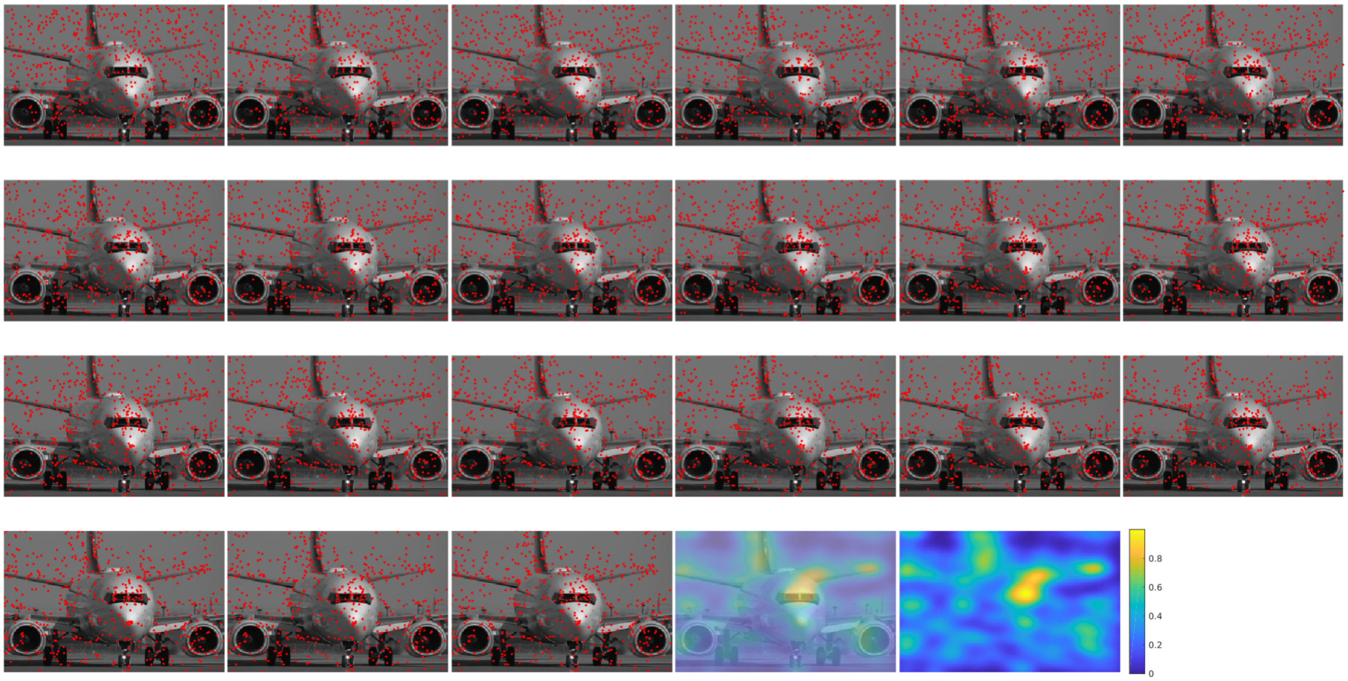

Fig. S21. Full serial reproduction results for a natural image (all chains and all iterations). A. Main results Using the between-subject design. B. Results using the within-subject design. The bottom right sub-panels show the parametric KDEs, and the next-to-last sub-panels show an overlay of the parametric KDEs over the image.

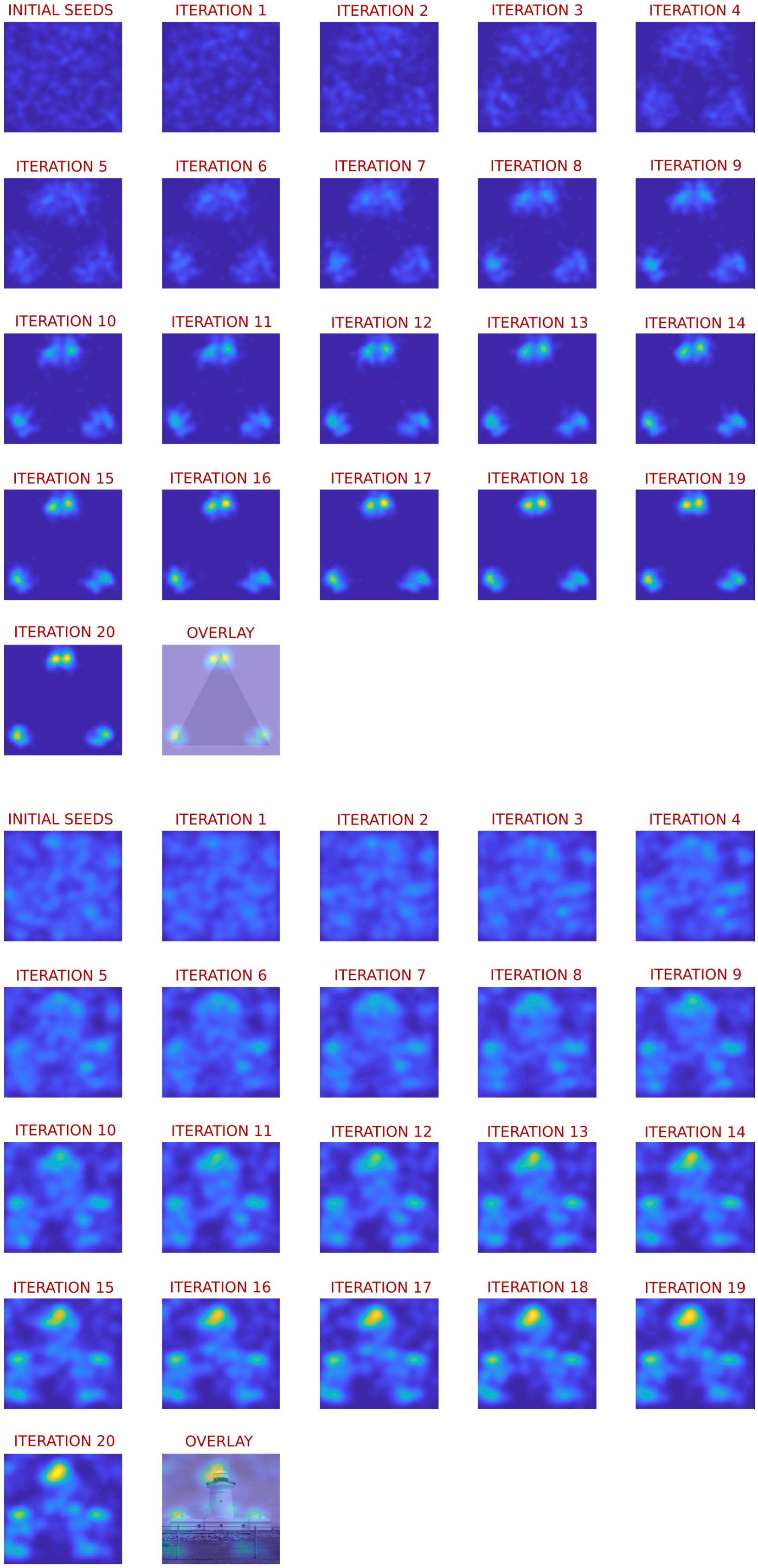

Fig. S22. Kernel density estimates (KDEs) for triangle and lighthouse image serial reproduction results. KDEs for the initial seed distribution and all 20 iterations of the chains are shown.

Experiment number	Image file name	Image category	Image width	Image height	Number of participants	Number of chains
1	gray-circle	Shape images	564	554	107	499
2	gray-triangle	Shape images	610	523	102	496
3	gray-square	Shape images	518	505	103	498
4	gray-pentagon	Shape images	512	486	103	494
5	face-with-illusory-contour	Natural images	192	256	94	498
6	Lighthouse	Natural images	166	256	121	500
7	Bird	Natural images	256	183	101	467
8	Horse	Natural images	192	256	104	483
9	Plane	Natural images	256	165	110	482
10	Boat	Natural images	256	192	103	478
11	Room	Natural images	172	256	106	485
12	Bird-segmentation	Segmented natural images	256	183	108	500
13	Horse-segmentation	Segmented natural images	192	256	106	488
14	Plane-segmentation	Segmented natural images	256	165	98	498
15	Boat-segmentation	Segmented natural images	256	192	105	492
16	Room-segmentation	Segmented natural images	172	256	106	491
17	"Face"	Discrimination experiments	292	277	178	N.A.
18	"Lighthouse"	Discrimination experiments	293	243	220	N.A.
19	"Bird"	Discrimination experiments	259	243	190	N.A.
20	"Horse"	Discrimination experiments	266	243	300	N.A.
21	"Plane"	Discrimination experiments	192	256	308	N.A.
22	"Boat"	Discrimination experiments	166	256	300	N.A.
23	"Room"	Discrimination experiments	256	183	240	N.A.
24	"gray circle from circle-to-square"	Discrimination experiments	192	256	240	N.A.
25	"gray triangle"	Discrimination experiments	256	165	280	N.A.
26	"gray square"	Discrimination experiments	256	192	240	N.A.
27	"gray pentagon"	Discrimination experiments	172	256	98	N.A.
28	gray-square-with-illusory-corner	Illusory contours	518	505	106	494
29	face-with-illusory-contour	Illusory contours	192	256	110	497
30	plane-200-ms-encoding	Natural images	256	165	117	481
31	plane-reduced-contrast	Natural images	256	165	106	500
32	plane-Gaussian-noise	Natural images	256	165	109	499
33	Plane-uniformity-manipulation	Natural images	256	165	210	492
34	Plane-payoff-manipulation	Natural images	256	165	110	499
35	Plane-delay-manipulation	Natural images	256	165	103	499
36	Pentagon-within-design	Shape images	512	486	74	288
37	Plane-within-design	Natural images	256	165	150	577
38	Fixations-image-1	Natural images	256	256	110	499
39	Fixations-image-2	Natural images	256	256	109	499
40	Fixations-image-3	Natural images	256	256	109	500
41	Fixations-image-4	Natural images	256	256	111	498
42	Fixations-image-5	Natural images	256	256	105	498
43	Fixations-image-6	Natural images	256	256	106	500
44	Fixations-image-7	Natural images	256	256	106	500
45	Fixations-image-8	Natural images	256	256	104	500
46	Fixations-image-1	Meaning ratings experiments	256	256	40	N.A.
47	Fixations-image-2	Meaning ratings experiments	256	256	40	N.A.
48	Fixations-image-3	Meaning ratings experiments	256	256	40	N.A.
49	Fixations-image-4	Meaning ratings experiments	256	256	40	N.A.
50	Fixations-image-5	Meaning ratings experiments	256	256	40	N.A.
51	Fixations-image-6	Meaning ratings experiments	256	256	40	N.A.
52	Fixations-image-7	Meaning ratings experiments	256	256	40	N.A.
53	Fixations-image-8	Meaning ratings experiments	256	256	40	N.A.
54	Fixations-image-1	Graspability ratings experiments	256	256	40	N.A.
55	Fixations-image-2	Graspability ratings experiments	256	256	40	N.A.
56	Fixations-image-3	Graspability ratings experiments	256	256	40	N.A.
57	Fixations-image-4	Graspability ratings experiments	256	256	40	N.A.
58	Fixations-image-5	Graspability ratings experiments	256	256	40	N.A.
59	Fixations-image-6	Graspability ratings experiments	256	256	40	N.A.
60	Fixations-image-7	Graspability ratings experiments	256	256	40	N.A.
61	Fixations-image-8	Graspability ratings experiments	256	256	40	N.A.
62	Lighthouse-300-ms-encoding	Natural images	166	256	115	499
63	Lighthouse-blank-probe	Natural images	166	256	107	500
64	Lighthouse-blank-probe-and-300-ms-encoding	Natural images	166	256	106	498
65	Blank-stimulus-and-probe-control	Natural images	200	256	53	250
66	Blank-stimulus-and-lighthouse-probe	Natural images	166	256	55	250
67	Lighthouse-300-ms-encoding-blank-probe-and-noise-masking	Natural images	166	256	110	500
68	Lighthouse-300-ms-and-noise-masking	Natural images	166	256	109	500
69	Landscape-image-1	Natural images	256	213	54	250
70	Landscape-image-2	Natural images	256	190	52	250
71	Landscape-image-1-segmentation	Segmented natural images	256	213	52	250
72	Landscape-image-2-segmentation	Segmented natural images	256	190	55	250
73	Shaded-19-sided-polygon-(300ms-presentation)	Shape images	486	486	113	475
74	Shaded-3-sided-polygon	Shape images	487	487	89	405
75	Shaded-4-sided-polygon	Shape images	488	488	106	495
76	Shaded-5-sided-polygon	Shape images	489	489	105	496
77	Shaded-7-sided-polygon	Shape images	490	490	106	498
78	Shaded-9-sided-polygon	Shape images	491	491	106	494
79	Shaded-11-sided-polygon	Shape images	492	492	101	485
80	Shaded-13-sided-polygon	Shape images	493	493	104	489
81	Shaded-15-sided-polygon	Shape images	494	494	107	498
82	Shaded-17-sided-polygon	Shape images	495	495	107	497
83	Shaded-19-sided-polygon	Shape images	496	496	107	490
84	Shaded-21-sided-polygon	Shape images	497	497	110	498
85	Shaded-25-sided-polygon	Shape images	498	498	107	492

Fig. S23. All experiments, including the image names and categories, the width and heights of all stimulus images, the total number of participants who participated in each experiment, and the number of chains where applicable.
